# Driving Forces and Spin-Gated Reactivity of N5-Hydropyrimidopteridinetetraone Radicals in Photocatalysis

**DOI:** 10.1021/acs.joc.6c00017

**Published:** 2026-04-15

**Authors:** Zohreh Amanollahi, Luisa Zach, Tobias Taeufer, Thanh Huyen Vuong, Olga S. Bokareva, Jola Pospech

**Affiliations:** † 28392 Leibniz Institute for Catalysis, Albert-Einstein-Str. 29a, Rostock 18059, Germany; ‡ University of Rostock, Interdisciplinary Faculty Life, Light & Matter, Albert-Einstein-Str. 25, Rostock 18059, Germany; § University of Rostock, Institute of Chemistry, Albert-Einstein-Str. 27, Rostock 18059, Germany

## Abstract

Pyrimidopteridinetetraones **(PPT**s) are potent excited-state oxidants that also function as hydrogen atom transfer (HAT) catalysts. Here, we investigate the photobasicity and mechanistic role of N-hydropyrimidopteridinetetraone radicals (**PPTH** ^•^) in catalyst turnover. Optical and electron paramagnetic resonance (EPR) spectroscopy, quantum chemical calculations, and thermochemical analysis reveal that excited-state protonation of **PPT**s occurs at the N5 position of the heterocycle, generating **PPTH** ^•^ as a key catalytic intermediate. Formation of the exocyclic N–H bond from the excited state is associated with a bond dissociation free energy (BDFE) of 131 kcal mol^–1^ from the singlet and 106 kcal mol^–1^ from the triplet excited state, while subsequent homolytic N–H cleavage (BDFE ≈ 57 kcal mol^–1^) enables catalyst regeneration. Spin-resolved mechanistic analysis shows that singlet radical encounters proceed uniformly via a direct HAT (dHAT) pathway across aliphatic, benzylic, and redox-active substrates with low activation free energies. In contrast, triplet encounters access substantially higher-energy reaction manifolds and can give rise to substrate-dependent mechanistic trajectories, including dHAT, concerted proton-coupled electron transfer (cPCET), or stepwise electron and proton transfer sequences. These findings establish a spin-state–dependent framework for HAT in **PPT** photoredox catalysis, highlighting how electron affinity and (photo)­basicity govern excited-state reactivity and positioning **PPT**s as a platform for proton-coupled electron transfer chemistry.

## Graphical abstract

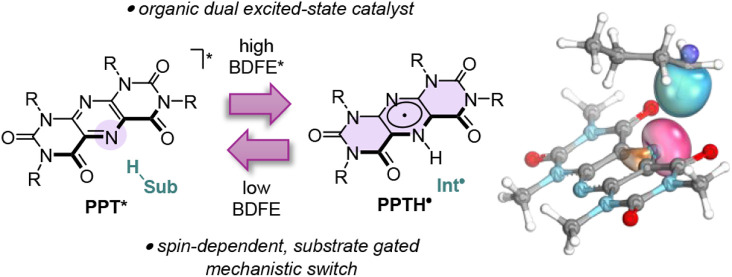

## Introduction

A detailed understanding of mechanistic paradigms in photocatalysis is essential for the rational design of next-generation photocatalysts and the expansion of their synthetic utility.^1,2^ While photoredox catalysis has enabled a wide range of bond-forming transformations under mild conditions,^3–5^ its success often hinges on complex, multistep processes involving excited-state electron transfer, energy transfer,^6–8^ and hydrogen atom transfer (HAT) events.^9,10^ A crucial advantage of proton-coupled electron transfer (PCET)-based systems is the expansion of the scope of light-driven transformation beyond a single-electron transfer (SET)-dependent reaction regime that solely relies on matching redox potentials (Figure 1A and B). Dissecting these pathways, particularly distinguishing between direct HAT (dHAT) and PCET^11,12^ is critical for predicting reactivity, tuning selectivity, and optimizing catalyst performance. In dHAT, the substrate–H bond undergoes homolytic cleavage, and the proton and electron are transferred together as a neutral hydrogen atom to the same acceptor orbital of the reacting species and represent a true H atom abstraction event. Conversely, cPCET also proceeds in a single kinetic step but involves spatially separated proton and electron motion, such that H^+^ and e^–^ are delivered to different acceptors along a coupled reaction coordinate.^13^ Productive PCET and HAT reactivity is controlled by the combined proton transfer (PT) and electron transfer (ET) thermodynamics (p*K*_a_ values, and redox potentials) and is thus fundamentally governed by differences in bond dissociation free energies (ΔBDFEs) (Figure 1C and D).^14^ Appreciating their mechanistic distinction is essential for interpreting reactivity patterns within photoredox manifolds and for rationally engineering catalysts capable of favoring one pathway over the other.

**Figure 1:**
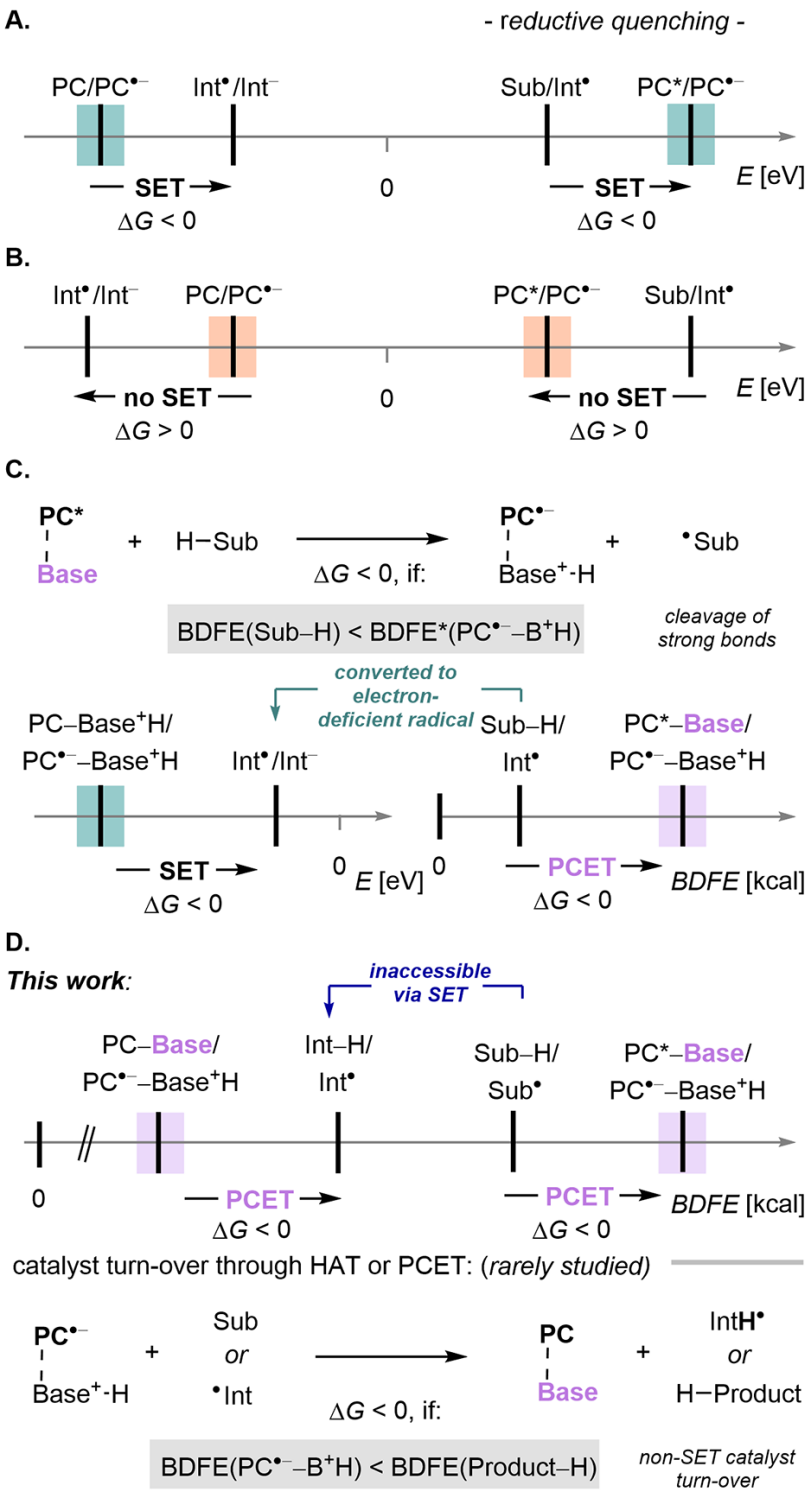
Decisive thermodynamic measures for excited-state oxidants: A. Productive photoredox catalysis via SET. B. Unfavorable redox couples for SET. C. PCET/SET or HAT/SET-based catalytic cycles. D. Fully PCET or HAT-based catalytic cycles (this work).

PCET, a mechanism ubiquitous in biological systems,^15,16^ has seen increasing application in synthetic organic chemistry over the past decade.^14,17^ The judicious selection of oxidant and Brønsted base pairs is key to achieving selective PCET, yet the success of multisite PCET often depends on overcoming the unfavorable kinetics of multimolecular interactions. These obstacles are addressed by implementing recent reports by Mayer,^18–21^ Wenger,^22,23^ and a collective account by Scholes, Hammes-Schiffer, and Knowles^24,25^ have demonstrated the impact of preassociation between redox-active species and Brønsted acids or bases in facilitating efficient excited-state PCET by reducing the reaction’s molecularity (Figure 2A).^26,27^ In 2009, Kojima, Mayer, and Fukuzumi provided insights into PCET mechanisms in the context of ruthenium­(III)–pterin complexes, elucidating how these complexes facilitate the simultaneous uptake of protons and electrons.^28–30^ In 2023, Ritter and coworkers demonstrated how protonation can significantly influence the excited-state oxidation potentials of acridinium photoredox catalysts in the context of hydrofunctionalization reactions.^31,32^ Building on related concepts, Barham and coworkers exploited the driving force associated with N–H bond formation in similar systems to enable the activation of C­(sp^3^)–H bonds.^33^ Earlier, García-Mancheño and Ooi had showcased the ability of twisted diradicals, generated from zwitterionic acridinium amidates, to mediate C­(sp^3^)–H bond activation.^34^ Recently, Yoshihiko Hamashima and coworkers demonstrated that direct hydrogen atom transfer between carboxylic acids and the triplet state of Xanthone can occur despite the presence of more readily activated benzylic C–H bonds, highlighting the importance of preassociation in governing O–H HAT selectivity.^35^

**Figure 2:**
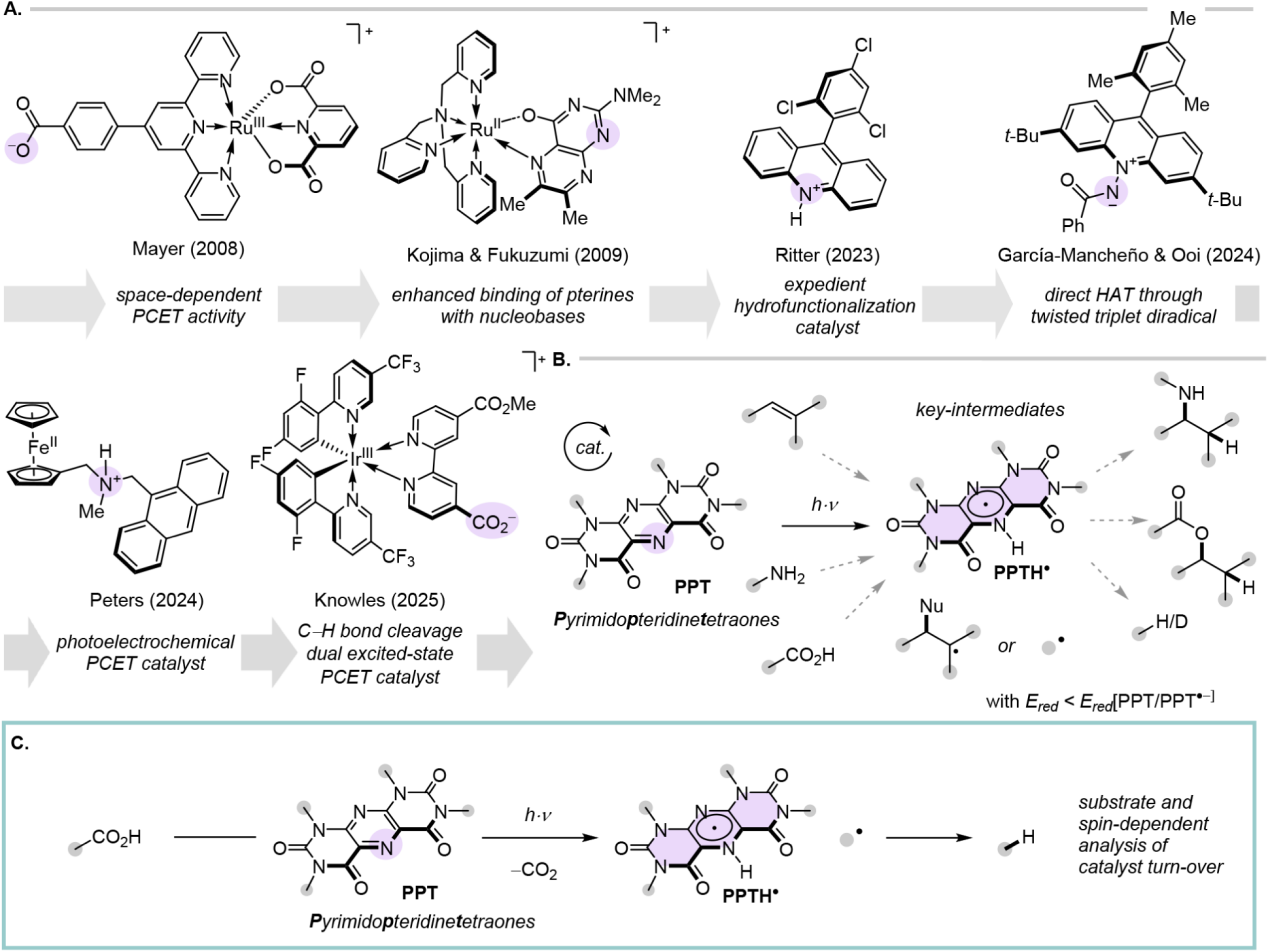
A. Selected literature examples for the covalent tethering of excited-state oxidants and bases in the context of excited-state PCET. B. Examples of **PPT** photocatalysis harnessing PCET/HAT reactivity. C. This work: Full spectrum elucidation of **PPTs** HAT vs PCET reactivity via **PPTH** ^•^ as a key intermediate.

In biological systems, flavins prominently exemplify how distinct protonation and redox states are exploited to initiate redox events and facilitate proton-coupled electron transfer,^36,37^ serving as profound inspiration for synthetic organic chemists.^38,39^

Foundational studies have established thermodynamic and kinetic frameworks for understanding dHAT and (c)­PCET, correlating reactivity with bond dissociation energies, redox potentials, and proton affinities.^12^ Understanding how excited-state properties govern the formation of catalytically relevant radical intermediates is essential, particularly as the modulation of redox potentials via pH-dependency becomes more prevalent in photocatalytic systems.^13,40,41^ However, while bifunctional catalysts enable challenging X–H bond activation via PCET, most studies limit their use to radical generation, with the resulting radical species captured through radical addition to electron-deficient alkenes (Figure 1C). PCET efficiently cleaves strong bonds under mild conditions, but subsequent radical addition to electron-poor alkenes provides a fast general pathway for C–C bond formation and allows catalyst turnover via a SET manifold, avoiding the need for a second PCET step. Consequently, the field has largely relied on PCET for activation coupled to classical radical addition/SET sequences for product formation. In contrast, examples focusing on the conversion of the bifunctional catalyst under non-SET conditions are scarce (Figure 1D). A notable exception is the photoelectrochemical reduction of ketones reported by Peters and coworkers, in which both the initial activation of the catalyst and the subsequent reconversion of the catalyst proceeds via PCET, mediated by a ferrocene/alkylamino Brønsted base combination linked to an anthracene photosensitizer.^42^ This example illustrates that bifunctional photocatalysts can operate exclusively through PCET steps, highlighting the mechanistic diversity accessible in bifunctional photocatalytic systems.

We have introduced pyrimidopteridines (**PPT**), previously a stoichiometric side-product from photomediated deoxygenation of their corresponding *N*-oxides,^43,44^ as potent organic photoredox catalysts.^45^ These catalysts have been shown to convert radicals beyond the SET manifold in various examples (Figure 2B).^46–48^ In the course of our studies, we have observed a pronounced counter-thermodynamic catalyst turnover involving radical intermediates with reduction potentials more negative than that of the **PPT**/**PPT** ^•–^ redox couple $(E_{1/2}^{red}\left(PPT/PPT^{•-}\right)=-1.21$ V vs SCE in MeCN),^49^ occurring in the absence of any assisting cocatalysts or sacrificial reagents. This unexpected catalyst turnover was rationalized by the formation of a reactive N5-hydropyrimidopteridinetetraone radical (**PPTH** ^**•**^) species, as confirmed by electron paramagnetic resonance (EPR) spectroscopy. The **PPTH** ^•^ radical species acts as a formal HAT reagent, enabling product formation and catalyst regeneration.^48^ As these catalysts merge potent organic photoredox catalysis with the emerging desire for dual reactivity, understanding both the formation pathways and subsequent conversion of this **PPTH** ^•^ intermediate was deemed critical to fully elucidate the photocatalytic cycle and harness the full potential of **PPT** photoredox catalysis in future accounts.

In this study, we investigate how photobasicity and excited-state thermodynamics govern both N–H bond formation and cleavage in intermediary **PPTH** ^•^ radicals (Figure 2C).^50^ A combination of optical spectroscopy, EPR, and quantum chemical calculations is employed to delineate the substrate-gated thermodynamic boundary between direct HAT and PCET pathways from **PPTH** ^**•**^, through analysis of intrinsic bonding orbital (IBO) evolution along the intrinsic reaction coordinate (IRC).^51^ IRC denotes the minimum-energy reaction pathway connecting reactants, transition states, and products, while IBOs provide a localized orbital representation that facilitates tracking bond reorganization along this pathway. These findings establish a comprehensive thermodynamic framework for HAT in **PPT** photoredox catalysis and broaden our understanding of excited-state reactivity in organic systems that are both redox-active and proton-affine.

## Results and Discussion

We commenced our investigation by characterizing the photophysical properties of the **PPT** catalyst and its key **PPTH** ^**•**^ intermediate to gain deeper insight into N–H bond formation and cleavage. We focused on determining the (i) excited-state energies, (ii) excited-state redox potentials, and (iii) ground- and excited-state p*K*_a_ values, which collectively allowed us to calculate the BDFEs of the exocyclic N–H bond for both its formation in the excited state and its cleavage during catalyst regeneration. Tetrapropylpyrimidopteridinetetraone (**PrPPT**) was used in all measurements due to its convenient synthetic accessibility,^49^ ease of purification, good solubility, and consistent performance across catalytic transformations.^44–48^ **PPTs** demonstrate consistent photophysical and electrochemical properties, independent of their *N*-substituents and are therefore collectively abbreviated as **PPT** in this account.

The results of this study are summarized in a box-shaped multidimensional square-scheme formalism depicted in Figure 3. The excited-state energy of **PPT** (*E*_S_1__(**PPT** ^*^) = +3.31 eV) was determined from UV–vis absorption and emission spectroscopy and was used to benchmark the calculated singlet and triplet excited-state energy (*E*_T_1__(**PPT** ^*^) = +2.10 eV), obtained from (Time-Dependent)­Density Functional Theory, (TD)­DFT, calculations at the CAM-B3LYP/def2-TZVP level of theory (see Supporting Information for more details and optimized geometries). Ground-state redox potentials were determined by cyclic voltammetry (CV) and differential pulse voltammetry (DPV), enabling calculation of the triplet excited-state reduction potential Ered*(P3PT*/PPT•−)=+1.15 V vs NHE in MeCN. All electrochemical potentials reported in this study are referenced to the normal hydrogen electrode (NHE), following the recommendations of Agarwal and Mayer, to ensure the most mechanistically sound interpretation of thermodynamic parameters.^12^

**Figure 3:**
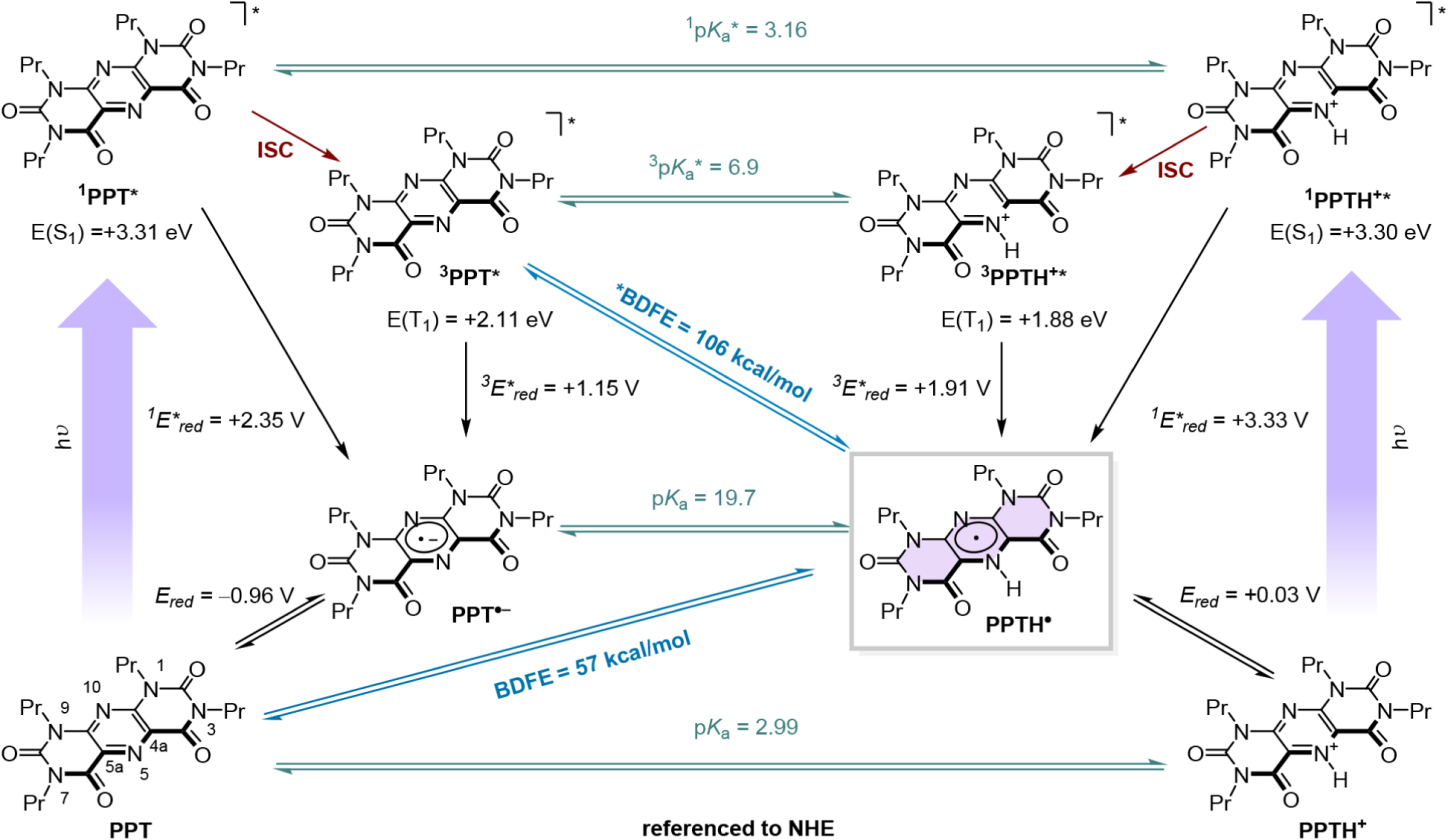
Thermochemical box scheme for **PPT** and **PPTH^+^** illustrating the interconnection of excited-state energies, ground- and excited-state redox potentials, and acid–base equilibria to bond dissociation free energies. ^1^BDFE of 131 kcal mol^–1^ is not shown for clarity.

The p*K*_a_ of **PPTH** ^+^ was computed by using a DFT-based thermodynamic cycle that relates the free energy difference (Δ*G*) between protonated and deprotonated species in solution to reference acids of known p*K*_a_, yielding a predicted value of 2.93. This result was experimentally validated by titration using differential absorption spectroscopy, which provided an experimental p*K*_a_ of 2.99 for the protonated catalyst **PrPPTH** ^+^, which is in excellent agreement with theory. Subsequently, ground-state redox potentials and excited-state characteristics were determined. Notably, protonation had a negligible effect on the singlet excited-state energy (*E*_S_1__(**PrPPTH** ^+*^) = +3.30 eV), yet significantly shifted the ground-state reduction potential to more positive values $(E_{1/2}^{red}\left(PPTH^{+}/PPTH^{•}\right)=+0.03$ V), corresponding to a proton-coupled shift Δ*E*_red_ of +0.99 eV. This increase in electron affinity directly influences the excited-state reduction potentials, rendering them substantially more positive compared to the unprotonated form (Ered*(P1PTH+*/PPTH•)=+3.33 V and Ered*(P3PTH+*/PPTH•)=+1.91 V vs NHE in MeCN). The computed intersystem-crossing (ISC) rate constants from S_1_ to T_2_ are 5.4 × 10^8^ s^–1^ and 3.2 × 10^9^ s^–1^ for **MePPT** and **PrPPT**, respectively, corresponding to characteristic ISC times of approximately 1.8 and 0.3 ns. In comparison, the calculated radiative rate constants from S_1_ are 7.6 × 10^8^ s^–1^ for **MePPT** and 7.1 × 10^8^ s^–1^ for **PrPPT**, corresponding to radiative lifetimes of about 1.3 and 1.4 ns, respectively, whereas the experimentally measured S_1_ lifetime is about 3 ns.^49^ We emphasize that the ISC rate is governed by spin-vibronic coupling^52^ and by the accessibility of crossing regions on the excited-state surfaces; therefore, the vertical S_1_–T_2_ energy gap shown in Figure 5 (*vide supra*) is not, by itself, the quantity that determines the ISC kinetics. These results indicate that the ISC can compete efficiently with fluorescence and other S_1_ decay channels. This competition is especially pronounced for **PrPPT**, for which ISC is significantly faster than fluorescence and is therefore expected to redirect a substantial fraction of the excited-state population into the triplet manifold. Under typical photocatalytic concentrations, diffusion-controlled electron transfer can still reach effective rates on the order of 1 × 10^8^–10^9^ s^–1^; thus, for **MePPT**, a productive SET directly from S_1_ remains kinetically accessible. Overall, these results suggest that the productive SET step depends on the substitution pattern: **MePPT** may plausibly react from the singlet excited state, whereas **PrPPT** is more likely to involve the triplet manifold because of its markedly faster ISC.

To determine the BDFEs for the N–H bond in the key **PPTH** ^•^ intermediate, the proton and electron transfer steps leading to the **PPTH** ^•^ radical from the excited and the ground states were connected through a square-scheme formalism, drawing a comprehensive picture of the thermochemical interplay of **PPT** and **PPTH** ^+^ (Figure 3). The generation of the **PPTH** ^•^ radical from the ground state, while thermodynamically accessible, requires forcing conditions with either the use of strong acids (p*K*_a_ < 3.00 in MeCN) or highly reducing agents (*E*_red_ < −0.96 eV vs NHE in MeCN). The N–H bond BDFE of 57 kcal mol^–1^ was both calculated based on the associated square scheme (Figure 3) using the Bordwell formalism and computationally determined with ethylbenzene and cyclohexane as a reference and proved to be in good agreement. The obtained ground-state BDFE enables calculation of a p*K*_a_ of 19.7 for **PPTH** ^•^, indicating that the radical anion **PPT** ^•–^ is significantly basic. The excited-state acidity (p*K*_a_*) was estimated using the Förster formalism,^53,54^ yielding values of 3.16 in the singlet excited state (S_1_) and 6.9 in the triplet excited state (T_1_). The high excited-state bond dissociation free energy of formation (BDFE*) of 131 kcal mol^–1^ from the singlet excited state arises from the strongly oxidizing excited-state reduction potential (Ered*(P1PTH*/PPT•−)=+2.35 V vs NHE) combined with the high basicity of the reduced form of the heterocycle. From the protonated catalyst, the BDFE is governed by its exceedingly high excited-state reduction potential (>3.0 V).

The relationship between the strong basicity of **PPT** ^•–^ and the moderate reduction potential of triplet-state **PPT** (Ered*(P3PT*/PPT•−)=+1.15 V vs NHE), or conversely, the moderate photobasicity and strongly oxidizing character of the triplet-state **PPTH** ^+^ Ered*(P3PTH+*/PPTH•)=+1.88 V vs NHE in MeCN), similarly results in a highly favorable driving force for the formation of **PPTH** ^•^. This finding is reflected in an excited-state bond dissociation free energy (^3^BDFE*) of 106 kcal mol^–1^.

Taken together, these thermodynamic relationships explain why **PPTH** ^•^ readily forms from PPT*. Photoexcitation renders the formation of the N–H bond in **PPTH** ^•^ highly favorable, providing the thermodynamic driving force for hydrogen atom transfer.

Next, we sought to validate the thermodynamic parameters summarized in Figure 4 experimentally. EPR spectroscopy revealed the rapid formation of the characteristic signal corresponding to the **PPTH** ^•^ radical upon irradiation of a solution containing the photocatalyst **PPT** in acetic acid in MeCN (p*K*_a_ = 23.5,^55^ *E*_ox_ = +2.55 V vs NHE^56^) (Figure 4A). The spectrum exhibits a distinctive hyperfine splitting pattern arising from interactions with two nonequivalent nitrogen atoms and one hydrogen atom (*A*_N1_ = 7.91 G, *A*_N2_ = 3.69 G, and *A*_H_ = 7.46 G; Figure 4B), consistent with the proposed radical structure. The position of the N–H bond was determined by DFT calculations, which revealed that protonation on the carbonyl-containing *ONO* face (see SI, Figure S4 and Table S7) is thermodynamically favored by 16 kcal mol^–1^ over protonation on the nitrogen-rich *NNN* face. With both electron and proton transfers individually unfavorable, the cPCET pathway is most plausible, supported by an overall ΔBDFE* of 25 kcal mol^–1^ from the singlet and 3.0 kcal mol^–1^ from the triplet excited state (Figure 4C).

**Figure 4:**
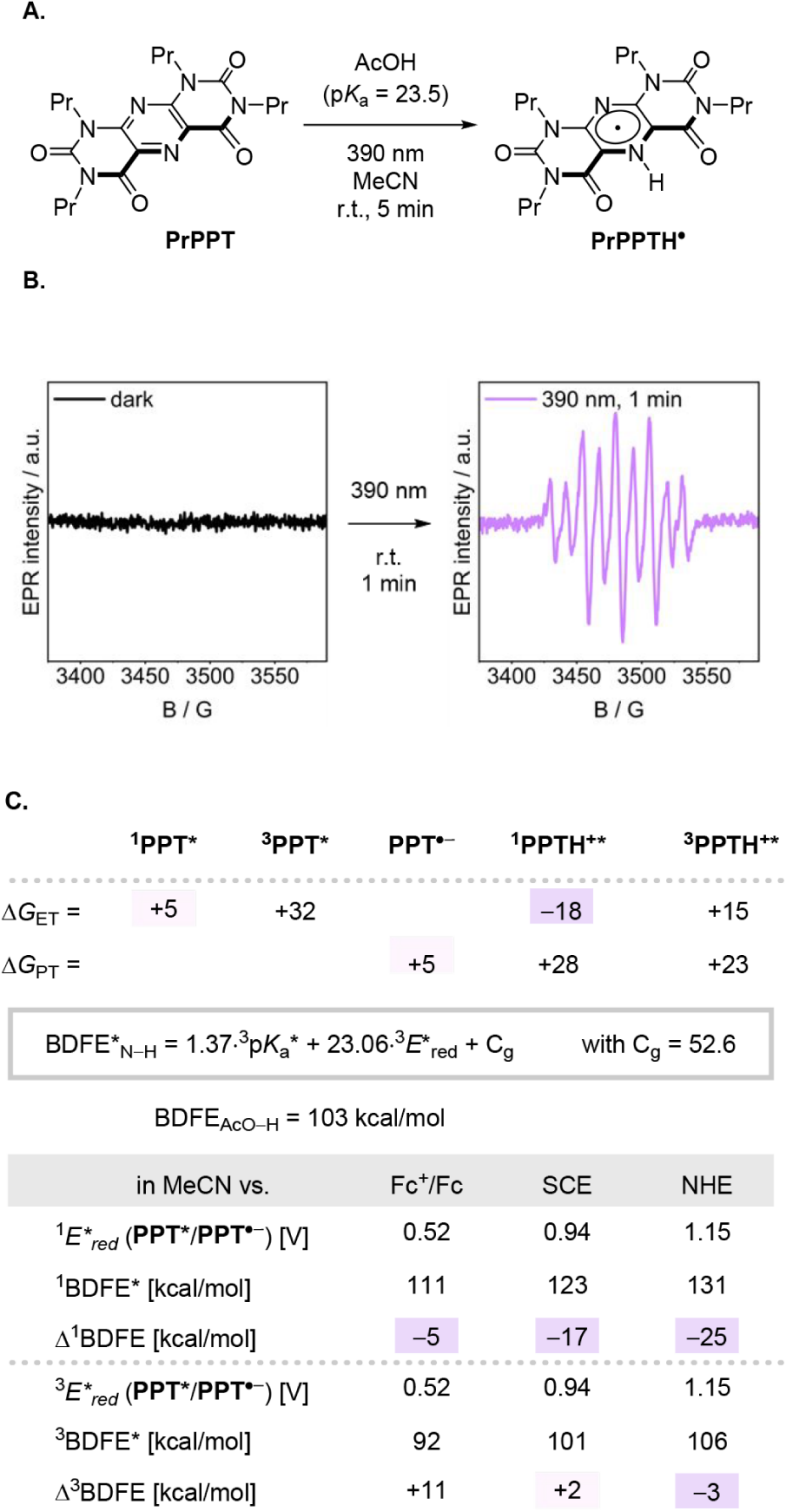
A. Reaction scheme for the photomediated formation of **PrPPTH^•^** from a weak acid. B. EPR spectrum of **PrPPTH^•^**. The simulated spectrum of the reaction according to Figure 4A reveals hyperfine splitting consistent with two nonequivalent nitrogen atoms and one hydrogen atom (*A*_N1_ = 7.91 G, *A*_N2_ = 3.69 G, *A*_H_ = 7.46 G). C. Thermochemical comparison of stepwise ET or PT pathways versus a cPCET mechanism.

The thermodynamic parameters obtained for **PPT** indicate that its excited state provides sufficient driving force to facilitate homolytic bond cleavage via PCET, involving bonds weaker than 106 kcal mol^–1^. Concurrently, scission of the exocyclic N–H bond in **PPTH** ^•^ (BDFE = 57 kcal mol^–1^) is thermodynamically favorable when forming stronger bonds, such as benzylic C–H (85–88 kcal mol^–1^)^57^ or aliphatic C–H bonds (≈96 kcal mol^–1^),^58^ enabling efficient HAT. Notably, the hydrogen-donating ability of **PPTH** ^•^ compares well with related flavin hydroquinones and flavin semiquinones (60–64 kcal mol^–1^)^12^ as well as other established HAT reagents, including thiols (S–H BDFEs = 79–87 kcal mol^–1^),^59^ dihydropyridines (C–H BDFEs ≈ 73–77 kcal mol^–1^),^60^ and metal polypyridyl carboxylate complexes (43–56 kcal mol^–1^).^24^ Thus, the intrinsic reactivity of **PPTs** holds great promise in organic photoredox catalysis and offers key features for sustainable, operationally simple photoredox transformations, eliminating the need for sacrificial HAT reagents or cocatalysts.

To strengthen the mechanistic rationale for PPTs in organic photoredox catalysis, we investigated their key characteristics via computational methods. The rigid framework of PPTs allows (TD)­DFT calculations to predict their excited-state properties reliably. Vertical excitation energies reproduce the small Stokes shift, reflecting a narrow energetic distribution, as shown by the minor differences between vertical and adiabatic excitation energies (Figure 5A). The Jablonski diagram depicts the computed TDDFT energy levels along with the suggested initial excited-state evolution to the bright S_1_ state, followed by the vibrational relaxation (VR) to the corresponding minimum, intersystem crossing (ISC) to the triplet manifold, most likely to the T_2_ due to the largest spin–orbit coupling (SOC_S_1_→T_2__ = 2.73 cm^–1^). This process is followed by internal conversion (IC) to the lowest triplet state, which is involved in further catalysis. The natural transition orbitals (NTO) analysis^61^ reveals a pronounced spatial separation between the highest occupied NTO (HONTO) and lowest unoccupied NTO (LUNTO), indicating an intramolecular charge transfer (CT) from the electron-rich *NNN* face to the electron-deficient *ONO* face (Figure 5B). This redistribution increases the electron density at N5, enhancing its proton affinity in the excited state (Δp*K*_a_ = 3.91). Fragmentation analysis of the **PrPPT** core revealed strict compartmentalization of the molecule’s excited-state landscape, based on electron–hole correlations within the computed set of excited states.^62^ This partitioning is identical for both singlet and triplet manifolds and clearly separates the **PPT** backbone from all four propyl chains. When the threshold for clustering is slightly lowered, the propyl chains are further divided into two correlated fragments: one pair is located on the *NNN* face and another on the *ONO* face. This observation is further supported by representative shapes of the NTOs involved in the lowest energy transitions, where the electron density is exclusively delocalized over the **PPT** core. The spatial separation of the side chains supports the conclusion that substituent modifications can be introduced without significantly affecting the excited-state redox potentials. This conclusion is consistent with previous spectroscopic and electrochemical studies.^49^ Consequently, most calculations were performed using the tetramethyl-substituted **PPT** derivative to minimize the computational cost.

**Figure 5:**
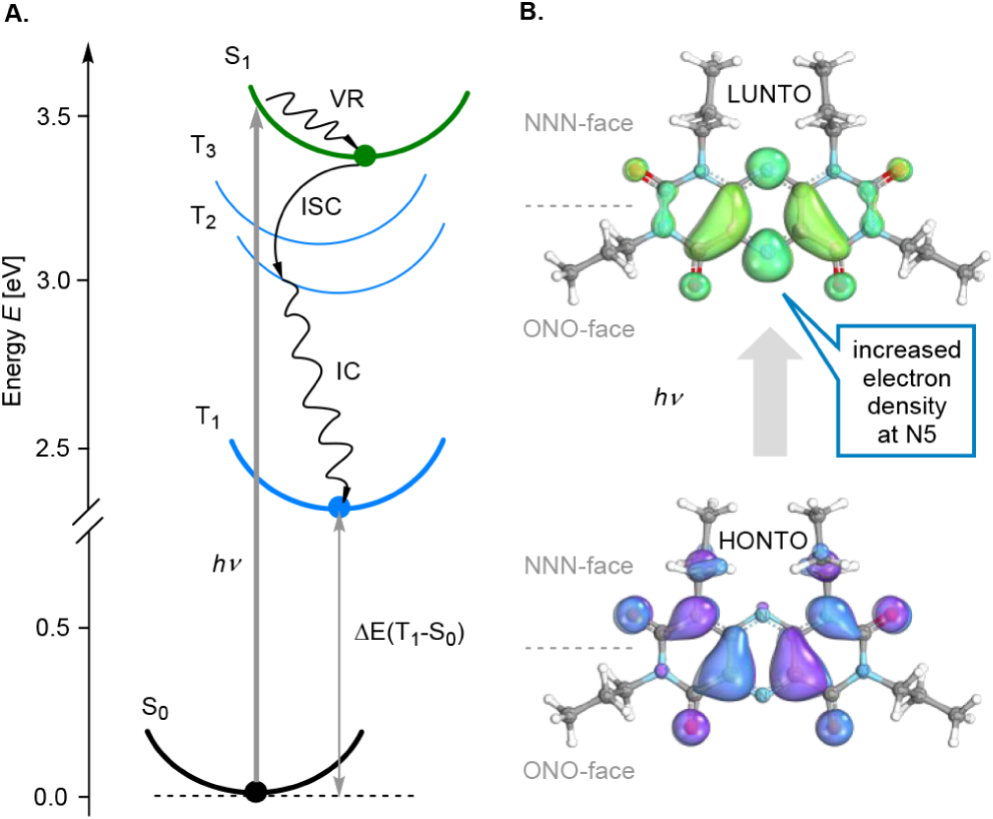
A. Jablonski diagram of **PrPPT** with calculated energy levels and proposed initial excited states evolution. B. Natural Transition Orbitals for the S_1_ state (identical for T_1_, see SI) with a distinct intramolecular charge transfer to the electron-deficient *ONO* face, increasing electron density at N5.

We next evaluated the spatial electronic distribution of the unpaired spin density in **PPTH** ^•^ (Figure 6). Natural Bond Orbital (NBO) analysis revealed predominant radical localization at the hydrogen-bound N5 nitrogen atom, with additional spin delocalization across the central pyrazine core, particularly at N10, as well as minor contributions at C4a and C5a (Figure 6A). This electronic distribution is consistent with the formation of a two-center, three-electron (2c–3e) N–H bond (Figure 5B). Such bonding motifs are well-established in redox-active pyridinium salts following single-electron reduction and are known to play a key role in radical group transfer processes.^63,64^ In this context, out-of-plane bending of the exocyclic bond and the associated decrease in bond order, attributed to π*→σ* interactions, are critical features. In the present system, a successive out-of-plane bending (around 30.7°) coincides with the elongation of the N–H bond by 0.84 Å in both the absence and presence of an external acceptor (Figure 6B).

**Figure 6:**
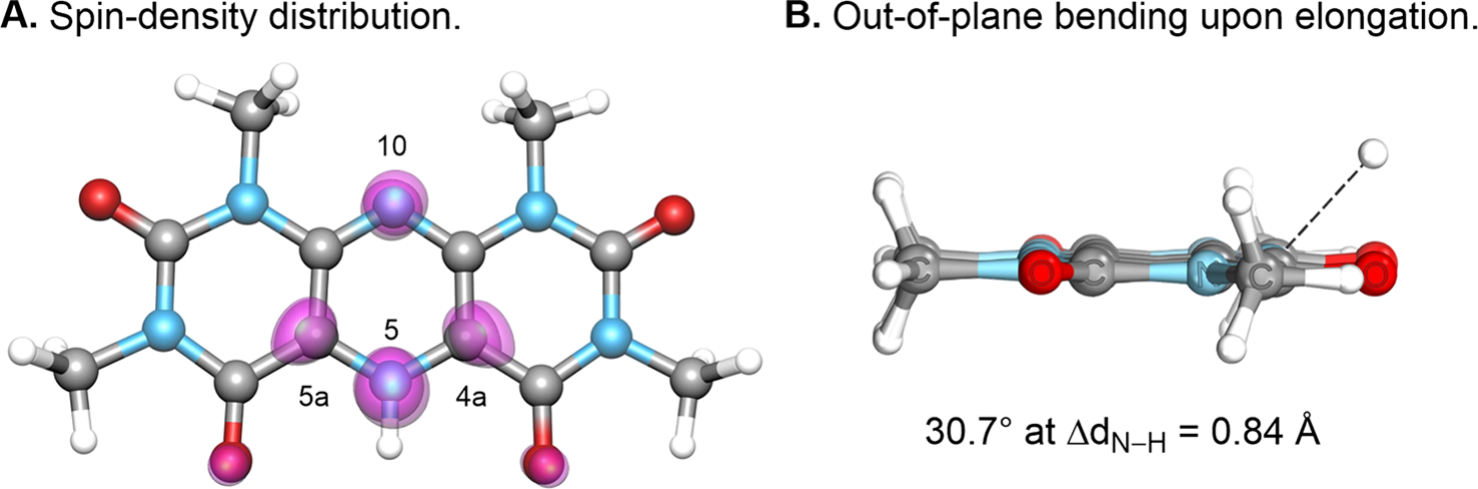
A. Spin density distribution of the **PPTH^•^** computed at the CAM-B3LYP­(D3BJ)/def2-TZVP in MeCN, which shows localization at N5 with delocalization across the pyrazine core, consistent with a 2c–3e N–H bond. B. Out-of-plane N–H bond distortion upon elongation.

Pyrimidopteridinetetraones have emerged as powerful organic photoredox catalysts, enabling decarboxylative C–C,^45^ C–O,^47^ and C–N^48^ bond formations, as well as deuterium labeling,^46^ under mild and additive-free conditions. **PPT** photoredox catalysis offers the unique opportunity to study a photomediated catalytic transformation solely operating via a single catalyst and a purely PCET-based reaction manifold. In contrast, in most bifunctional PCET photoredox systems, the initially formed radical is rapidly intercepted by electron-deficient alkenes via a SET pathway, rendering a direct turnover of the protonated catalyst ambiguous.^24,34^ This study addresses this knowledge gap by systematically probing the reactivity of **PPTH** ^•^ and its role in driving catalyst turnover beyond conventional SET scenarios. We therefore sought to investigate the mechanistic trajectory of the formal hydrogen-atom transfer from the **PPTH** ^•^ species under conditions where an SET pathway is thermodynamically inaccessible $\left(\Delta G_{SET}>0eV\right)$ .

We focused on the PPT-catalyzed protodecarboxylation of carboxylic acids, a transformation previously reported by our group,^46^ allowing us to capitalize on the mechanistic simplicity and relevance of HAT or PCET to catalytic turnover as well as its synthetic relevance (Figure 1D and Scheme 1, below).^65–68^ The generation of **PPTH** ^•^ from carboxylic acids, as supported by the findings *vide supra*, is postulated to involve a homolytic O–H bond cleavage, followed by a decarboxylation to generate a neutral carbon-centered radical (Figure 4). While most synthetically useful carboxylic acids fall within a narrow O–H BDFE range,^69^ their p*K*_a_ values^70^ and oxidation potentials^71^ vary significantly depending on the reaction environment, meaning that in some cases consecutive ET–PT pathways could also contribute to **PPTH** ^•^ formation. Moreover, the fate of the resulting carbon-centered radicals upon decarboxylation is determined by their intrinsic redox potentials and the thermodynamics of subsequent product-forming steps (Table 1). Understanding these processes is therefore critical to defining the trajectory of **PPTH** ^•^-mediated radical turnover and, more broadly, the full potential of PPT as well as bifunctional photoredox catalysis.

**Scheme 1:**
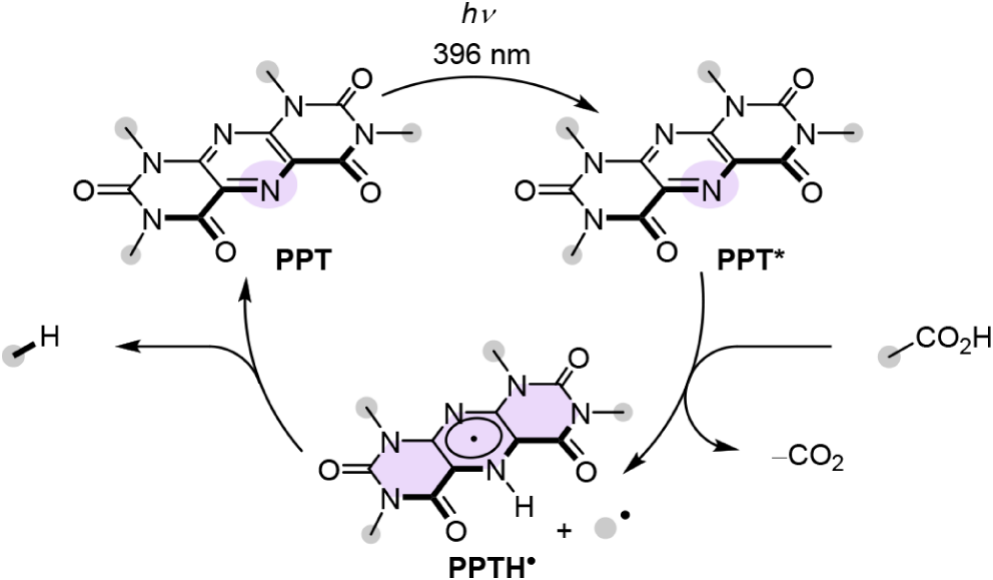
General Scheme for the **PPT**-Catalysed, Base-Free Protodecarboxylation of Carboxylic Acids

**Table 1:**
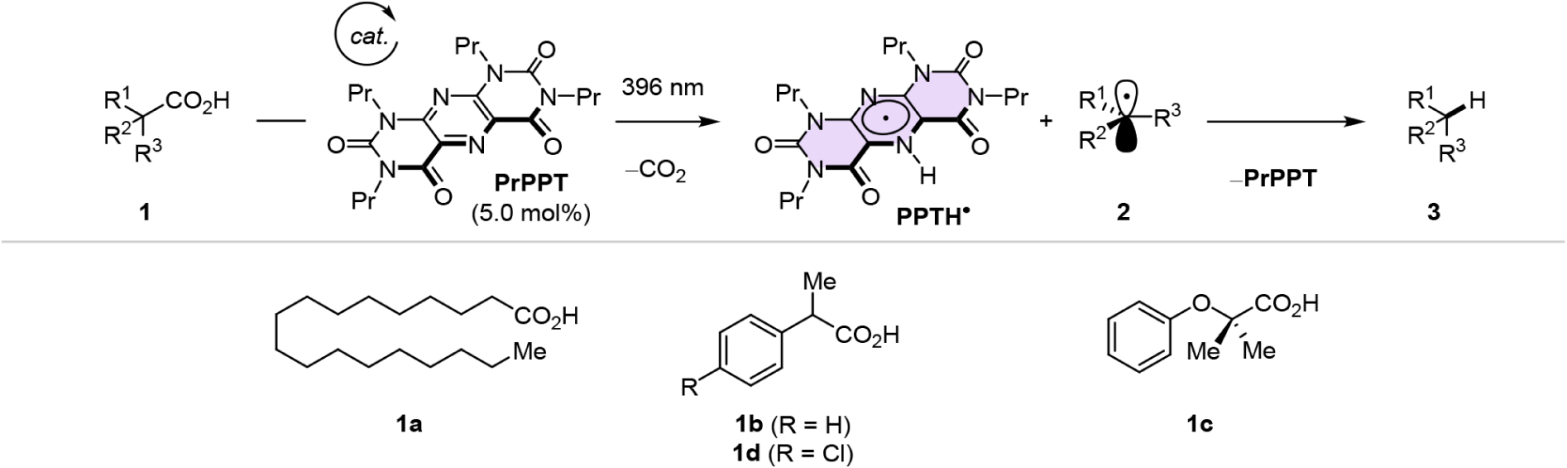
Thermodynamic Parameters of Carboxylic Acids and Carbon-Centered Radicals

			*E*_ox_ ^b^ [eV]		Δ*G*°_ET_ ^c^ [kcal mol^–1^]			*E*_red_^b^ [eV]		
Entry	Compound	p*K*_a_ ^a^ MeCN	RCO_2_H	RCO_2_^–^	RCO_2_H	RCO_2_^–^	ΔBDFE^*^ ^d^ [kcal mol^–1^]	R**^•^**	Δ*G*°_ET_ ^e^ [kcal mol^–1^]	ΔBDFE^f^ [kcal mol^–1^]
1	1a	21.2	>+3.0	>+3.0	+43	+43	–2	–2.0	+47	–34
2	1b	21.1	+2.40	+1.29	+29	+3	–3	–1.34 ^68,69^	+9	–22
3	1c	18.8	+2.08	+1.26	+21	+3	–3	+0.15 ^68^,^f^	–3	–28

a In MeCN. Calculated from p*K*_a_ values in water using p*K*_a_(MeCN) = (p*K*_a_(H_2_O) + X +CO · 2.2 – nC · 0.13 – MW · 0.0017 + 6.5)/0.55.^55^

b Referenced to NHE by adding +0.63 V to the value measured against Fc^+^/Fc.

c From Δ*G*° = (*E*_ox, Sub_ – ^3^ *E*_red, PPT_*) · 23.06 in kcal mol^–1^.

d ΔBDFE = BDFE_O–H_ – BDFE*_PPTH•_ in kcal mol^–1^. BDFEs were calculated using the ALFABET BDE predictor.^69^

e From Δ*G*° = (*E*_red, **PPT**_ – *E*_red, Sub_) · 23.06 in kcal mol^–1^.

f ΔBDFE = BDFE_**PPTH**•_ – BDFE_C–H_ in kcal mol^–1^. From BDFE values for C–H bonds.^69^

Both **PPTH** ^**•**^ and the substrate radical (**sub** ^**•**^) exist as independent, uncorrelated doublets. When these radicals encounter each other in solution, their spins combine to form either a singlet or triplet radical pair, with a statistical ratio of 1:3 (singlet:triplet). Consequently, singlet encounters are formally spin-allowed and can react immediately; they are less frequent. Triplet radical pairs, although initially spin-forbidden for direct bond formation, generally have longer lifetimes in solution, allowing diffusion, reorientation, and intersystem crossing to productive states. As a result, both singlet-like and triplet-like configurations may contribute to the reaction.

We computationally investigated the hydrogen transfer mechanisms from the **PPTH** ^**•**^ radical across a series of substrates representing distinct radical stabilities and electronic properties: (i) *n*-butyl radical **A**, an unstabilized primary aliphatic radical; (ii) ethylbenzyl radical **B**, a secondary stabilized benzylic radical; and (iii) isopropoxybenzene radical **C**, a redox-active, noninnocent radical with partial oxygen-centered character. We first conducted a comprehensive reaction-pathway energy analysis for the encounters between **PPTH** ^**•**^ and **sub** ^**•**^ with antiparallel and parallel spin alignment, described computationally using broken-symmetry singlet (BS) and triplet reference states, respectively (Figure 7). The BS singlet encounter, characterized by ⟨S^2^⟩ values close to 0, is statistically less favored but proceeds via a markedly lower activation barrier than the triplet pathway (ΔΔ*G* ^‡^ = >25 kcal mol^–1^). As the antiparallel and parallel spin encounters proceed via distinct transition states, the reaction outcome is controlled by the difference in activation free energies rather than by their statistical populations, thereby establishing the singlet pathway as kinetically dominant.

**Figure 7:**
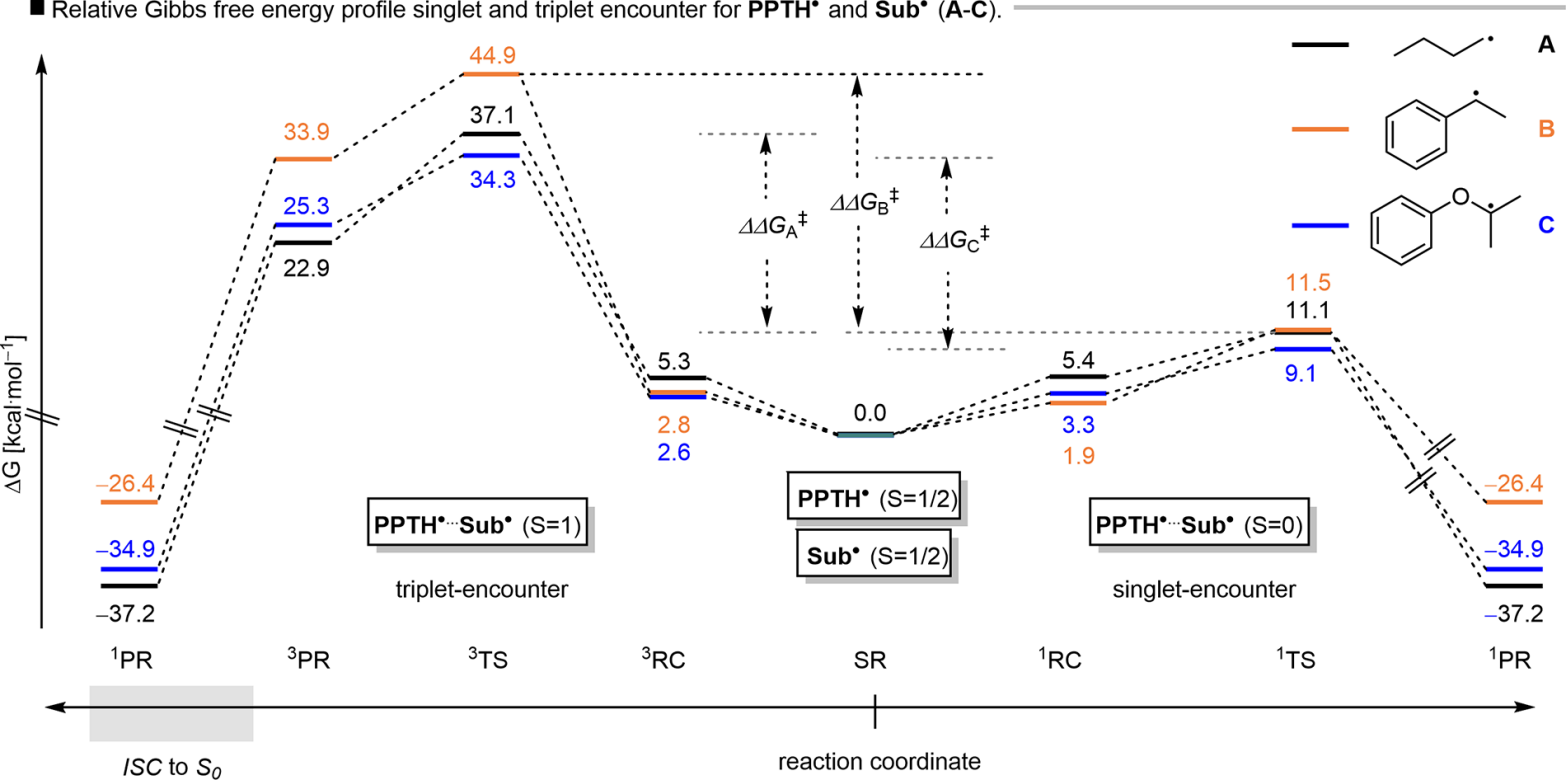
Calculated Gibbs free-energy profiles (Δ*G*) for the radical–radical coupling of **PPTH^•^** with (A) *n*-butyl, (B) benzyl, and (C) phenoxy radicals. The profiles compare the energetics of the spin-projected broken-symmetry singlet (^1^BS) and triplet (^3^T) pathways. ΔΔ*G* ^‡^ represents the difference in reaction barriers between the singlet and triplet encounters.

To directly visualize the distinct electron-flow patterns underlying the reaction pathways, we employed intrinsic bond orbital (IBO) analysis.^51,72^ The application of IBOs along the intrinsic reaction coordinate (IRC) to distinguish between HAT and cPCET mechanisms was pioneered by Knizia and coworkers,^73^ who showed that direct inspection of electronic wave functions can provide intuitive mechanistic insights that are often inaccessible through conventional experimental or theoretical approaches. By tracking the evolution of α- and β-spin IBOs in both donor and acceptor species, we identified characteristic electronic signatures that clearly distinguish dHAT from cPCET and stepwise ET-PT mechanism, and revealed how the spin state of the encounter, substrate electronics, and geometry influence the preferred pathway. Figure 8 illustrates the electron flow of the most indicative IBOs during the hydrogen transfer reaction between **PPTH** ^•^ and three representative radical substrates. The whole set of strongly changed IBOs for all pathways is collected in the Supporting Information.

**Figure 8:**
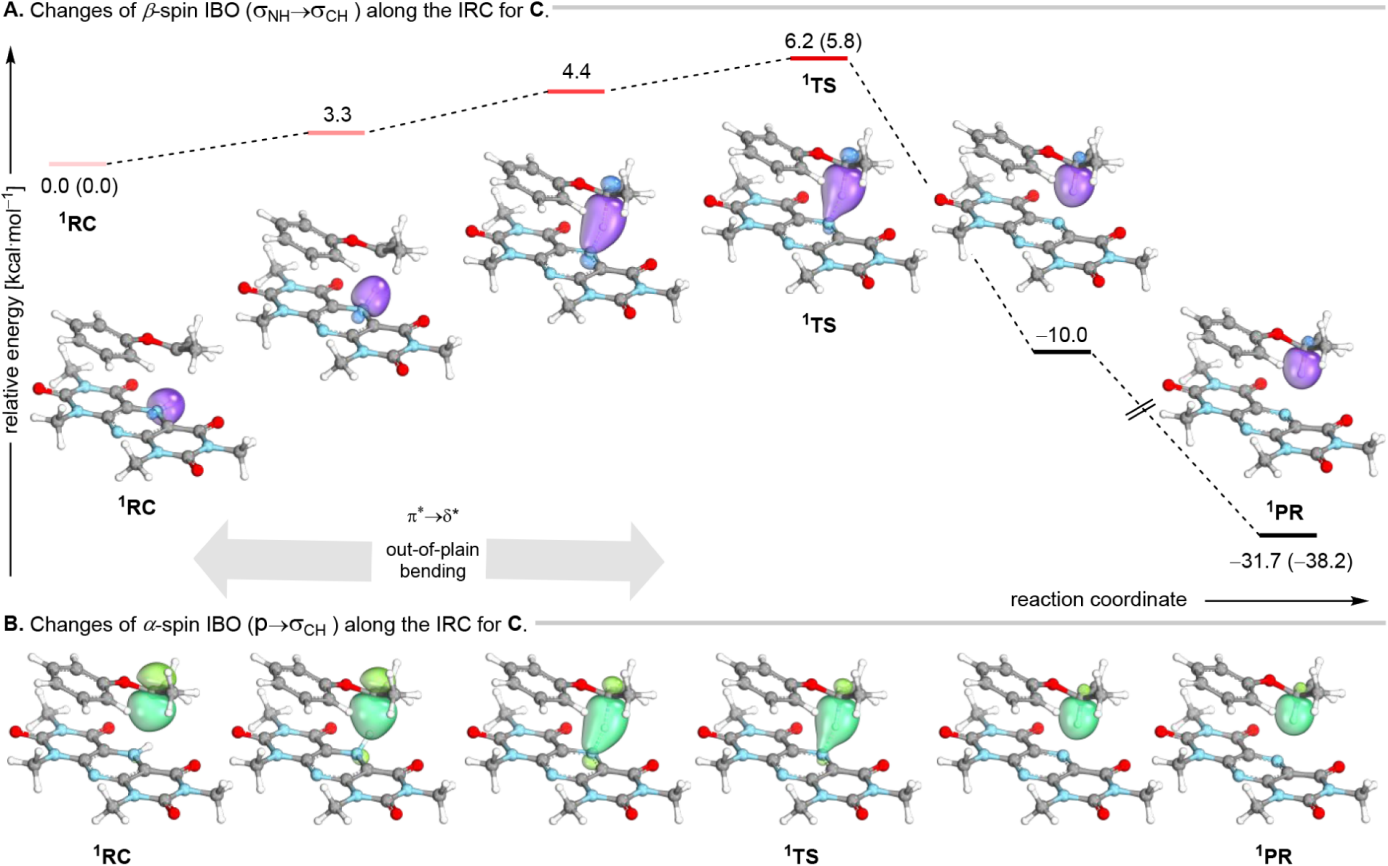
IBO analysis and relative energy profiles, including free energies of stationary points in parentheses along the reaction coordinate of the singlet encounter between **PPTH^•^** and substrate radical **C**. The intensity of the reddish horizontal lines from the reactant complex to the transition state indicates the extent of out-of-plane bending, which corresponds to the π* → σ* interaction.

For the singlet encounter, the reaction proceeds uniformly via a dHAT pathway for all substrates **A**–**C**. Accordingly, the transition state features a concerted transfer of the hydrogen atom and its associated electron, with activation Gibbs free energies ranging from 5.7 to 9.6 kcal mol^–1^ from reactant complex ^1^RC. The reaction pathway identified from this analysis is shown in Figure 8. The phenoxypropyl radical **C** was selected as a representative example because its electronic structure renders it particularly susceptible to alternative PCET pathways while nevertheless displaying a clear preference for the dHAT mechanism. Specifically, the α-electron from the σ­(N–H) bonding orbital is transferred into the semioccupied p-orbital of the substrate’s carbon-centered radical, where it pairs with the β-spin electron to form the new C–H bond. Concurrently, the α-spin electron initially residing in the σ­(N–H) orbital remains localized on the **PPT**, contributing to the N5 lone pair (see SI).

In contrast, the triplet encounter, defined by parallel alignment of the unpaired electrons, exhibits a substrate-gated switch between distinct reaction pathways such that product formation depends sensitively on the electronic nature of the substituents. Specifically, the mechanistic trajectories range from dHAT for unstabilized radicals (**A**), to cPCET for stabilized benzylic radicals (**B**), and to a stepwise ET–PT–ET sequence for the conversion of the redox-active, stabilized phenoxypropyl radical (**C**). These pathways are associated with substantially higher activation free energies, reaching up to ∼42 kcal mol^–1^, and are illustrated in the Supporting Information.

Guided by the mechanistic insights obtained through IBO analysis, we reevaluated the thermodynamic parameters governing the formation and reactivity of the carbon-centered radicals listed in Table 1. These include key parameters such as BDFEs, reduction potentials, and free energy changes associated with HAT (Δ*G*_HAT_) and SET (Δ*G*_ET_). As shown in Table 1, entry 1, the generation of the aliphatic primary carbon-centered radical from stearic acid **1a** ought to proceed through dHAT or cPCET and is expected to be independent of external base activation. The subsequent reduction to the corresponding carbanion is thermodynamically prohibitive (Δ*G*_ET_ = +20 kcal mol^–1^). However, the dHAT pathway is strongly exergonic with Δ*G*_HAT_ = −34 kcal mol^–1^. Most test reactions were performed at 50 °C to improve substrate solubility and accelerate the decarboxylation step. This modest temperature increase has a minimal impact on redox potentials, BDFEs, or p*K*_a_ values and does not affect the mechanistic conclusions or catalytic performance. We experimentally confirmed that the photomediated protodecarboxylation of stearic acid **1a** by **PrPPT** proceeds in the absence of added base, with only a modest decrease in yield from 18% to 10%, however, remaining in an overall low-yielding regime (Figure 9). In contrast, other substrates showed either substantial depletion by 56% for **1d** or no conversion in the case of **1c**. In our previous study, protodecarboxylation of benzylic carboxylic acids was found to require basic conditions to achieve high yields.^46^ These observations suggest that a stepwise PT–ET mechanism may operate in the initial sequence. The benzylic radical formed from **1b** exhibits an endergonic reduction (Δ*G*_ET_ = +9 kcal mol^–1^)^74^ but a favorable ΔBDFE (Δ*G*_HAT_ = −22 kcal mol^–1^), suggesting a plausible ET–PT–ET PCET mechanism despite the slightly uphill electron transfer step. Its corresponding carbanion intermediate is highly basic (p*K*_a_ = 51.1 in MeCN),^75^ rendering a protonation strongly favorable. In fact, a 1,2-addition adduct of the corresponding benzylic carbanion derived from **1d** was identified by HPLC-MS and HRMS, supporting the involvement of a carbanionic intermediate in line with the proposed mechanism (Scheme 2).^76,77^ The phenoxypropyl radical derived from **1c** is thermodynamically even more competent to engage in an initial electron transfer event (Δ*G*_ET_ = −3 kcal mol^–1^). However, the corresponding carbanion could not be trapped, presumably due to its steric demand and instability and generally poor reactivity under base-free conditions.

**Figure 9:**
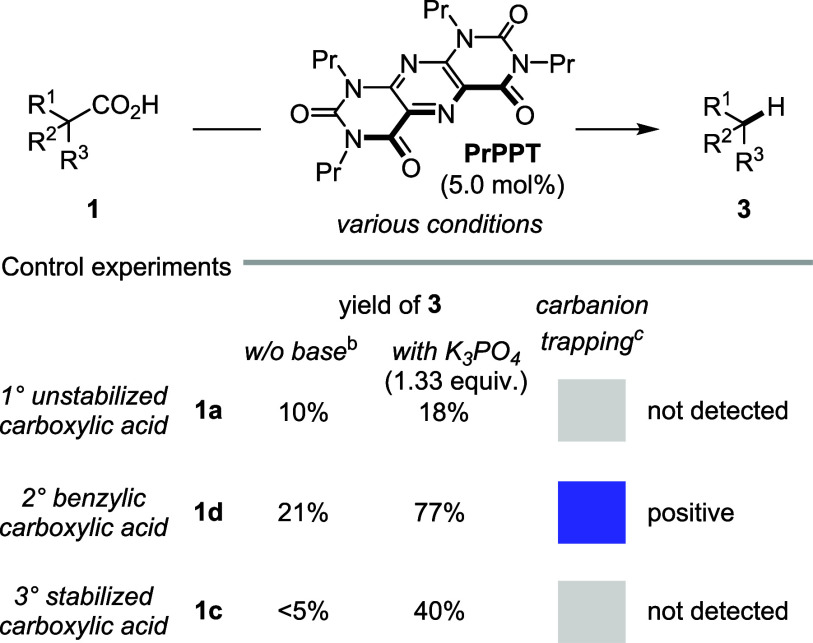
Protodecarboxylation yield of acid **1a**–**1d** in the presence and absence of added base. ^a^Detailed reaction conditions are given in the ESI. ^b^Yields were determined by calibrated GC using biphenyl as an internal standard. Deviation in the yield of the protodecarboxylation to yields according to ref.^45^. ^c^The reaction was performed in the presence of acetone (3.0 equiv).

**Scheme 2:**
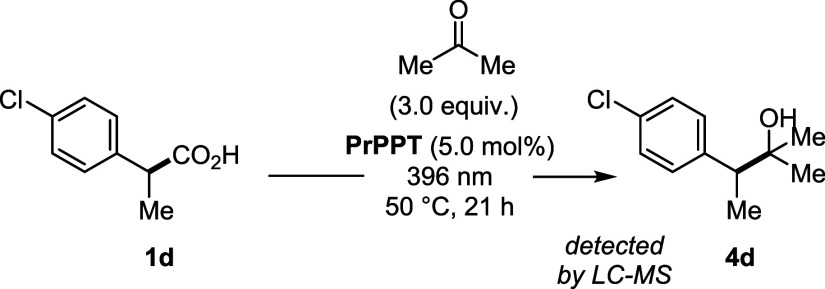
Trapping Experiment of Intermediary Carboanions Formed upon Decarboxylation and Reduction of the Benzylic Radical with Acetone

Taken together, these thermodynamic parameters illustrate that the interplay between substrate acidity, oxidation potential, and bond dissociation free energy dictates the driving force for hydrogen atom transfer, providing a clear mechanistic rationale for the observed PCET/dHAT reactivity trends.

In summary, these data underscore how subtle differences in substrate electronics and radical stability dictate the operative PCET mechanism. While alkyl radicals favor direct HAT pathways, benzylic and heteroatom-stabilized radicals permit stepwise sequences involving carbanion intermediates. Collectively, these results highlight the value of intrinsic bond orbital (IBO) analysis in disentangling complex PCET mechanisms and how substrate electronics and structural features govern the preferred hydrogen transfer pathway.

Looking ahead, the distinct PCET reactivity of **PPTH** ^**•**^ suggests clear opportunities for extending this mechanistic framework to new bond-forming chemistry. The ability of stabilized radicals to engage in stepwise ET–PT–ET sequences points toward applications in selective C–C and C–heteroatom coupling, while the strong H-atom acceptor capability of the excited states could enable mild C–H activation and progress hydrofunctionalization processes without external HAT reagents. Although exploratory, these directions illustrate how the thermodynamic and electronic principles mapped here can guide the development of next-generation PPT-mediated photocatalytic transformations.

## Conclusion

In summary, this study establishes a comprehensive thermodynamic and mechanistic framework for the formation and reactivity of the N5-hydropyrimidopteridinetetraone radical derived from pyrimidopteridinetetraone-based photocatalysts. Through a combination of spectroscopy, electrochemistry, and (TD)­DFT calculations, we map the excited-state energetics, ground- and excited-state p*K*_a_ values, and bond dissociation free energies that underpin hydrogen atom transfer reactivity in this system. These data show that the excited-state PPT* combines favorable photophysical properties with strong electron- and proton-accepting ability, enabling efficient generation of the **PPTH** ^**•**^ radical under mild conditions. Spin-resolved mechanistic analysis reveals that **PPTH** ^**•**^ undergoes catalyst turnover predominantly through a direct hydrogen atom transfer pathway in singlet radical encounters, irrespective of the substrate class. This uniform, low-barrier mechanism enables facile N–H bond scission and regeneration of the parent PPT catalyst. In contrast, triplet radical encounters access substantially higher-energy reaction manifolds and may follow distinct mechanistic trajectories, including dHAT, concerted proton-coupled electron transfer, or stepwise electron- and proton-transfer sequences, depending on substrate stabilization. Intrinsic bond orbital analysis along the intrinsic reaction coordinate delineates the electronic features associated with these spin-dependent pathways and clarifies the mechanistic boundaries between them. Collectively, these findings highlight spin state and thermodynamic control, rather than substrate gating alone, as key determinants of the hydrogen transfer reactivity in **PPT** photoredox catalysis. The results position **PPT**-based photocatalysts as powerful and self-sufficient platforms for sustainable photoredox transformations that obviate the need for sacrificial HAT donors or cocatalysts, while providing general design principles for next-generation proton- and electron-coupled catalytic systems.

## Computational Details

All ground- and excited-state calculations were performed using density functional theory (DFT) and time-dependent DFT (TDDFT) as implemented in the ORCA program package (version 5.0.3).^78^ The CAM-B3LYP functional^79,80^ in combination with the def2-TZVP basis set^81^ was employed, with dispersion effects accounted for using the D3 correction with Becke–Johnson damping.^82^ Solvent effects (acetonitrile) were included using the conductor-like polarizable continuum model (CPCM).^83,84^ Stationary points were optimized and characterized by vibrational frequency analysis. Transition states were confirmed by a single imaginary frequency and connected to minima by intrinsic reaction coordinate (IRC) calculations.^85,86^ Thermal and entropic corrections to Gibbs free energies were applied in the temperature range 298.15–323.15 K. Excited-state and charge-transfer properties were analyzed using NBO,^51,87^ IBO,^51,72,88^ and TheoDORE analyses.^61^ Simulated absorption spectra were obtained by the Gaussian broadening of calculated stick spectra. Details of spectroscopic measurements, thermochemical data, and additional computational procedures are provided in the Supporting Information. Optimized Cartesian coordinates of all species are included in a separate Excel file.

## Supplementary Material





## Data Availability

The data underlying this study are available in the published article and its Supporting Information.

## Notes

General experimental considerations; detailed experimental procedures; spectroscopic characterization data; computational details (PDF)
Optimized geometries in XYZ format (XLSX)
