# Supplementary material for: Driving Forces and Spin-Gated Reactivity of N5-Hydropyrimidopteridinetetraone Radicals in Photocatalysis
Source: J Org Chem. 2026 Apr 15;91(16):5520–32. doi: 10.1021/acs.joc.6c00017 (PMC13122643; doi:10.1021/acs.joc.6c00017)
Supplement: Supplementary file 1 [file jo6c00017_si_001.pdf]

Electronic Supporting Information

Driving Forces and Spin-Gated Reactivity of N5-Hydropyrimidopteridinetetraone Radicals in Photocatalysis

Zohreh Amanollahi[a], Luisa Zach[a], Tobias Taeufer[a], Thanh Huyen Vuong[a], Olga S. Bokareva*[a,b,c] Jola Pospech*[a,b]

[a]Leibniz Institute for Catalysis (LIKAT), Albert-Einstein-Str. 29a, 18059 Rostock, Germany.

[b]Department of Life, Light & Matter, University of Rostock, Albert-Einstein-Str. 21, 18059 Rostock, Germany.

[c]Institute of Chemistry, University of Rostock, Albert-Einstein-Str. 27, 18059 Rostock, Germany.

Table Sof Contents

1. General remarks S3
2. Excited-state characteristics S5
2.1. Experimental contribution S5
2.2. Computational analysis S6
3. Fragmentation analysis for PPT compounds S9
4. ΔG of the PPTH$^{\bullet}$ S10
4.1. Protonation on ONO vs. NNN-face S10
5. Determination of redox potentials S11
6. Determination of pK_a values S14
6.2. Computational estimation of the pK_a values S18
7. Determination of pK_a* S20
8. Bond dissociation free energies of the exocyclic PPTH$^{\bullet}$ N-H bond S21
8.1. Square Scheme Sanalysis S21
8.2. Computational determination of the BDFE of PPTH$^{\bullet}$ S24
9. EPR spectroscopy S25
10. Analysis of reaction pathways S26

10.2. IBO analysis S28

10.2.1. Singlet pathway S28

10.2.2. Triplet pathway S32

11. Experimental validation of IBO analysis S37

12.1. Photo-mediated protodecarboxylation of carboxlic acids 1a-1d S38

11.2. Capture of carbanion intermediates S42

12. Abbreviations S44

13. References S45

1. General remarks

All chemicals were purchased from commercial suppliers and used as received. Pyrimidopteridinetetraones (PPT) were synthesized according to procedures previously reported by our group.[1] Dry acetonitrile (MeCN) was obtained by drying commercially available anhydrous, oxygen-free HPLC-grade solvents through activated molecular sieves. The UV-Vis spectra were recorded with a spectrophotometer UV5 from METTLER TOLEDO. The fluorescence measurements were conducted with a Cary Eclipse Fluorescence Spectrometer from AGILENT. All electrochemical investigations were performed at room temperature in hplc grade acetonitrile p.A. (VWR) under an Argon atmosphere with 0.1 M tetrabutylammonium hexafluorophosphate (FLUKA) as conducting salt using an Autolab (PGSTAT 204, METROHM). Prior to electrochemical measurements, the solutions were degassed by bubbling argon for 15 min and maintained under an inert atmosphere during the experiment. A glassy carbon disk electrode (d = 2 mm) was used as the working electrode, a plartinum sheet electrode (6x8 mm) as the counter electrode, and an Ag/AgCl/LiCl sat. in EtOH-system as the reference electrode (all electrodes from METROHM). Starting point of all measurements is the OCP after 60s. All potentials mentioned in this paper were measured with respect to this reference system and were checked by using the ferrocenium/ferrocene-internal reference system. Voltammograms are plotted according to the IUPAC convention with anodic currents shown as positive.

Ground- and excited-state calculations were performed within the framework of density functional theory (DFT) and its linear-response extension, TDDFT. To achieve an accurate description of charge-transfer properties crucial for photocatalysis, the range-separated hybrid functional[2, 3] CAM-B3LYP together with the triple-ζ quality def2-TZVP basis set[4] and the D3 dispersion correction with Becke–Johnson damping (D3BJ)[5] was applied. Solvent effects were included using the conductor-like polarizable continuum model (CPCM)[6, 7] with acetonitrile as the solvent. Transition states were located via relaxed surface scans along the relevant reaction coordinates and further optimized to stationary points. Vibrational frequency calculations were carried out at the same level of theory to characterize stationary points: a single imaginary frequency confirmed transition states, while its absence indicated true minima. Intrinsic reaction coordinate (IRC) calculations[8, 9] were performed to verify the proper connectivity between reactants, transition states, and products with default convergence criteria (TolMAXG = 2.0×10^{-3}, TolRMSG = 5.0×10^{-4}). Along these IRCs, electronic structures were recomputed with energies of each point along the trajectory converged to the *VeryTightSCF* threshold. Furthermore, IRC calculations were used to unravel the mechanism of the HAT step and to monitor the electron flow via the intrinsic bond orbitals (IBOs)[10, 11] generated using IboView (iboexp = 2).[12] Thermal and entropic corrections to the Gibbs free energy were applied from 298.15 K to 323.15 K in 5 K increments to account for temperature-dependent effects.

Moreover, because the optimization of excited states may suffer from root reordering, the identities of the optimized S_1, T_1 and T_2 states were verified by analyzing the dominant orbital transitions at the initial

and optimized geometries rather than relying on state numbering alone. RMSD values relative to the ground-state geometry and excitation energies before and after optimization are provided in the Table SS3.

Additionally, natural bond orbital (NBO) analysis[10, 13] was employed to assess charge and spin distributions across the species involved in various mechanistic steps. The broadening of the resulting absorption stick spectra was done with Gaussians of FWHM 0.20 eV. Excited-state analysis was performed using the TheoDORE package,[14] which enables the automatic quantitative analysis of wavefunctions and localization of excitations at predefined molecular moieties. The excitation-based fragmentation analysis (hierarchical clustering) was performed according to the strategy suggested in the literature.[15]All calculations were performed using the ORCA program package, version 5.0.3.[16] Pre- and post-processing of data was done with homemade programs. The optimized structures of all species involved in this study in XYZ format are tabulated in a separate Excel file.

2. Excited-state characteristics

2.1. Experimental contribution

The singlet excited-state energy ($^1E_{0,0}$) of **PPT** and its protonated form **PPTH**$^+$ was experimentally determined from the intersection of the normalized absorption and emission spectra (see **Fehler! Verweisquelle konnte nicht gefunden werden.**). UV–vis absorption was recorded at a concentration of [96 µM] in MeCN, and the emission spectrum was obtained under identical conditions upon excitation at [340 nm]. Measurements of the protonated form **PPTH**$^+$ were carried out at pH 2.45 in MeCN. Both spectra were baseline-corrected and normalized to unity.

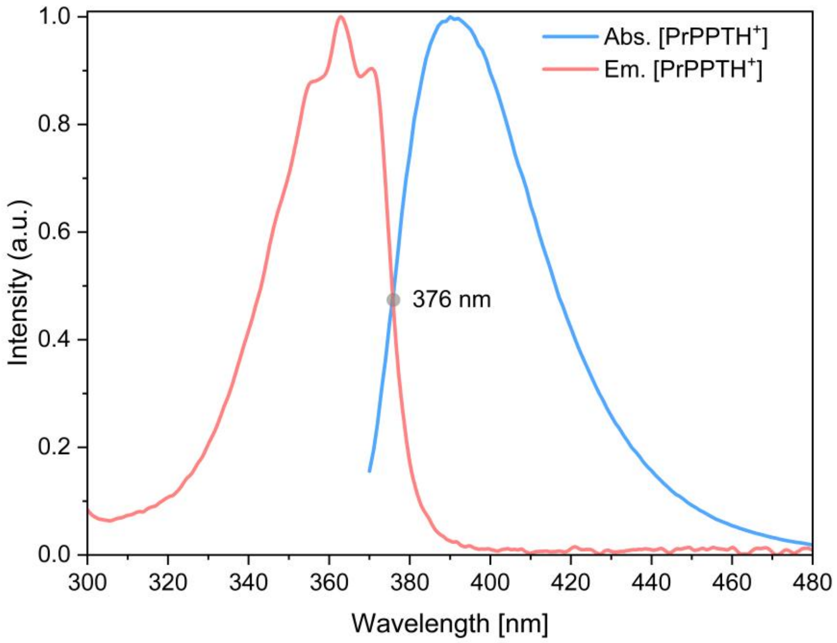

Figure SS1:Absorption and emission from ***PrPPT*** *in MeCN at pH 2.45. Acidified with TfOH.*

The S_0-S_1 transition energy ($^1E_{0,0}$) was extracted from the wavelength of spectral overlap at 375 nm for **PPT**[1] and 376 nm for **PPTH**$^+$, corresponding to the energy of the lowest vibrational level of the excited state. The excited-state energy was calculated using Equation:

$$^1E_{0,0} = \frac{hc}{\lambda_{0,0}}$$

Accordingly, the excited-state energies were:

$$^1E_{0,0}(PPT) = 3.31\ eV$$

$$^1E_{0,0}(PPTH^+) = 3.30\ eV$$

2.2. Computational analysis

To analyze the excited states properties of **PPT**, the 100 lowest vertical singlet and triplet excitations were computed. As all relevant processes for photocatalysis occur in the lowest excited states, the following discussion focuses on the low-energy UV region (250-400 nm) in detail (Figure SS1). Different theoretical models were employed to investigate the impact of various effects on the shape of absorption spectra. In total, the low energy part is dominated by the absorption to the lowest singlet state S_1, which is of clear HOMO→ LUMO character illustrated by NTOs in Figure SS2). The inclusion of the SOCs effects introduces only minor changes in the total shape of the absorption spectrum (blue solid and blue dashed lines overlapping).

Furthermore, to understand the origin of the offset between theoretical and experimental absorption maxima, the vibrational effects were taken into account by averaging vertical spectra computed on a set of initial geometries from the Wigner distribution based on displacement along harmonic vibrations. The two sets of coordinates (20 and 100 conditions) yielded similar absorption spectra, indicating that further increasing the calculation setup is not expected to improve the results. As the maxima of Wigner-based spectra are closer to the experimental maximum than the original pure vertical calculated spectra, we conclude that the shift between the measured and computed spectra can be attributed to the vibrational effects beyond the harmonic approximation. As benchmarking different flavors of TDDFT to reach the best match for S_1 excitation is beyond the scope of the current publication, we introduce a correction factor of 0.72 eV to account for this offset and to maintain a consistent quantum-chemical approach throughout the study.

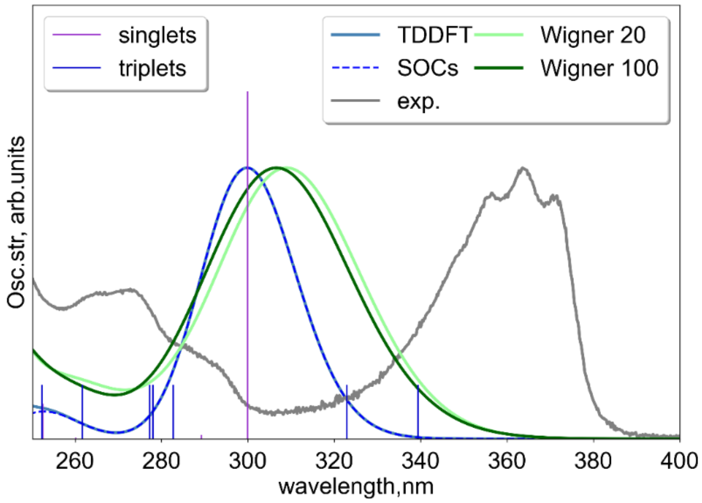

Figure SS1: Experimental and theoretical absorption spectra calculated with CAM-B3LYP with different models

Calculated excitation energies (TDDFT) were systematically corrected by applying a uniform energy shift to match the experimentally observed absorption maxima. This empirical correction ensures better alignment between computed and experimental spectra. The correction assumes a constant offset across transitions.

*Table SS1: Calculated vertical excitation energies, spin–orbit coupling (SOC) matrix elements, and corrected excitation energies obtained from TDDFT at the CAM-B3LYP/def2-TZVP level, compared with the experimental value for **PPT**. Adiabatic S_0-S_1 and S_0-T_1 energies are given in parentheses.*

**PPT**	E [eV]	SOC [cm^{-1}]	corr. calc. E [eV]	exp. E [eV]
S_1	4.13 (3.99)		3.31	3.31
T_1	3.01 (2.79)	0.51	2.29	
T_2	3.65	2.73	2.93	
T_3	3.84	0.43	3.12	

Table SS2: Calculated vertical excitation energies, spin–orbit coupling (SOC) matrix elements, and corrected excitation energies obtained from TDDFT at the CAM-B3LYP/def2-TZVP level, compared with the experimental value for $PPTH^+$. Adiabatic S_0-S_1 and S_0-T_1 energies are given in parentheses.

**$PPTH^+$**	E [eV]	SOC [cm^{-1}]	corr. calc. E [eV]	exp. E [eV]
S_1	3.68 (3.37)		3.30	3.30
T_1	2.22 (1.95)		1.84	
T_2	3.51		3.13	
T_3	3.64		3.26	

Table SS3: Calculated vertical excitation energies from TDDFT at the CAM-B3LYP/def2-TZVP level for PPT geometries optimized in the S_0, S_1, and T_1 electronic states.

PPT

Geomtery	S_1 [eV]	T_1 [eV]	T_2 [eV]
S_0-OPT	4.13	3.01	3.84
S_1-OPT	3.85	2.70	3.74
T_1-OPT	3.81	2.62	3.63

*Table SS4: Calculated vertical excitation energies from TDDFT at the CAM-B3LYP/def2-TZVP level for **$PPTH^+$** geometries optimized in the S_0, S_1, and T_1 electronic states.*

$PPTH^+$

Geomtery	S_1 [eV]	T_1 [eV]	T_2 [eV]
S_0-OPT	3.68	2.21	3.51
S_1-OPT	3.38	1.91	3.17
T_1-OPT	3.38	1.80	3.22

Considering a correction factor for further analysis, the lowest vertical excited states in the singlet and triplet manifolds are further analyzed by Jablonski diagrams and Natural Transition Orbitals, which represent the character of the involved states. As the S_1 state is the brightest in the low-energy UV regions, it should be initially excited. Subsequently, the population is transferred to the lower-lying triplet manifold due to spin-orbit coupling. The most significant SOC elements are between S_1 and T_2 states. As is usually the case, the lowest triplet state is involved in photochemical reactions; therefore, the population should decay further to the T_1 state, where it stays long enough to allow for further processes. S_1, T_1, and T_2 share the same destination NTO (LUMO orbital), while the initial NTOs are

all localized on the central PPT core, with significant spatial overlap between them, which in turn can facilitate decay to the lowest triplet state.

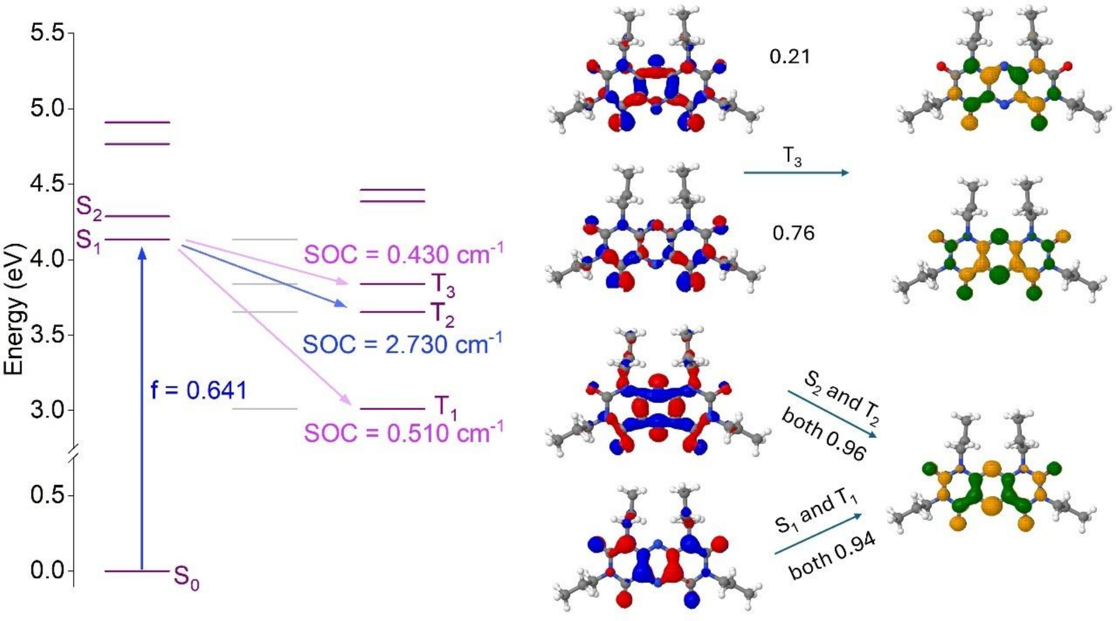

Figure SS2: Jablonski diagram of the lowest excited states of PPT as computed with CAM-B3LYP/def2-TZVP. SOCs between the lowest singlet S_1 and low-lying T_1-T_3 are also marked. (left) Natural Transition Orbitals of the most important excited states, together with NTOs contributions (right).

*Table SS5: Calculated vertical and adiabatic excitation energies from TDDFT at the CAM-B3LYP/def2-TZVP level for **PrPPT** and **MePPT**, using geometries optimized in their corresponding electronic states. The structural RMSD values of the first two triplet states relative to the ground-state geometry are also reported. The relatively larger RMSD of PrPPT in the T_2 state arises mainly from changes in the positions of the propyl substituents..*

	PrPPT			***MePPT***		
	$E_{vertical}$ [eV]	$E_{adiabatic}$ [eV]	Structural RMSD	$E_{vertical}$ [eV]	$E_{adiabatic}$ [eV]	Structural RMSD
S_1	4.2	3.9	0.0908	4.1	3.9	0.0713
T_2	3.7	3.5	0.3033	3.7	3.5	0.0750
T_1	3.0	2.8	0.0332	3.0	2.8	0.0353

3. Fragmentation analysis for PPT compounds

The hole and electron correlations within the manifold of computed excitations (100 singlets and 100 triplets) were analyzed using a hierarchical clustering approach. All computed excited states were examined in terms of excitation localization, resulting in a molecular partitioning based solely on inter-excitation correlations. This partitioning is identical for both singlet and triplet manifolds and clearly separates the PPT backbone from all four propyl chains. When the threshold for clustering is slightly lowered, the propyl chains are further divided into two correlated fragments: one pair located on the NNN face and another on the ONO face. This observation is further supported by representative shapes of the natural transition orbitals involved in the lowest energy transitions, where the electron density is fully delocalized over the **PPT** backbone. The separation of side chains supports the conclusion that substituent modifications can be introduced without significantly affecting the excited-state potentials.

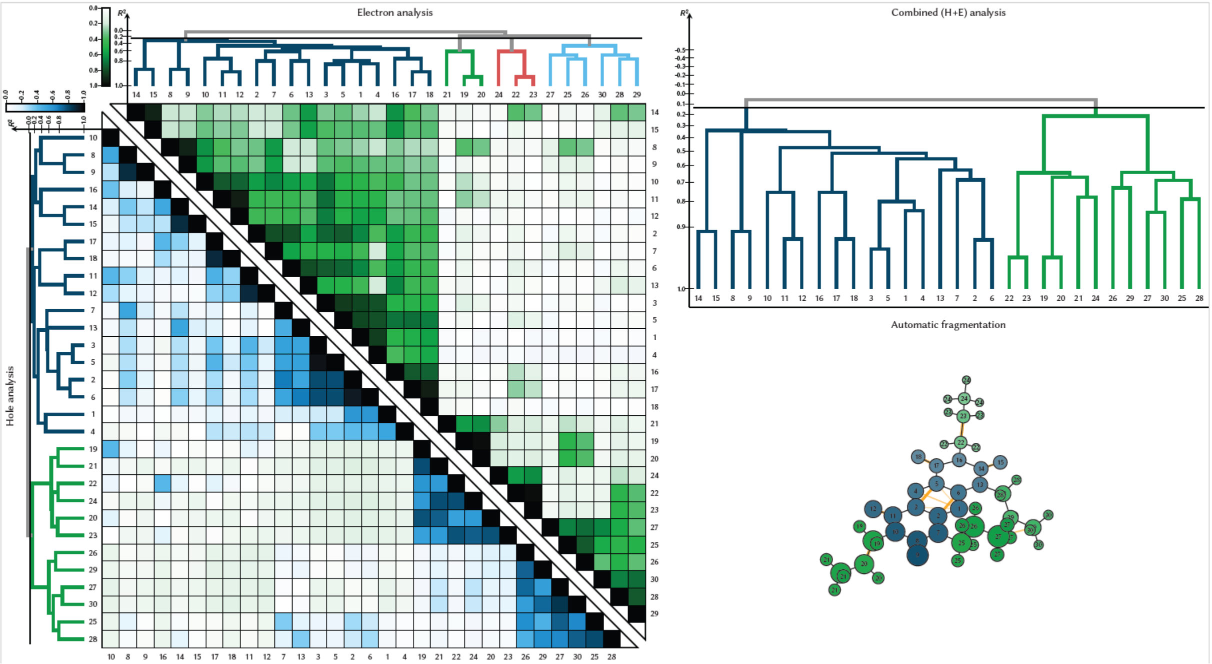

Figure S3: *Hierarchical clustering approach for electron-hole correlations on the example of the singlet manifold for* ***c****. Triplet correlations are similar and omitted for brevity.*

4. ΔG of the PPTH•

4.1. Protonation on ONO vs. NNN-face

As illustrated by the Lewis structure of PrPPT, there are two potential protonation sites on the central ring of the **PPT:** the ONO-face and NNN-face. Both sites can abstract a proton to form the **PrPPTH•** radical, as supported by EPR spectroscopy. However, DFT calculations reveal a significant thermodynamic preference for protonation at the ONO-face site over the NNN-face. This observation is reflected in the Gibbs free energies and electronic energies of the two isomers.

Table S6 summarizes these values for both PrPPT and MePPT, confirming that the protonation at the *ONO*-face is preferable in both cases by approximately 16 kcal/mol.

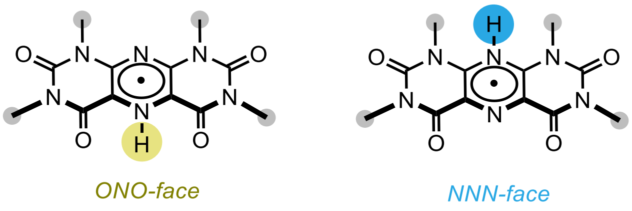

*Figure S4. Lewis structures of **PPTH•** showing the ONO- and NNN-face isomers.*

*Table S6. Gibbs free energies (G in Hartree) and relative free energies (ΔG in kcal/mol) at 298.15 K for ONO and NNN isomers of **PrPPTH•** and **MePPTH•**.*

	ΔG [*kcal/mol*], 298.15 K
PrPPTH•-ONO	0.00
PrPPTH•-NNN	16.22
MePPTH•-ONO	0.00
MePPTH•-NNN	15.65

5. Determination of redox potentials

5.1. Ground-state redox potentials

Cyclic voltammetry (CV) and differential pulse voltammetry (DPV) of **PPTH⁺** were performed at pH 2.36 using a supporting electrolyte solution of 0.1 M $TBAPF_6$ in MeCN. The concentration of **PPTH⁺** was 10 mM. All potentials are reported versus the ferrocene/ferrocenium couple (Fc^+/Fc) as the internal reference and can be referenced to the saturated calomel electrode (SCE) by addition of +0.38 V and to NHE by addition of +0.63 V to the obtained value.[17]

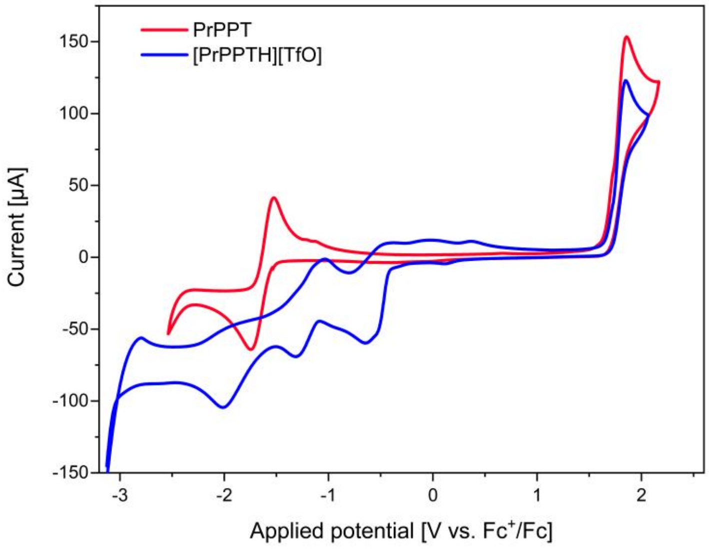

*Figure S5: Cyclic voltammetry of **PrPPT** in MeCN vs. Fc^+/Fc (Red) and their corresponding acidified solution at pH 2.36, representing [**PrPPTH**][OTf] in MeCN vs. Fc^+/Fc (Blue). Concentration = 10 mM. A glassy carbon disk electrode (d = 2 mm) was used as working electrode, a platinum sheet as counter electrode, and Ag/AgCl/LiCl (EtOH) as reference electrode. Potentials are referenced to the Fc^+/Fc couple. Scan rate = 100 mV s^{-1}.*

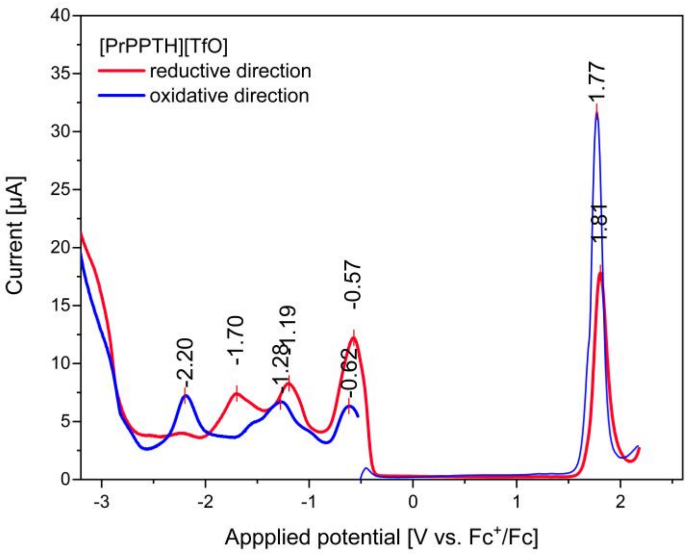

Figure S6: Differential puls voltammetry of [PrPPTH][OTf] in MeCN vs. Fc+/Fc at pH 2.36 at room temperature using a glassy carbon working electrode (d = 2 mm), glassy carbon counter electrode, and Ag/AgCl/LiCl (EtOH) reference electrode. Potentials referenced to Fc^{+}/Fc.

The redox potential of the protonated species **PPTH**$^{+}$ was determined from differential pulse voltammetry (DPV) measurements conducted in both oxidative and reductive directions. To ensure accuracy and minimize potential hysteresis effects, the reported value corresponds to the mean of the peak potentials obtained from the forward (oxidation) and reverse (reduction) scans.

Vs. Fc^{+}/Fc:

$$E_{red}(PPTH^{+}/PPTH^{\bullet}) = \frac{-0.62 + -0.57}{2} V \; = -0.595\,V$$

$$E_{ox}(PPTH^{+\bullet}/PPTH^{+}) = \frac{1.81 + 1.77}{2} V \; = +1.79\,V$$

Vs. SCE:

$$E_{red}(PPTH^{+}/PPTH^{\bullet}) = -0.215\,V$$

$$E_{ox}(PPTH^{+\bullet}/PPTH^{+}) = +2.17\,V$$

Vs. NHE:

$$E_{red}(PPTH^{+}/PPTH^{\bullet}) = +0.03\,V$$

$$E_{ox}(PPTH^{+\bullet}/PPTH^{+}) = +2.42\,V$$

The ground-state redox potential of the corresponding **PPT** was previously obtained by analogous measurements performed by our group, as reported in Ref.[1], and is given as:

Vs. Fc^{+}/Fc:

$$E_{red}(PPT/PPT^{\bullet-}) = -1.59\,V$$

$$E_{ox}(PPT^{+\bullet}/PPT) = +1.80\,V$$

Vs. SCE:

$$E_{red}(PPT/PPT^{\bullet-}) = -1.21\,V$$

$$E_{ox}(PPT^{+\bullet}/PPT) = +2.18\,V$$

Vs. NHE:

$$E_{red}(PPT/PPT^{\bullet-}) = -0.96\,V$$

$$E_{ox}(PPT^{+\bullet}/PPT) = +2.43\,V$$

5.2. Excited-state redox potentials

Excited-state redox potentials (E^*_{ox} and E^*_{red}) were estimated from the ground-state electrochemical data and the singlet excited-state energies according to the Rehm–Weller formalism. Excited-state energies ($E_{0\text{–}0}$) were determined from the intersection of normalized absorption and emission spectra. Excited-state redox potentials were then calculated using $E^*_{red} = E^*_{red} + E_{0-0}$ and $E^*_{ox} = E_{ox} - E_{0-0}$. These values allow estimation of the photoinduced electron-transfer driving forces from the excited states of **PPT** and **PPTH$^+$**. The excited-state redox potential of the protonated species **PPTH$^+$** are listed below:

Vs. Fc^+/Fc:

$$E_{red}(PPTH^{+*}/PPTH^{\bullet}) = -0.595\,V + 3.30\,V \;=\; +2.71\,V$$

$$E_{ox}(PPTH^{+\bullet}/PPTH^{+*}) = +1.79\,V - 3.30\,V \;= -1.51\,V$$

Vs. SCE:

$$E_{red}(PPTH^{+*}/PPTH^{\bullet}) = +3.08\,V$$

$$E_{ox}(PPTH^{+\bullet}/PPTH^{+*}) = -1.13\,V$$

Vs. NHE:

$$E_{red}(PPTH^{+*}/PPTH^{\bullet}) = +3.33\,V$$

$$E_{ox}(PPTH^{+\bullet}/PPTH^{+*}) = -1.38\,V$$

The excited-state redox potential of the corresponding **PPT*** was previously obtained by analogous measurements performed by our group, as reported in Ref.[1], and is given as:

Vs. Fc^+/Fc:

$$E_{red}(PPT^*/PPT^{\bullet-}) = +1.72\,V$$

$$E_{ox}(PPT^{\bullet+}/PPT^*) = -1.51\,V$$

Vs. SCE:

$$E_{red}(PPT^*/PPT^{\bullet-}) = +2.10\,V$$

$$E_{ox}(PPT^{\bullet+}/PPT^*) = -1.13\,V$$

Vs. NHE:

$$E_{red}(PPT^*/PPT^{\bullet-}) = +2.33\,V$$

$$E_{ox}(PPT^{\bullet+}/PPT^*) = -1.38\,V$$

6. Determination of p*K*a values

6.1. Experimental determination of the pKa values for PrPPTH+

The pKa value of **PrPPTH+** was determined using UV-Vis titration with triflic acid (TfOH, p*K*a = 0.7 in MeCN)[18] in acetonitrile. A stock solution of **PrPPT** was prepared at a concentration of [96 µM] in acetonitrile, and 2 mL was transferred to a 4 mL cuvette (d = 1.0 cm). UV-vis spectra were recorded using a [spectrometer make and model], with data acquisition over the wavelength range of 200–600 nm to monitor the absorbance changes of **PrPPT**. The absorbance of the fully deprotonated species **PrPPT** was set to zero, and the formation of the protonated species was recorded as differential spectra.

The titration was performed by adding [10 µL, 20 µL, 40 µL, 80 µL, and 160 µL] of triflic acid solution (concentration: [10 mM & 100 mM]) to the **PrPPT** solution in the cuvette. After each addition, the solution was allowed to equilibrate for [10 seconds] before recording the spectrum. The process was repeated until significant spectral changes were observed, indicating the formation of **PrPPTH+**. To ensure accuracy and reproducibility, all solutions were freshly prepared, and spectra were recorded in duplicate.

Background. In a differential absorbance spectrum, the absorbance is defined as the difference in absorbance between two states (e.g., protonated and deprotonated forms). The general equation follows from Beer-Lambert's law:

$$\Delta A(\lambda) = A_{observed}(\lambda) - A_{reference}(\lambda)$$

where:

- $\Delta A(\lambda)$is the differential absorbance at wavelength λ.
- $A_{observed}(\lambda)$ is the absorbance at a given protonation state (e.g., during titration).
- $A_{reference}(\lambda)$ is the absorbance at a reference state.

For a Protonation Equilibrium:

In the observed titration range, the spectrum follows a two-state equilibrium between the deprotonated form (A) and the protonated form (HA^+). The absorbance at any point during titration can be written as:

$$A(\lambda) = \epsilon_A(\lambda)c_A + \epsilon_{HA+}(\lambda)c_{HA+} \qquad \text{(Beer-Lambert law)}$$

where:

- $\epsilon_A(\lambda)$ and $\epsilon_{HA+}(\lambda)c_{HA+}$ are the molar absorptivities of the neutral and protonated species at λ.
- c_A and $c_{HA+}$are their respective concentrations.

Using the protonation fraction:

$$f_{HA+} = \frac{1}{1 + 10^{pKa-pH}}$$

and

$$f_A = 1 - f_{HA+}$$

The absorbance at any point becomes:

$$A(\lambda) = f_A A_A(\lambda) + f_{HA+} A_{HA+}(\lambda)$$

Normalized Differential Absorbance:

The spectra were normalized such that the fully neutral form has $A_{A,min}(\lambda) = 0$ and the protonated form $A_{A,max}(\lambda) = 1$ and the normalized absorbance can be written as:

$$A_{norm}(\lambda) = \frac{A(\lambda) - A_{A,min}(\lambda)}{A_{A,max}(\lambda) - A_{A,min}(\lambda)}$$

which equals:

$$A_{norm}(\lambda) = \frac{f_{HA+} A_{HA+}(\lambda) + (1 - f_{HA+}) A_A(\lambda) - A_A(\lambda)}{A_{HA+}(\lambda) - A_A(\lambda)}$$

Since the fraction of protonated species follows the Henderson-Hasselbalch equation, this yields:

$$A_{norm} = \frac{1}{1 + 10^{pKa-pH}}$$

Data analysis was conducted by monitoring the absorbance at 363 nm, where changes in absorbance reflected the protonation equilibrium. The normalized differential absorbance values A_{norm} were plotted as a function of acid concentration with $pH = -\log{[TfOH]}$. The resulting titration curve was analyzed to determine the pKa of the corresponding acid of the analyte **PrPPTH**$^{+}$. The pKa value was obtained from the inflection point of the titration curve at $A_{norm} = 0.5$ of the sigmoidal fit function.

Table S7: Spectrophotometric titration of PrPPT with TfOH.

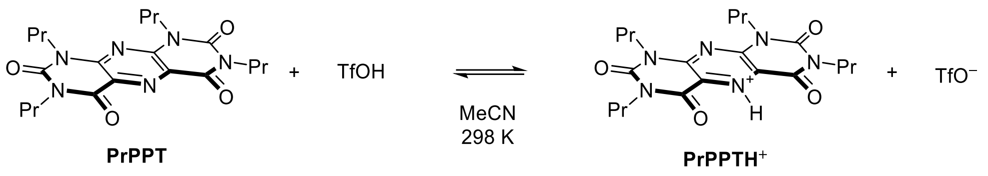

	1st run		2nd run	
	−log[TfOH]	ΔA_{norm}[PPTH+]	−log[TfOH]	ΔA_{norm}[PPTH+]
1	3.75	0.057	3.70	0.0276
2	3.421	0.1470	3.42	0.1087
3	3.151	0.3030	3.15	0.2923
4	2.907	0.5772	2.91	0.5765
5	2.702	0.9434	2.70	0.9684
6	2.5	0.9642	2.50	0.9921
7	2.367	1.0037	2.37	0.9321
8	2.13	1	2.13	1

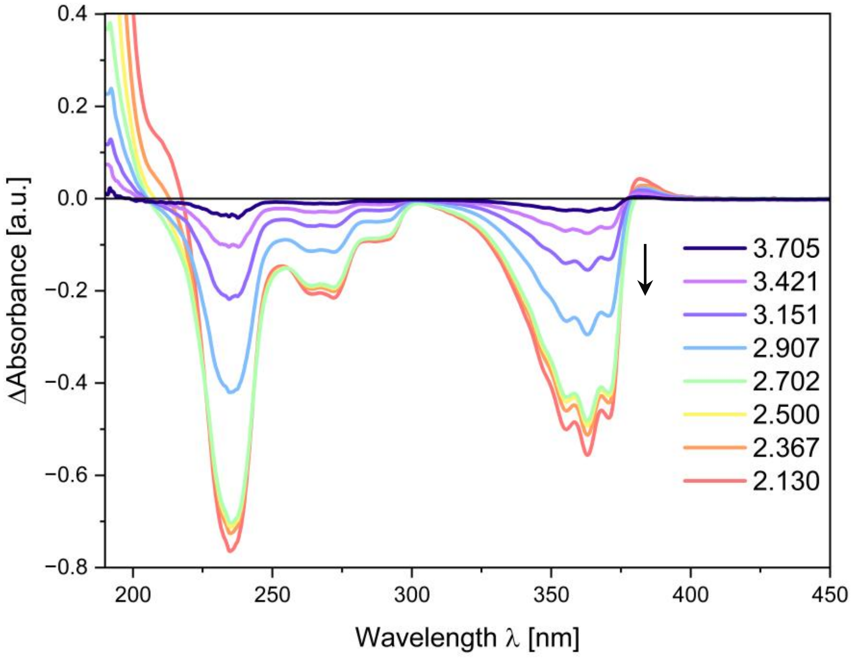

Figure S7: Differential UV-Vis spectra for the spectrophotometric titration of ***PrPPT*** *with TfOH.*

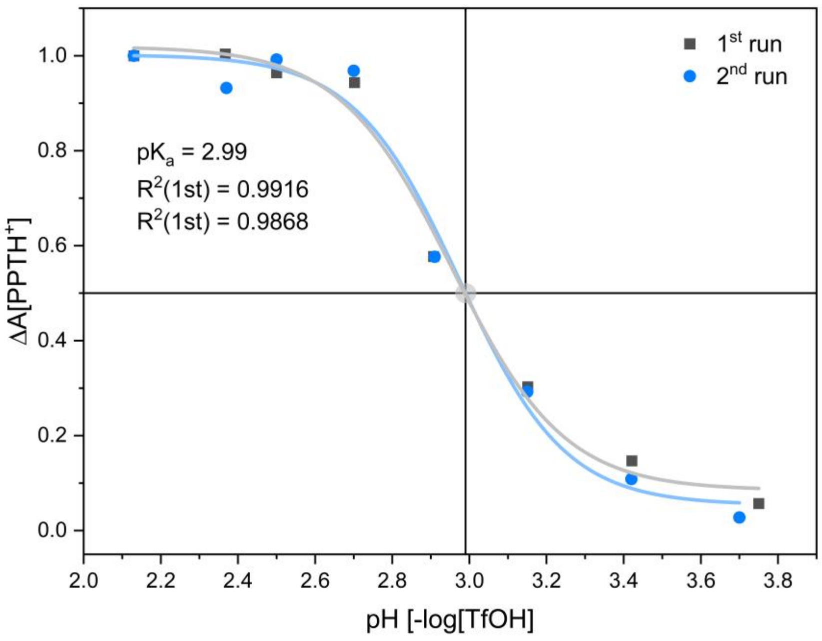

*Figure S8: Titration curve of **PrPPT** with TfOH in MeCN*

6.2. Computational estimation of the pK_a values

The calculation of the acid dissociation constant (p*K*a) using DFT involves evaluating the relative free energies of the protonated acid and its conjugate base in solution. This computational approach leverages the free energy differences between protonated and deprotonated species, providing a quantitative estimate of the p*K*a. By combining reaction free energies (ΔG) with experimental pKa values of reference acids, it is possible to predict the p*K*a of unknown compounds through linear free-energy relationships.

In this study, the p*K*a values of the target molecules, including the acidic form of **PrPPT** (**PrPPTH**$^+$) as well as the neutral radical species **PrPPTH**$^\bullet$, were estimated through acid-base equilibria involving reference acids (acetic acid, benzoic acid, 2,4-$(NO_2)_2$phenol, 3-$CF_3C_6H_4CH(CN)_2$, and Tosic acid). The experimentally determined p*K*a values of these reference acids (ranging from 8 to 24 in MeCN)[19] were used to construct a calibration curve.

The reaction Gibbs free energies (ΔG) for the acid-base equilibria were calculated according to the following equations:

$$PPTH^{+}/\ PPTH^{\bullet} + \ A^{-} \rightleftharpoons PPT/\ PPTH^{\bullet -} + \ AH$$

The p*K*a of the target molecule was estimated using the following equation:

$$pK_a^{target} = pK_a^{reference} - 2.303RT\ \Delta G^{\circ}$$

Linear free-energy relationships were established by plotting the calculated ΔG values (in kcal/mol, MeCN) against the experimental p*K*a values of the reference acids in the same solvent. The corresponding linear free energy plots are shown in Figure SS9-Figure SS10, where the x-axis is the calculated reaction free energy (ΔG_{prot}) of the equilibrium in MeCN and the y-axis is reported pK_a values.

Table S8. pKa values (in MeCN) of reference acids used for the estimation of the pKa of ***PrPPTH***$^+$ *and* ***PrPPTH***$^\bullet$*.*[19]

Reference Acid	*pKa (in MeCN)*
Acetic acid	23.51
Benzoic acid	21.51
2,4-$(NO_2)_2$phenol	16.66
3-$CF_3C_6H_4CH(CN)_2$	14.72
Tosic acid	8.60

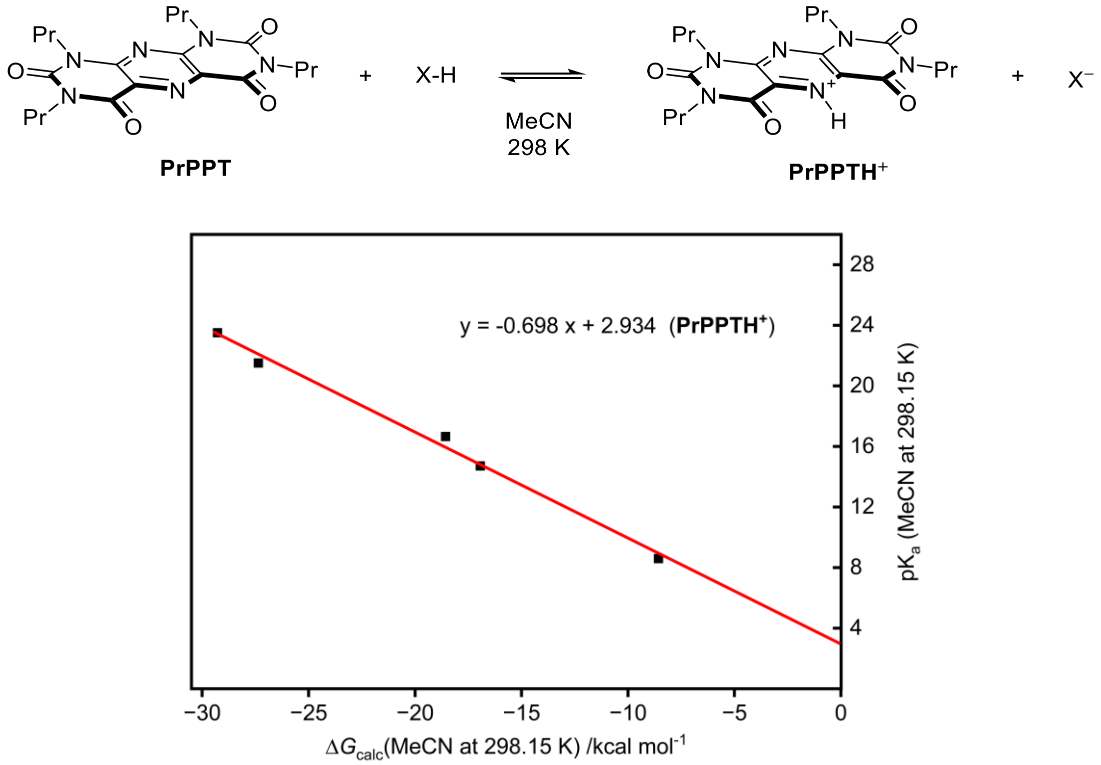

*Figure SS9: Linear free energy plots of **PrPPT** with different acids. pKa (MeCN) of **PrPPT** is estimated at 2.9.*

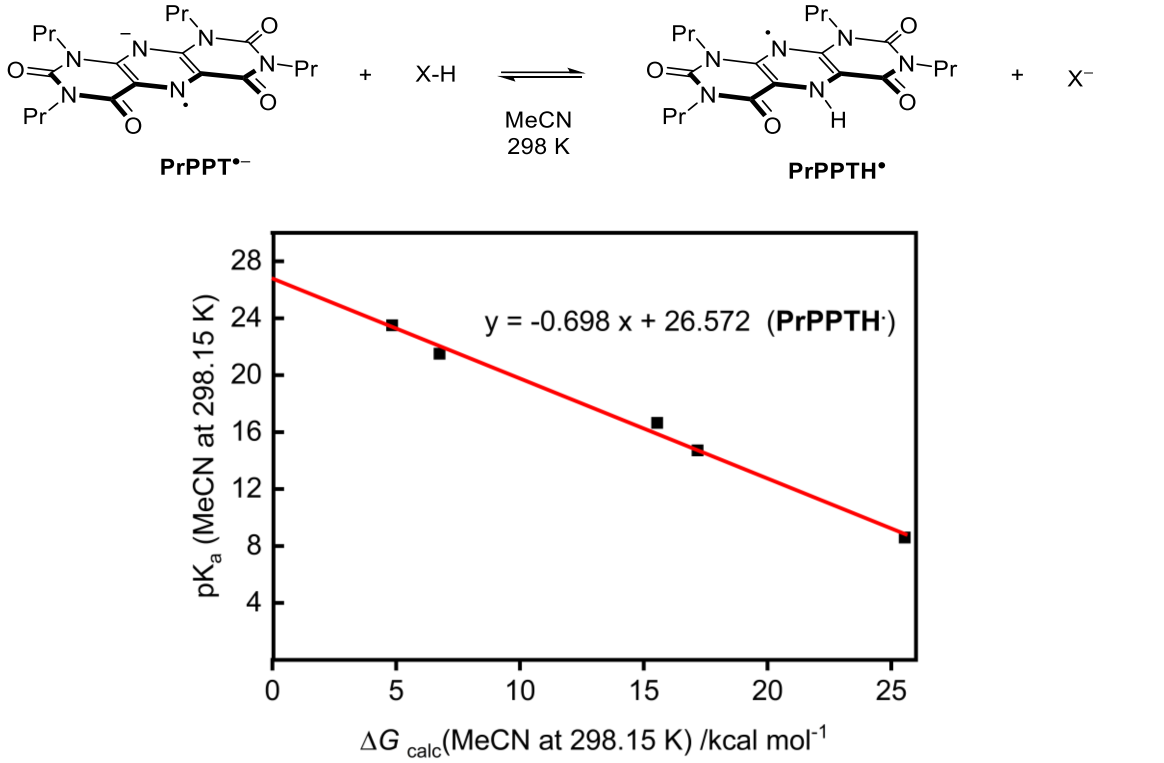

*Figure SS10: Linear free energy plots of **PrPPT•−** with different acids. pKa (MeCN) of **PrPPTH•** is estimated at 26.6.*

7. Determination of pK_a*

The excited-state acidity constant (pK_a*) of **PPTH^{+}*** was estimated using the Förster cycle, which relates the ground- and excited-state acid–base equilibria through the thermodynamic cycle. The pK_a value in the ground state was determined independently (see section 7), and the 0–0 transition energies ($^{1}E_{0,0}$) of the neutral and protonated species were obtained from the intersection of their normalized absorption and emission spectra ($E_{0,0}^{PPT} = +3.31\ eV$ and $E_{0,0}^{PPTH^+} = +3.30\ eV$; see section 3. The p$K_a$* was then calculated using the Förster equation:

$$pK_a{}^{*} = \frac{\Delta G^*}{2.303 \cdot RT}$$

where

$$\Delta G^* = E_{0,0}^{PPT} + \Delta G - E_{0,0}^{PPTH^+}$$

and

$$\Delta G = 2.303\ RT \cdot pK_a$$

with $R = 8.617 \cdot 10^{-5}\ eV/K$ and $T = 298.15\ K$.

$$E_{0,0}^{PPT} = +2.29\ eV$$

$$E_{0,0}^{PPTH^+} = +1.84\ eV$$

$$pK_a = 2.99 \text{ in MeCN}$$

$$\Delta G = 2.303 \cdot 8.617 \cdot 10^{-5} \frac{eV}{K} \cdot 298.15\ K \cdot 2.99 = 0.177\ eV$$

$$\Delta G^* = [3.31 + 0.177 - 3.30] eV = 0.187\ eV$$

$$pK_a{}^{*} = \frac{\Delta G^*}{2.303 \cdot RT} = \frac{0.187\ eV}{2.303 \cdot 8.617 \cdot 10^{-5}\ eV/K \cdot 298.15\ K} = 3.16$$

8. Bond dissociation free energies of the exocyclic PPTH$^{\bullet}$ N-H bond

8.1. Square Scheme Sanalysis

To elucidate the thermodynamic relationships between proton and electron transfer events, a square Scheme Sanalysis was conducted following the approach established by Bordwell and Breslow and advocated by Mayer and co-workers.[20] This method enables estimation of bond dissociation free energies (BDFEs), pK_a values, and redox potentials for proton-coupled electron transfer (PCET) systems. By integrating the experimentally determined as well as computed values, the free energies of the individual steps (proton transfer (PT), electron transfer (ET), and concerted proton-electron transfer (cPCET)) were extracted. Ground-state redox potentials are referenced to SCE. The analysis presented here provides a consistent framework for evaluating the thermodynamic parameters of the **PPT** framework. Excited-state redox potentials were calculated according to the following equation:

$$E_T^*(PPT/PPT^{\bullet-}) = E_{red}^{1/2}(PPT/PPT^{\bullet-}) + E_T\ (\,^3PPT\ \ )$$

and

$$E_T^*(\,^3PPTH^{+*}/PPTH^{\bullet}) = E_{red}^{1/2}(\,^3PPTH^{+*}/PPTH^{\bullet}) + E_T\ (\,^3PPTH^{+})$$

Table S9. Overview of data for the thermochemical box scheme.

	**Fc^+/Fc**	**SCE**	**NHE**	**unit**
p*K*a (**$PPTH^+$**)	2.99	2.99	2.99	in MeCN
**$PPTH^+$/$PPTH^{\bullet}$**	-0.60	-0.22	0.03	eV
**BDFE**	**42.86**	**51.62**	**57.39**	**kcal·mol^{-1}**
**PPT/$PPT^{\bullet-}$**	-1.59	-1.17	-0.96	eV
pKa (**$PPTH^{\bullet}$**)	19.65	18.98	19.65	in MeCN
E(S_1)	3.31	3.31	3.31	eV
**^{1}PPT*/$PPT^{\bullet-}$**	1.72	2.14	2.35	eV
**^{1}BDFE***	**111.32**	**123.32**	**131.22**	**kcal·mol^{-1}**
^{3}E(T_1)	2.11	2.11	2.11	eV
**^{3}PPT*/$PPT^{\bullet-}$**	0.52	0.94	1.15	eV
**^{3}BDFE***	**91.52**	**100.28**	**106.04**	**kcal·mol^{-1}**
E(S_1)	3.30	3.30	3.30	eV
**$^1PPTH^{+*}$/$PPTH^{\bullet}$**	2.70	3.08	3.33	eV
p*K*a (**$^1PPTH^+$**)	3.16	3.16	3.16	in MeCN
E(T_1, **$PPTH^+$**)	1.88	1.88	1.88	eV
**$^3PPTH^{+*}$/$PPTH^{\bullet}$**	1.28	1.66	1.91	eV
p*K*a (**$^3PPTH^+$**)	6.86	6.86	6.86	in MeCN

The combination of (i) excited-state energies, (ii) ground- and excited-state reduction potentials, and (iii) the calculated pK_a of PPTH (as determined in Section 7) allows for the calculation of effective bond dissociation energies using the Bordwell–Breslow formalism:

$$BDFE = 1.37\ pK_a + 23.06\ E_{red}^{1/2} + C_g$$

$$\text{with } C_g = 52.6\ kcal/mol \text{ at 298 K in MeCN.}$$

The calculated BDFEs are dependent on the reference electrode used for the redox potentials. As such, BDFE values can vary across different reference systems. To ensure transparency and comparability, all BDFEs referenced against commonly used systems (Fc^+/Fc, SCE, NHE) are compiled in Table S9. The corresponding thermochemical box schemes for each reference system are illustrated in Figure S11-Figure S13.

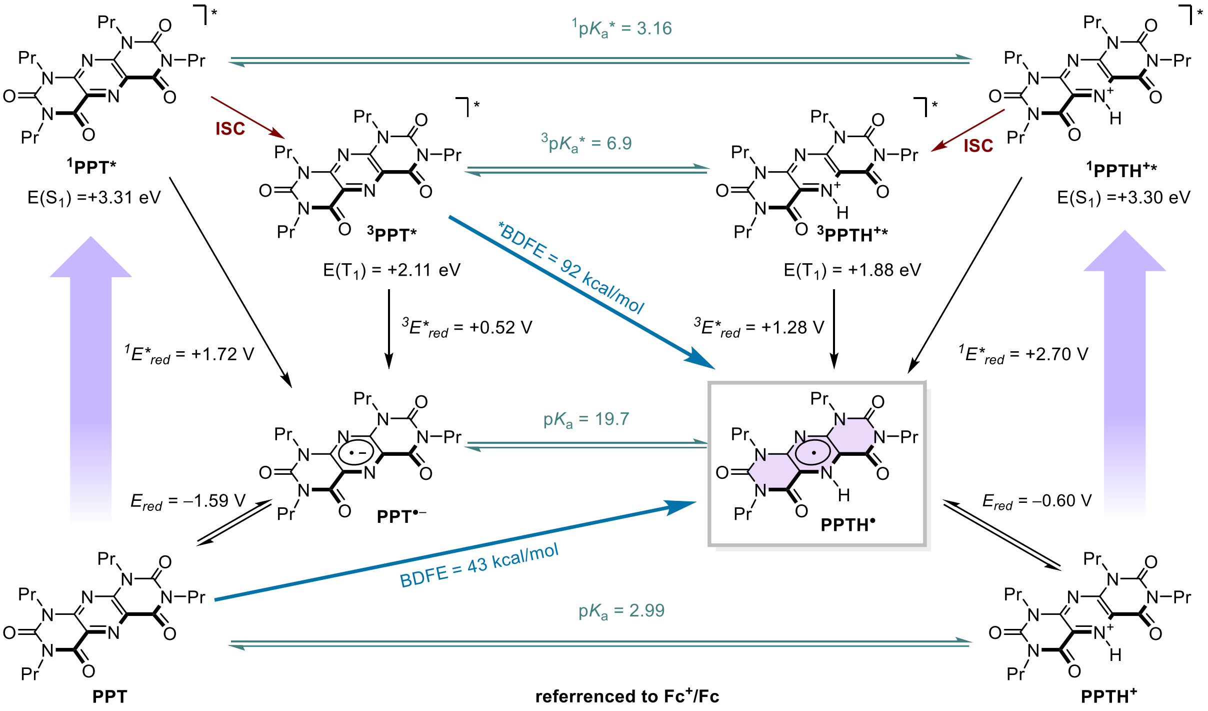

*Figure S11. Thermochemical energy box Scheme Sof **PPT** and **PPTH**$^+$, including triplet state energies and BDFE referenced to Fc$^+$/Fc.*

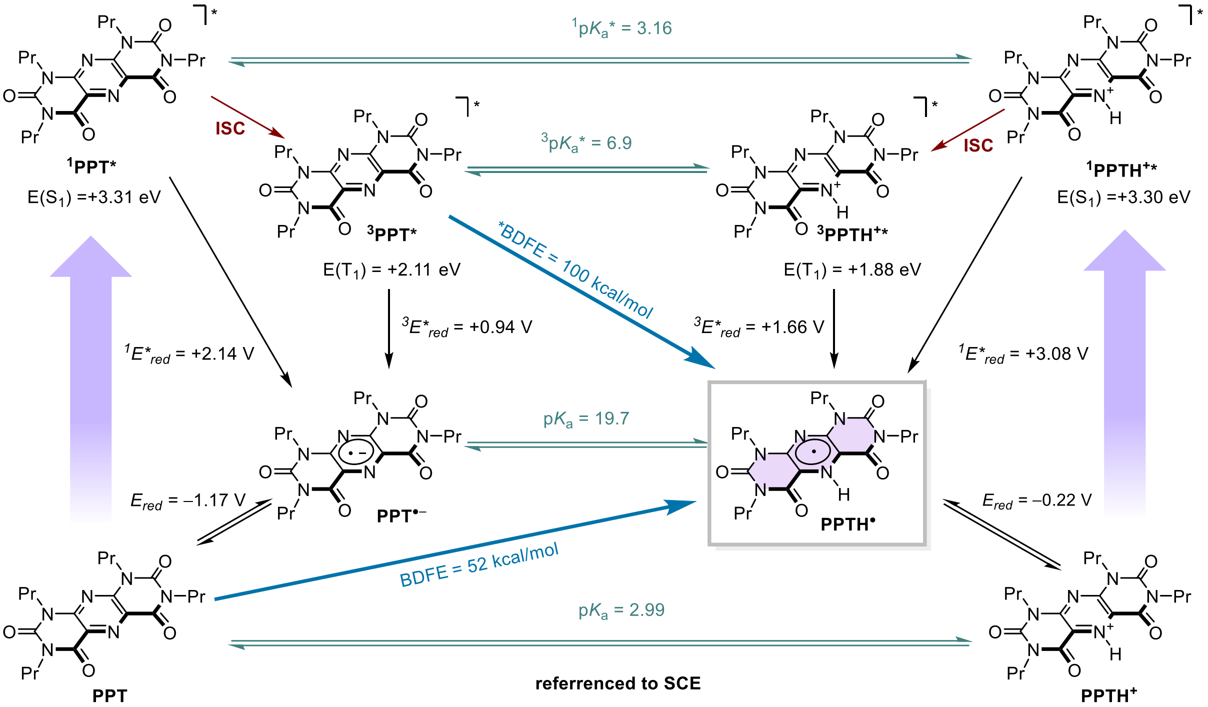

*Figure S12. Thermochemical energy box Scheme Sof **PPT** and **PPTH**$^+$, including triplet state energies and BDFE referenced to SCE.*

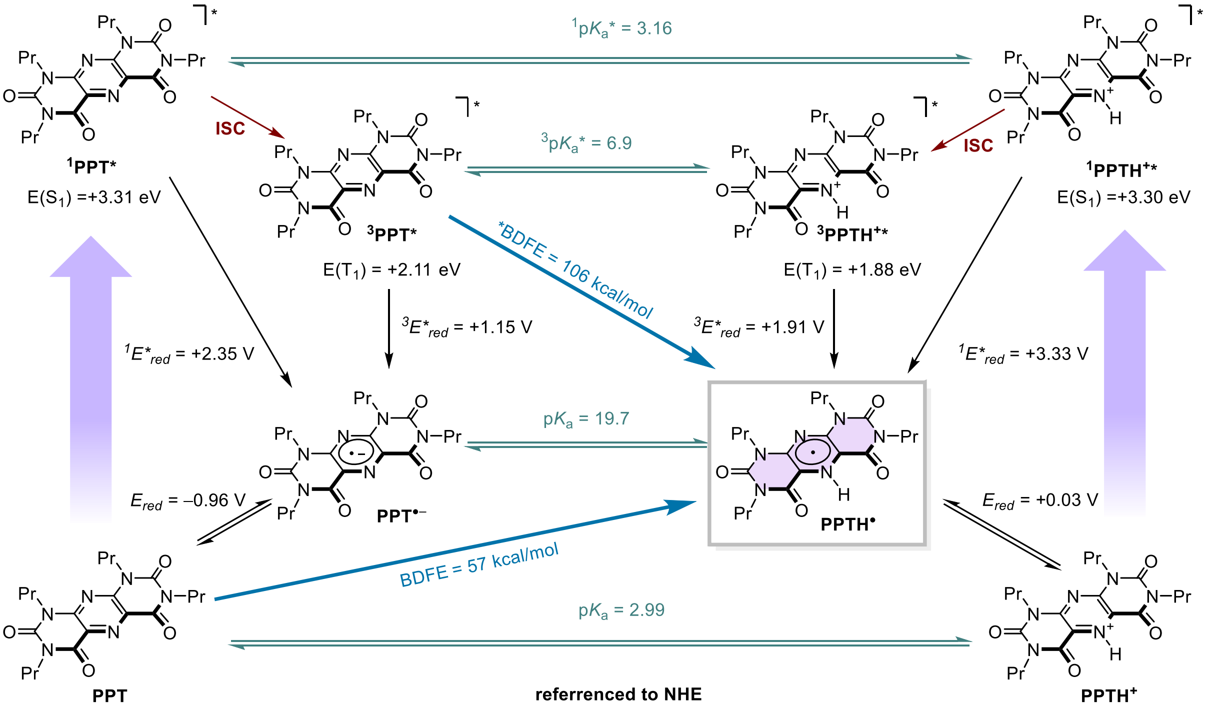

*Figure S13: Thermochemical energy box Scheme Sof **PPT** and **PPTH**$^+$, including triplet state energies and BDFE referenced to NHE.*

8.2. Computational determination of the BDFE of PPTH•

The bond dissociation free energy (BDFE) of the exocyclic N–H bond in **PrPPTH•** was estimated theoretically using DFT calculations at 298.15 K. To ensure accuracy, the calculated BDFE values were benchmarked against known experimental bond strengths, specifically, butane (91.0 kcal/mol) and ethylbenzene (78.9 kcal/mol).

The BDFE of **PrPPTH•** was calculated using the following relation:

$$BDFE_{PPTH^{\bullet}} = \Delta G^{\circ}_{HAT} + BDFE_{ref}(X-H)$$

where ΔG°_{HAT} represents the standard Gibbs free energy change for the HAT reaction, as illustrated below:

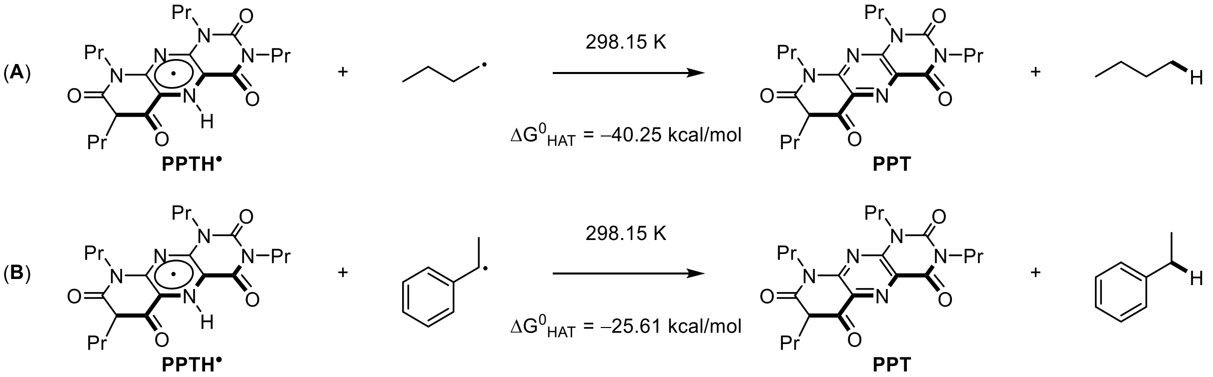

Thus, BDFE (**PrPPTH•**) is theoretically estimated to be:

$$BDFE_{PPTH^{\bullet}} = -40.25 + 91.00 = 50.75 \text{ kcal/mol}$$

and

$$BDFE_{PPTH^{\bullet}} = -25.61 + 78.90 = 53.29 \text{ kcal/mol}$$

based on butane and ethylbenzene, respectively.

9. EPR spectroscopy

EPR spectra were recorded on an X-band BRUKER EMX CW-micro EPR spectrometer equipped with an ER4119HS high-sensitivity resonator using a microwave power of 6.9 mW, modulation frequency of 100 kHz, and modulation amplitude up to 5 G. The $h\nu = g\beta B_0$ equation was used to calculate g values with ν and B_0 being the frequency and resonance field, respectively. 2,2-Diphenyl-1-picrylhydrazyl g values calibration was performed using 2,2-Diphenyl-1-picrylhydrazyl as a standard ($g = 2.0036 \pm 0.0004$). EPR spectrum simulation was done by MATLAB R2023a using the EasySpin-5.2.36 module.

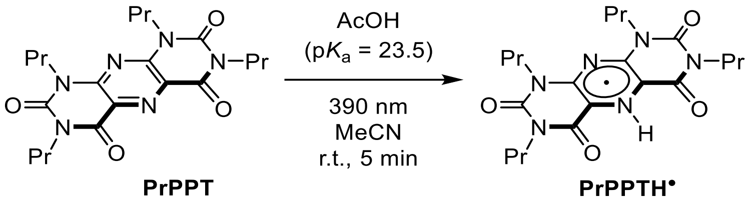

Scheme S1. Photo-mediated generation of PPTH• from weak acids.

The reaction was performed under Schlenk conditions using a septum-sealed EPR flat cell. The photocatalyst PrPPT (52 mg, 0.125 mmol) and acetic acid (75 mg, 1.25 mmol, 10 equiv.) were dissolved in dry acetonitrile (1.0 ml). The mixture was irradiated at 390 nm (Kessil lamp) without stirring while operando EPR-spectroscopy.

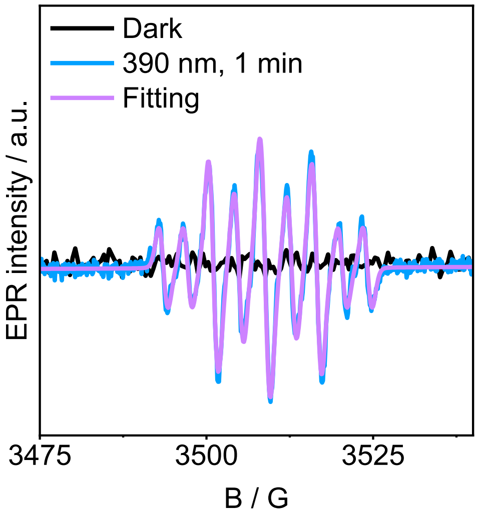

Figure S14: PrPPT with AcOH in MeCN in dark (black), under irradiation (40 W Kessil lamp 390nm), (a modulation amplitude of 1 G)(blue) and the simulated spectra with hyperfine splitting from two nonequivalent N and one H (A_{N1} = 7.91 G, A_{N2} = 3.69 G, A_H = 7.46 G)(pink).

10. Analysis of reaction pathways

10.1. Discussion of PPTH• formation

The computed intersystem-crossing (ISC) rate constants from the S_1 state to T_2 are 5.4×10^8 s^{-1} and 3.2×10^9 s^{-1} for **MePPT** and **PrPPT**, respectively, corresponding to characteristic ISC times of approximately 1.8 ns and 0.3 ns. In comparison, the calculated fluorescence rate constants from S_1 are 7.6×10^8 s^{-1} for **MePPT** and 7.1×10^8 s^{-1} for **PrPPT**, corresponding to fluorescence lifetimes of about 1.3 ns and 1.4 ns, respectively. These values indicate that ISC can compete efficiently with fluorescence and other S_1 decay channels. This competition is especially pronounced for **PrPPT**, for which ISC is significantly faster than fluorescence and is therefore expected to redirect a substantial fraction of the excited-state population into the triplet manifold. Under typical photocatalytic concentrations, diffusion-controlled electron transfer can still reach effective rates on the order of 10^8–10^9 s^{-1}, so for **MePPT**, a productive SET directly from S_1 remains kinetically accessible. Overall, these results suggest that the productive SET step depends on the substitution pattern: **MePPT** may plausibly react from the singlet excited state, whereas **PrPPT** is more likely to involve the triplet manifold because of its markedly faster ISC.Energetics of radical-radical coupling reactions

The coupling reactions between the **PPTH•** and **Sub•** involve the interaction of two unpaired electrons. These reactions can proceed via two distinct spin channels: a singlet surface (S = 0, antiparallel spins) leading to the formation of a covalent bond, and a triplet surface (S = 1, parallel spins), which is generally repulsive or leads to high-energy levels.

To accurately describe the energetics of these pathways, Unrestricted Kohn-Sham Density Functional Theory (UKS-DFT) calculations were performed. While the triplet encounter is well-described by a single determinant, the singlet encounter in radical-radical reactions often exhibits significant open-shell diradical character, particularly in the reactant complex (RC) and transition state (TS) regions. This multi-reference character cannot be captured by standard closed-shell Restricted DFT (R-DFT). Consequently, the Broken Symmetry (BS) approach was employed to model the singlet pathway.

However, the BS solution (E_{BS}) is not a pure singlet state; it is often contaminated by higher-spin states (primarily the triplet state), resulting in an expectation value of the spin operator $\langle S^2\rangle_{BS}$, that deviates from the ideal value of 0. To address spin contamination and derive the energy of the pure singlet state, we applied the approximate spin-projection Scheme Sproposed by Yamaguchi and co-workers.[21, 22] For the radical-radical coupling, the Singlet-Triplet energy gap (ΔE_{ST}) was calculated using the relationship:

$$\Delta E_{ST} = \frac{2(E_{BS} - E_T)}{\langle S^2\rangle_T - \langle S^2\rangle_{BS}}$$

where E_{BS} and E_T are the energies of the broken-symmetry singlet and the triplet states, respectively, and $\langle S^2\rangle_{S/T}$ are the corresponding spin angular momentum expectation values. The factor of 2

corresponds to the ideal spin expectation value of the triplet state ($\langle S^2 \rangle_T \approx 2$). This correction ensures that the reported energetics for the singlet pathway accurately reflect the pure spin state.

Figure S15 shows the calculated potential energy surfaces for the coupling of **PPTH•** with radicals A, B, and C, which are schematically represented in Scheme S2. The energy profile includes the Separated Radicals (SR), Reactant Complexes (^{1}RC/^{3}RC), Transition States (^{1}TS/^{3}TS), and Products (^{1}PR/^{3}PR). All energies are reported relative to the separated reactants (SR) at 0.0 kcal.mol^{-1}.

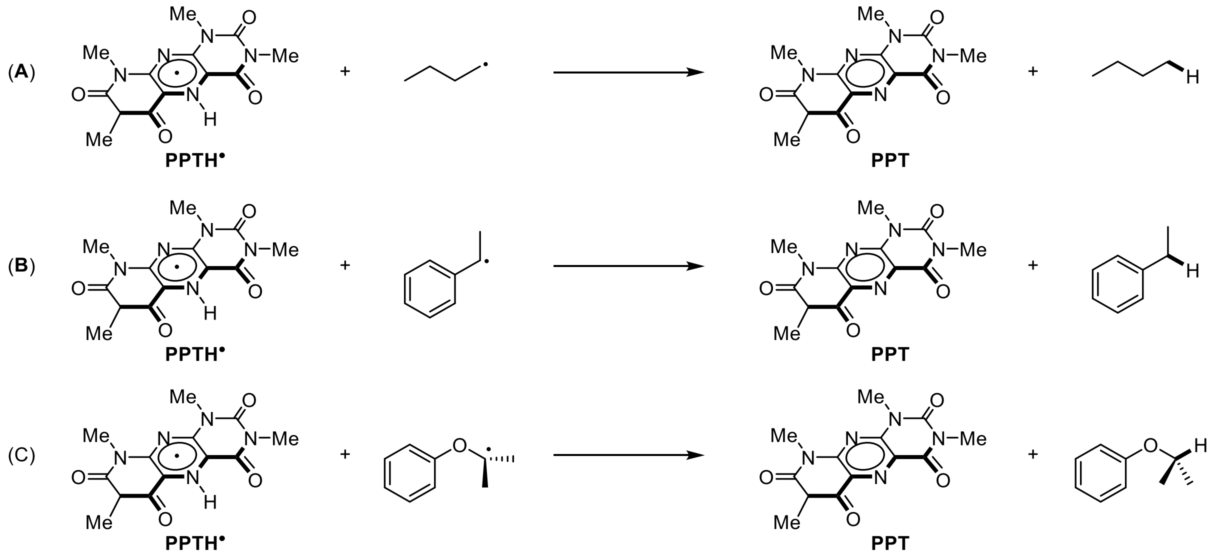

Scheme S2. Overview of reactions concerning the IBO analysis along the IRC.

Table S9. Relative electronic energies (ΔE_{el}, kcal.mol^{-1}) and solution-phase Gibbs free energies (ΔG, kcal.mol^{-1}) for the radical–radical coupling of PPTH• with (A) n-butyl, (B) benzyl, and (C) phenoxy radicals along the spin-projected broken-symmetry singlet (^{1}BS) and triplet (^{3}T) pathways. Energies are referenced to the separated radicals (SR) set to 0.0. RC = reactant complex, TS = transition state, and PR = product. Imaginary frequencies for the TS structures are reported in cm^{-1}.

	PPTH• with n-butyl (A)			***PPTH• with benzyl (B)***			***PPTH• with phenoxy (C)***		
	ΔE_{el}	ΔG	***Freq.***	ΔE_{el}	ΔG	***Freq.***	ΔE_{el}	ΔG	***Freq.***
SR	0.0	0.0		0.0	0.0		0.0	0.0	
^{1}RC	-7.4	5.4		-12.8	1.9		-11.1	3.3	
^{1}TS	-1.6	11.1	-1374.9	-2.9	11.5	-1656.1	-5.7	9.1	-1178.6
^{1}PR	-53.3	-37.2		-43.3	-26.4		-52.5	-34.9	
^{3}RC	-6.6	5.3		-11.0	2.8		-11.1	2.6	
^{3}TS	25.1	37.1	-487.8	32.6	44.9	-295.8	22.2	34.3	-1822.4
^{3}PR	10.1	22.9		20.4	33.9		11.4	25.3	

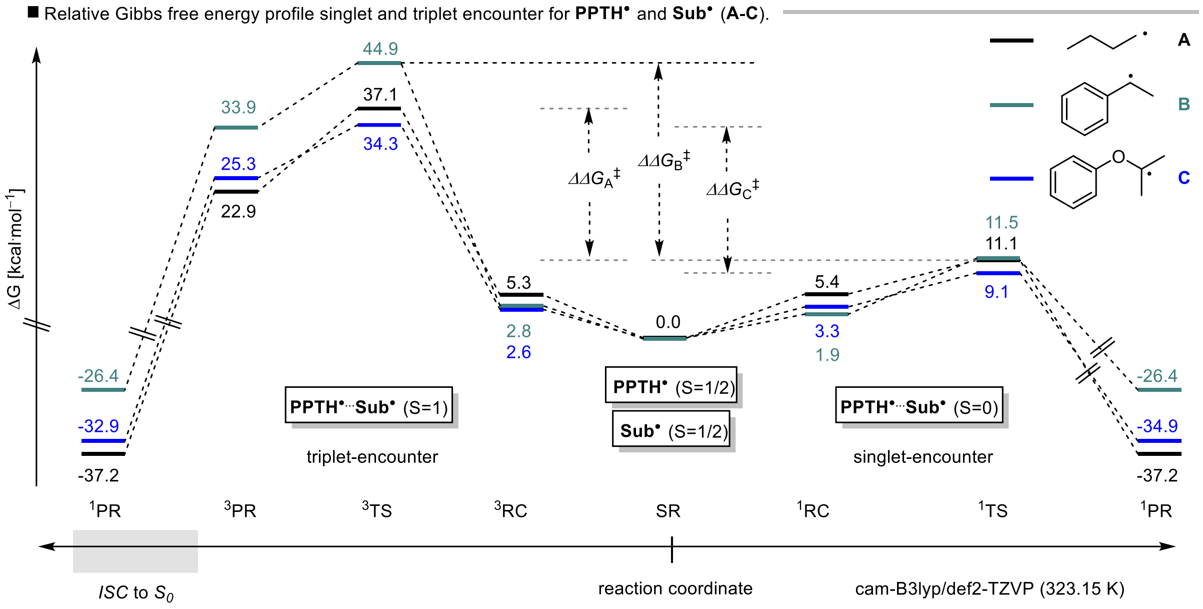

*Figure S15. Calculated Gibbs free-energy profiles (ΔG) for the radical-radical coupling of **PPTH•** with (A) n-butyl, (B) benzyl, and (C) phenoxy radicals. The profiles compare the energetics of the spin-projected broken-symmetry singlet (^1BS) and triplet (^3T) pathways. $\Delta\Delta G^{\ddagger}$ represents the difference in reaction barriers between the singlet and triplet states.*

10.2. IBO analysis

The IBO analysis was carried out for both singlet and triplet pathways of the reaction of **PPTH•** with (A) n-butyl radical, (B) benzyl radical, and (C) phenoxy radical. For the singlet pathway (dominant one, see main text),

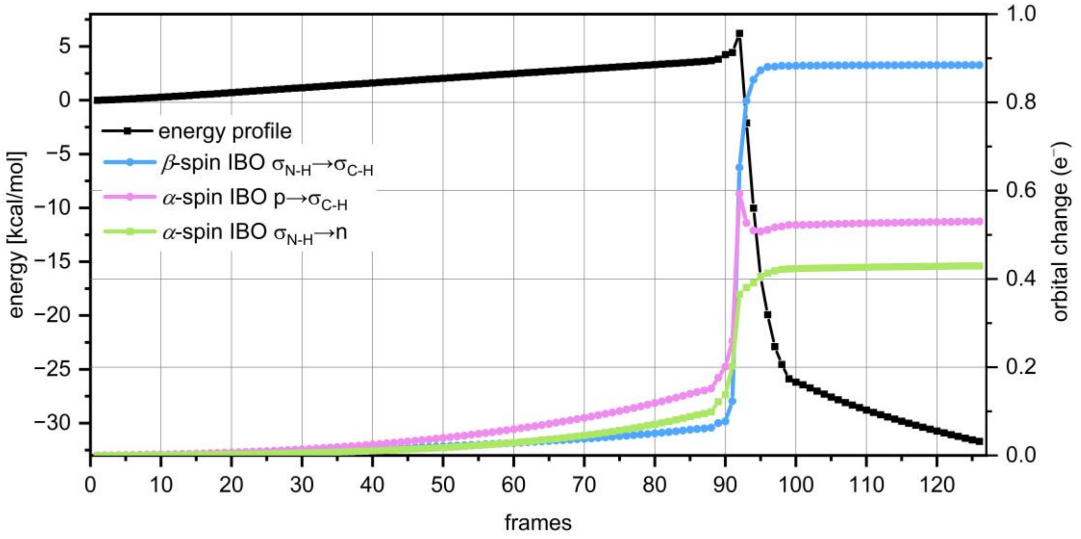

Figure S16, S19, and S21 present the IRC profiles of each radical combination together with the corresponding α- and β-spin IBOs that undergo significant changes along the path. Figure S17-Figure S21 illustrate representative orbital reorganizations, highlighting the movement and redistribution of the α- and β-spin IBOs along the IRC. Analogous analysis for the triplet pathway is given in section 10.2.2.

10.2.1. Singlet pathway

For all studied radical substrates (see Figures S17-S22), the reaction proceeds via a simultaneous transfer of a proton and an α-spin electron from the PPTH• radical, constituting a direct H• transfer. Specifically, the α electron from the σ(N–H) bonding orbital is transferred into the p-orbital of the substrate radical carbon, where it pairs with the *β*-spin electron to form the new C–H bond. Concurrently, the α-spin electron initially residing in the σ(N–H) orbital remains localized on the PPTH, contributing to the N5 lone-pair. The concerted nature of proton and electron transfer in this step provides strong evidence for a dHAT mechanism.

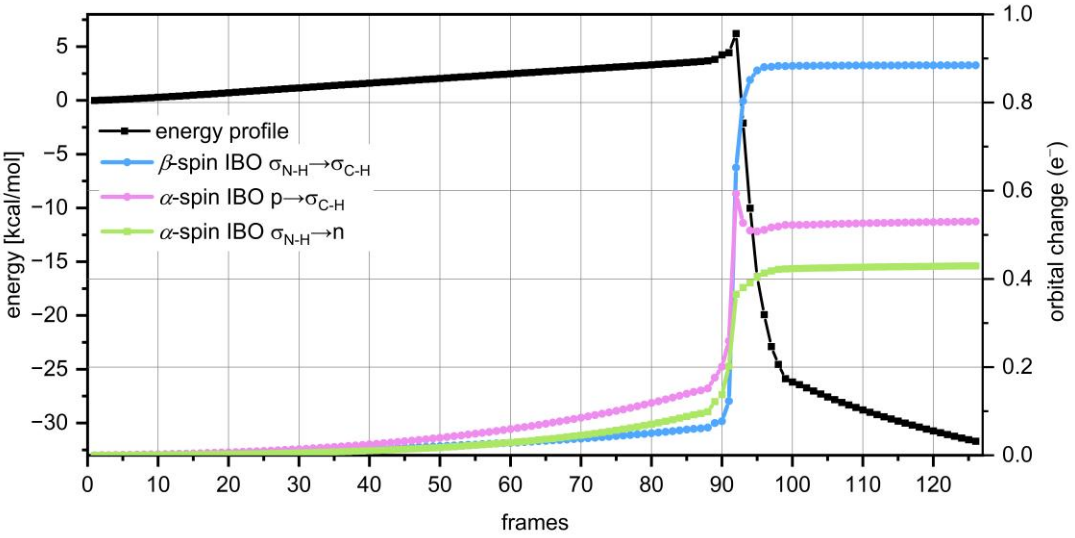

Figure S16. Intrinsic reaction coordinate (IRC) profile for the singlet pathway of the reaction of PPTH radical with n-butyl radical. Selected α- and β-spin IBOs showing significant changes along the IRC are indicated.

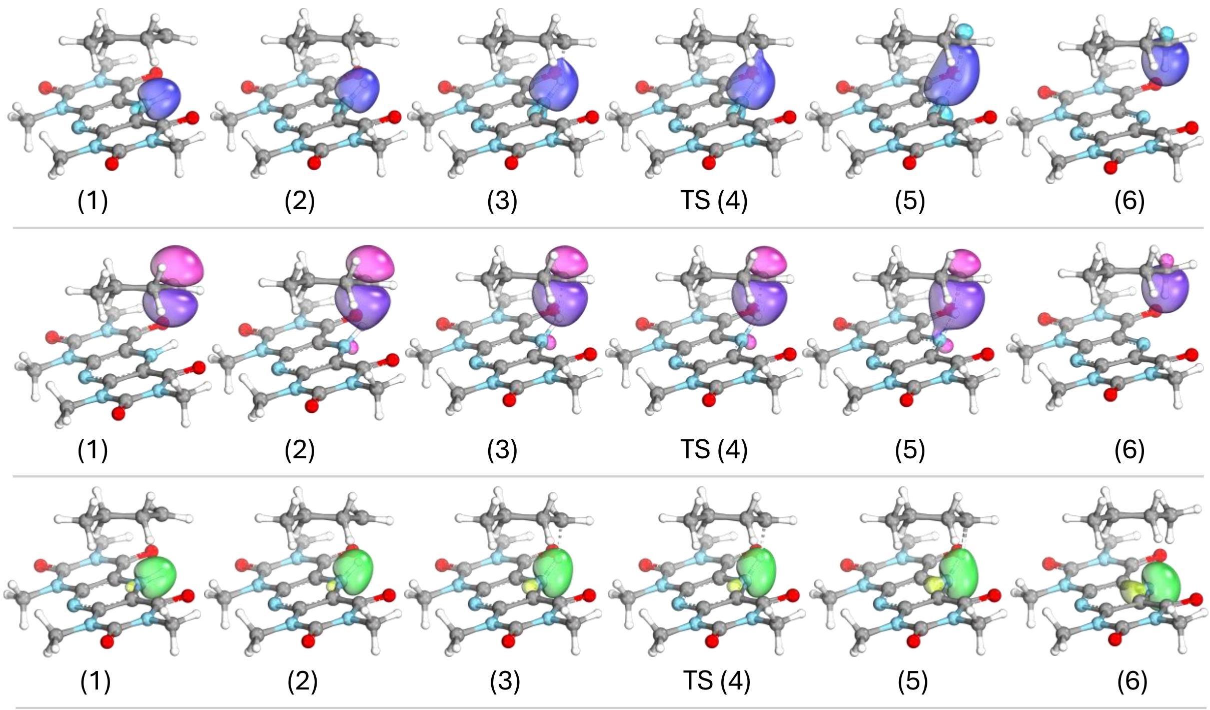

Figure S17. Representative α- and β-spin IBOs at selected points along the IRC for the PPTH–n-butyl radical reaction (singlet pathway), illustrating orbital reorganization during HAT.

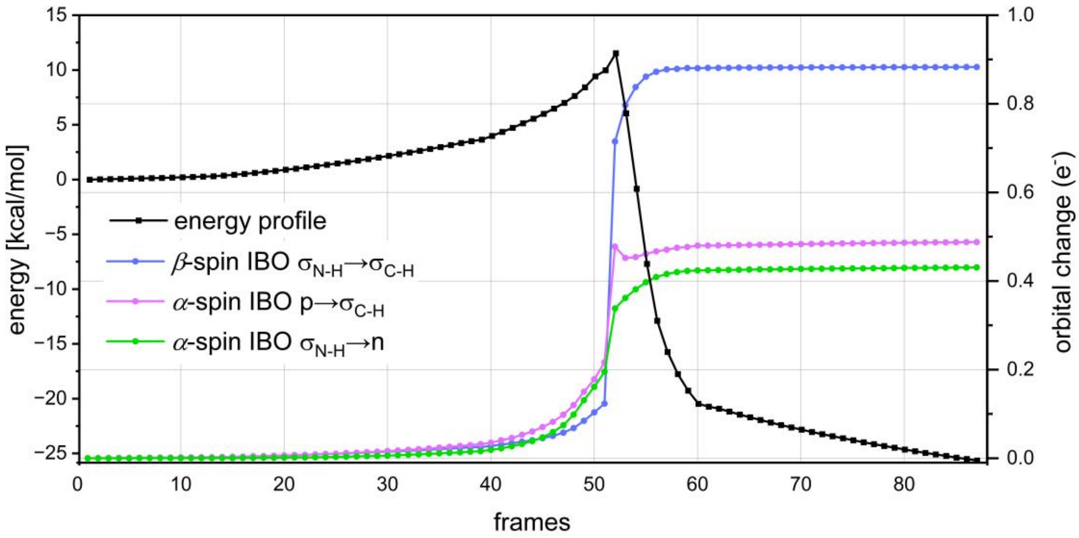

Figure SS18. Intrinsic reaction coordinate (IRC) profile for the singlet pathway of the reaction of PPTH radical with benzyl radical. Selected α- and β-spin IBOs showing significant changes along the IRC are indicated.

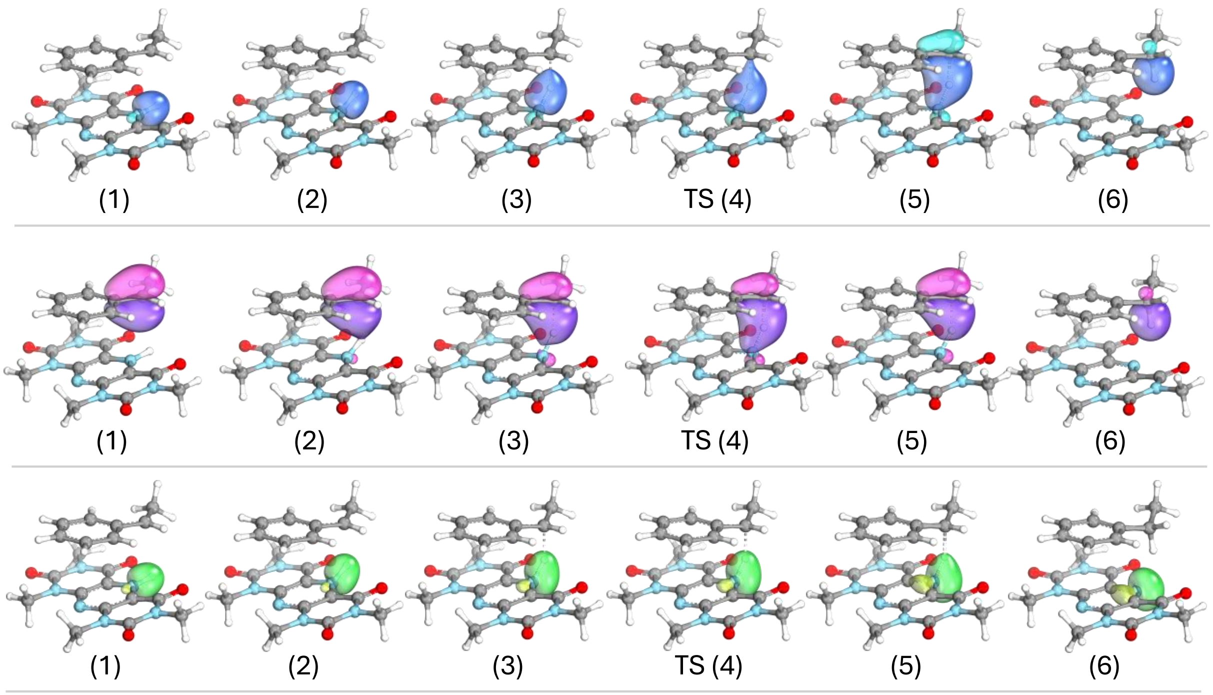

Figure S19. Representative α- and β-spin IBOs at selected points along the IRC for the PPTH–benzyl radical reaction (singlet pathway), illustrating orbital reorganization during HAT.

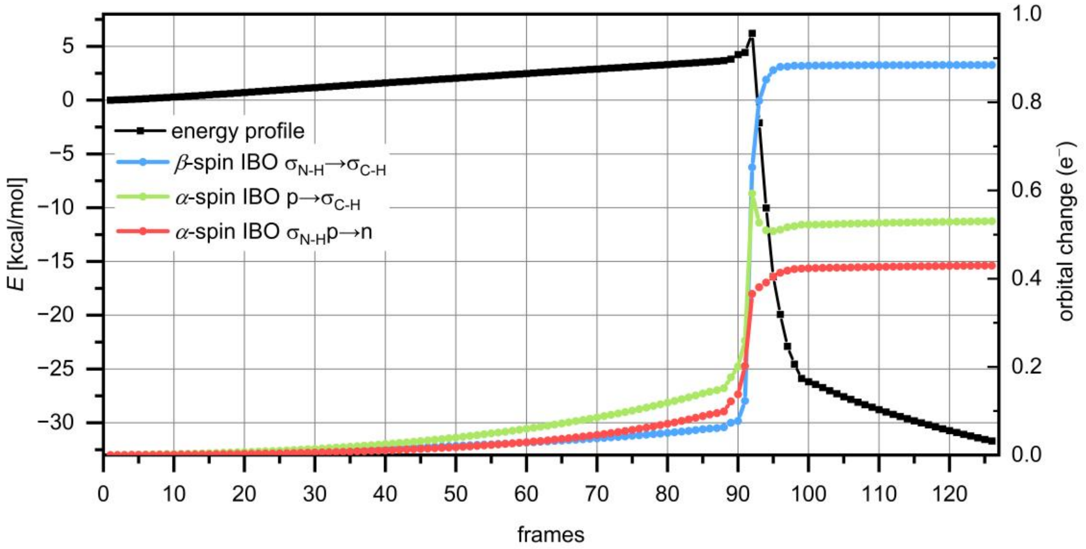

Figure S20. Intrinsic reaction coordinate (IRC) profile for the singlet pathway of the reaction of PPTH radical with phenoxy radical. Selected α- and β-spin IBOs showing significant changes along the IRC are indicated.

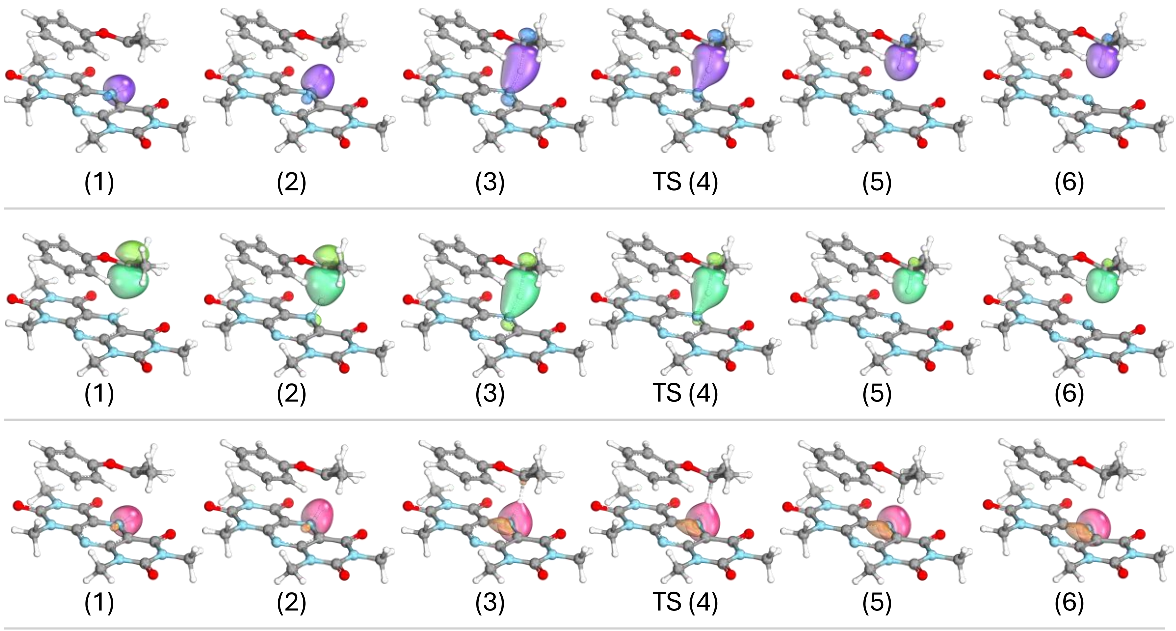

Figure S21. Representative α- and β-spin IBOs at selected points along the IRC for the PPTH–phenoxy radical reaction (singlet pathway), illustrating orbital reorganization during HAT.

10.2.2. Triplet pathway

In contrast to the singlet pathway, the reaction mechanism via the triplet pathway is substrate-dependent. For the *n*-butyl radical Figure S22A, analogously to the mechanisms discussed above for the singlet pathway, the reaction proceeds via a simultaneous transfer of a proton and a β-spin electron from the **PPTH$^{\bullet}$** radical, constituting a direct $H^{\bullet}$ transfer. Specifically, the β electron from the σ(N–H) bonding orbital is transferred into the p-orbital of the butyl radical carbon, where it pairs with the α-spin electron to form the new C–H bond. Concurrently, the α-spin electron initially residing in the σ(N–H) orbital remains localized on the **PPTH** scaffold, transiently generating a high-energy biradical intermediate. Subsequent rearomatization of the **PPT** unit restores the catalyst to its singlet ground state (S_0). The concerted nature of proton and electron transfer in this step provides unambiguous evidence for a dHAT mechanism.

In contrast, the benzyl substrate follows a fundamentally different pathway (Figure S22B). The reaction proceeds via cPCET, wherein the proton and electrons travel along distinct but coordinated trajectories. The proton is transferred directly from the N–H bond of **PPTH$^{\bullet}$** to the radical center, while the β-spin electron required for C–H bond formation originates from the substrate's aromatic π-system, facilitated by hyperconjugation and delocalization.[23] This initial electron transfer is accompanied by a second electron transfer from the **PPTH** π-system to the benzyl aromatic ring, compensating for the transient electron deficiency and stabilized by π–π interactions. This sequence of electron transfer–proton transfer–electron transfer in a concerted manner is clearly revealed by the evolution of IBOs and spin densities shown in Figure S22B, providing a compelling example of non-HAT reactivity.

An analogous ET-PT pathway is evident in the phenoxypropyl radical (Figure S22C, top). Notably, the evolution of the β-spin IBO, primarily localized at C4a and N5 of the **PPTH$^{\bullet}$**, supports a stepwise mechanism in which a second electron is transferred into the substrate's π-system after the transition state, succeeding the initial ET–PT sequence (Figure S22C, bottom).

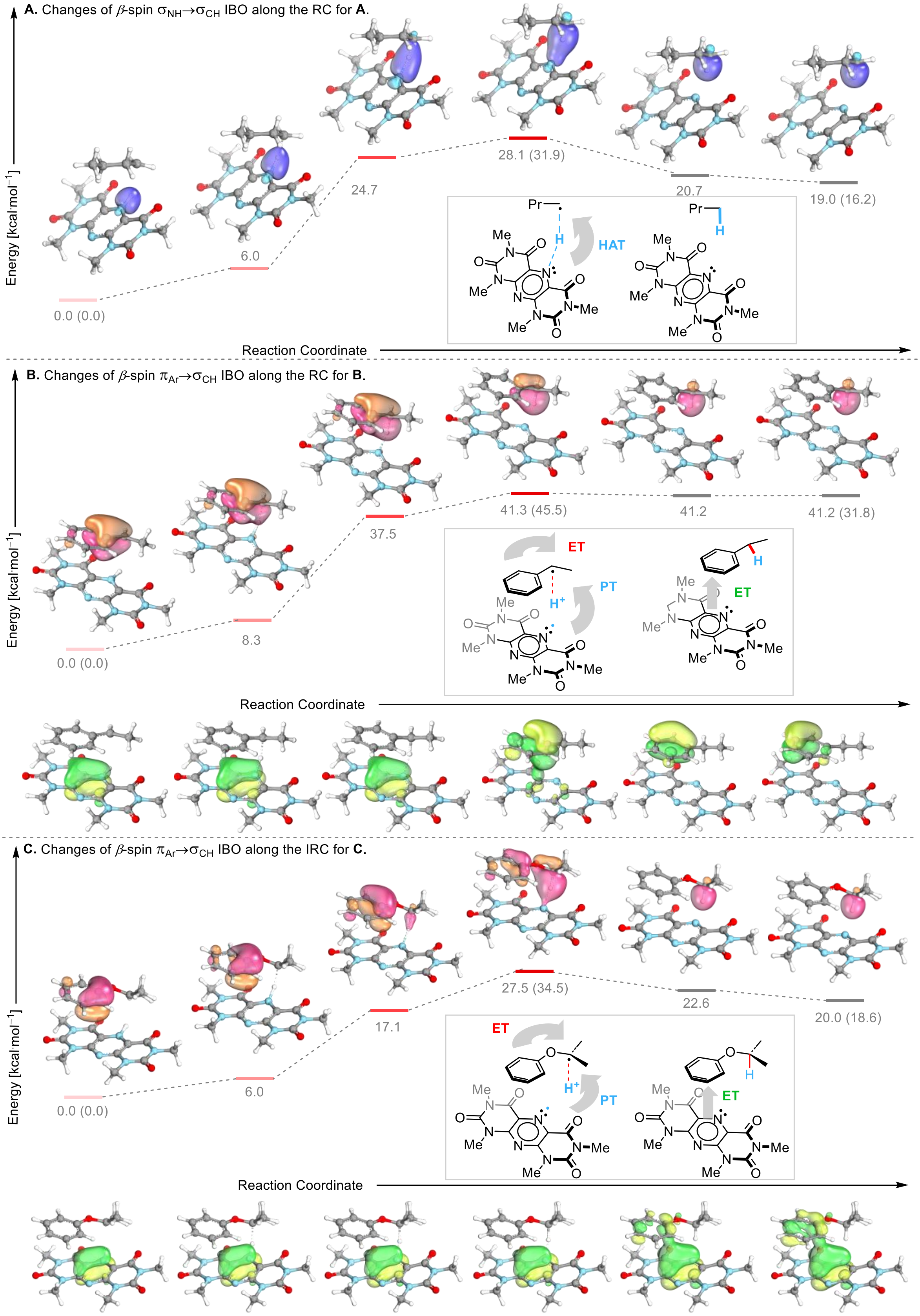

*Figure S22. IBO analysis and relative energy profiles (including free energies of stationary points) along the reaction coordinate of the **PPTH**˙ and radicals **A**, **B**, and **C**. The flow of the β-spin IBO, resembling electron transfer from π(PPT) ⟶ π(Ar), is shown for radicals **B** and **C** (green IBOs). The intensity of the reddish horizontal lines from the reactant complex to the transition state indicates the extent of out-of-plane bending, which corresponds to the π* ⟶ σ* interaction.*

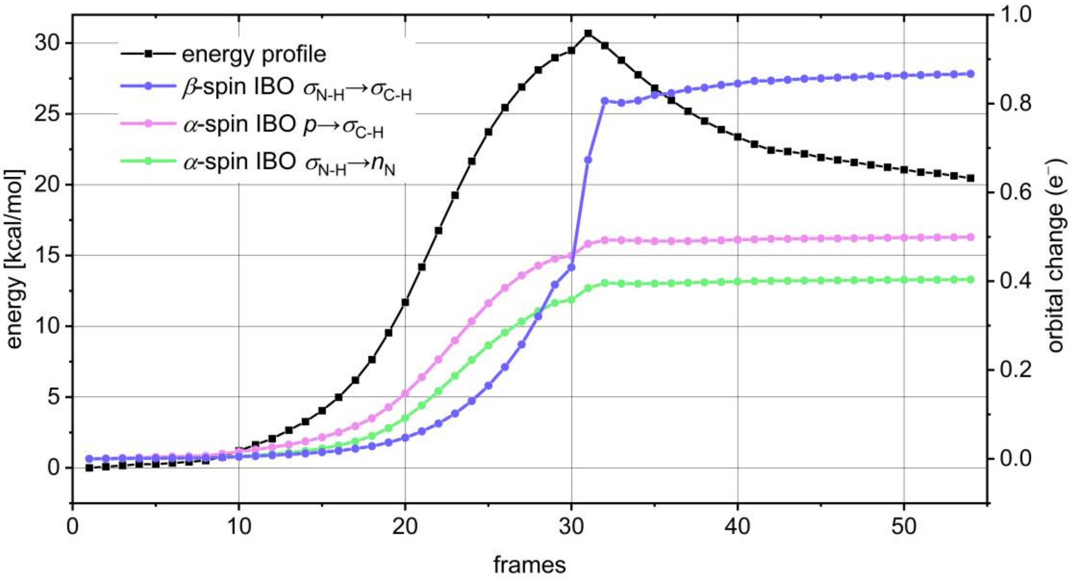

Figure S23. Intrinsic reaction coordinate (IRC) profile for the reaction of ***PPTH*** *radical with n-butyl radical. Selected α- and β-spin IBOs showing significant changes along the IRC are indicated.*

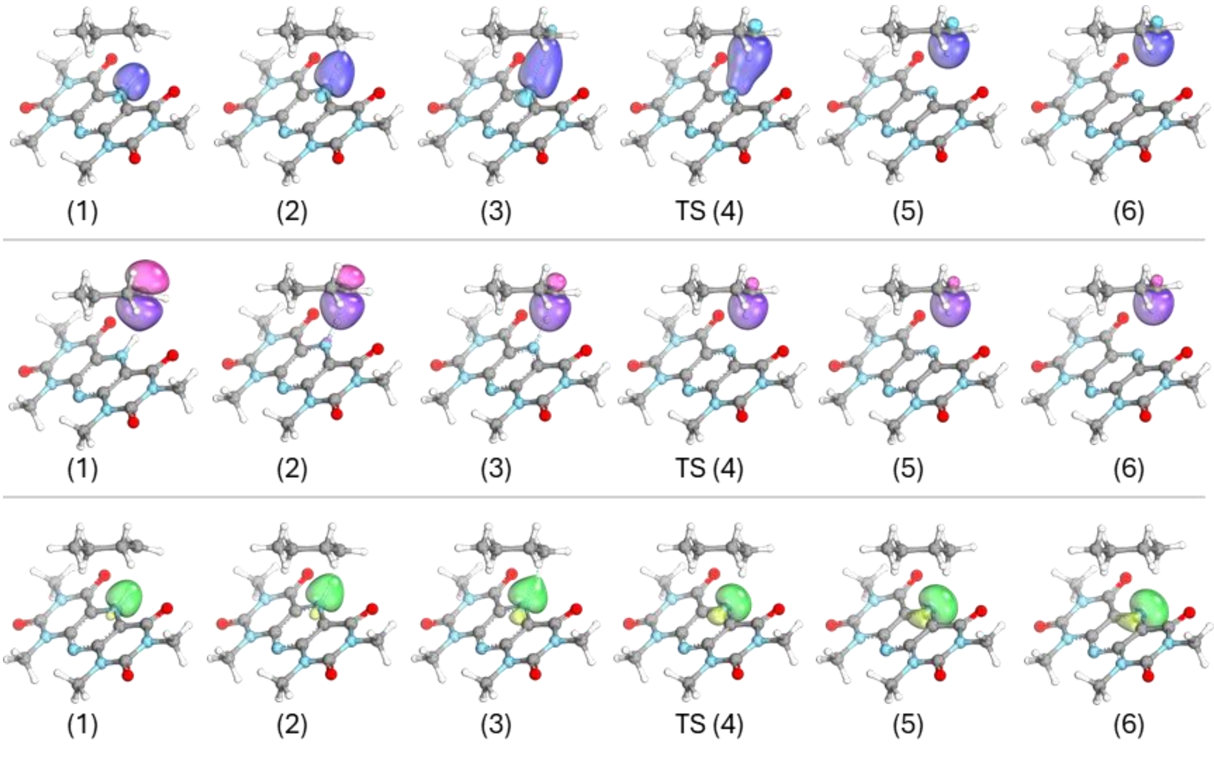

Figure S24. Representative α- and β-spin IBOs at selected points along the IRC for the ***PPTH****–n-butyl radical reaction, illustrating orbital reorganization during HAT.*

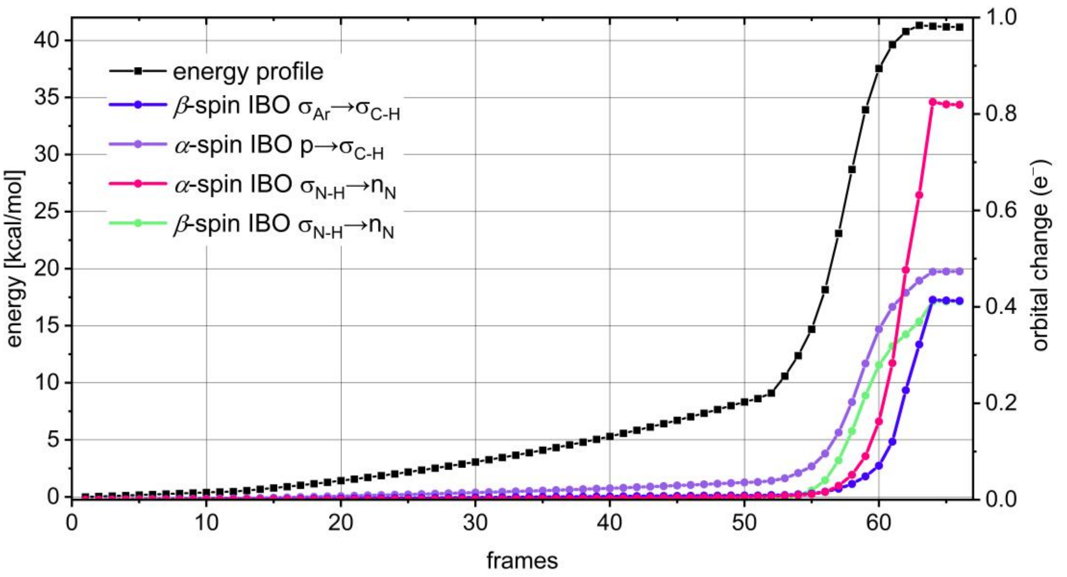

*Figure S25. IRC profile for the reaction of **PPTH** radical with benzyl radical, highlighting α- and β-spin IBOs that undergo noTable Sreorganization along the reaction path.*

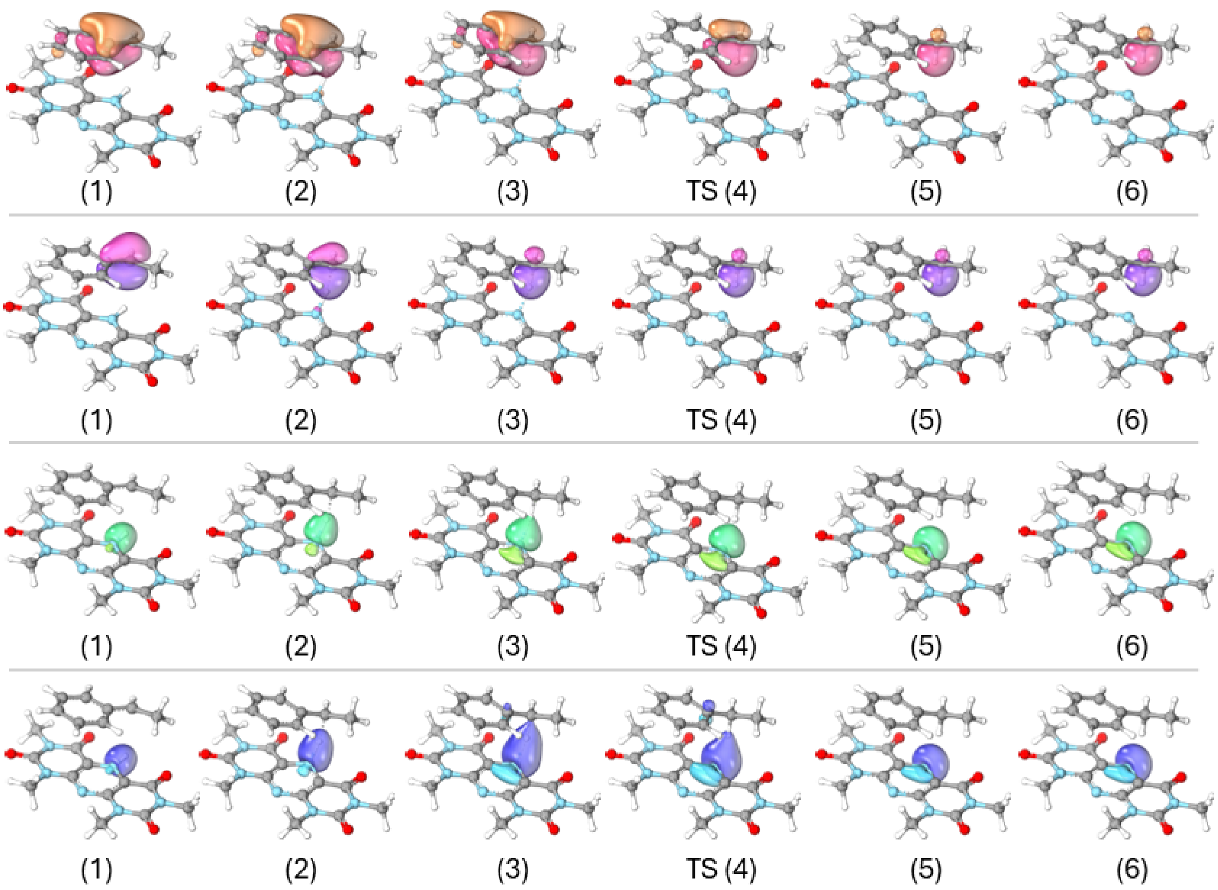

*Figure S26. Selected α- and β-spin IBOs along the IRC for the **PPTH**–benzyl radical reaction, highlighting the evolution of bonding interactions.*

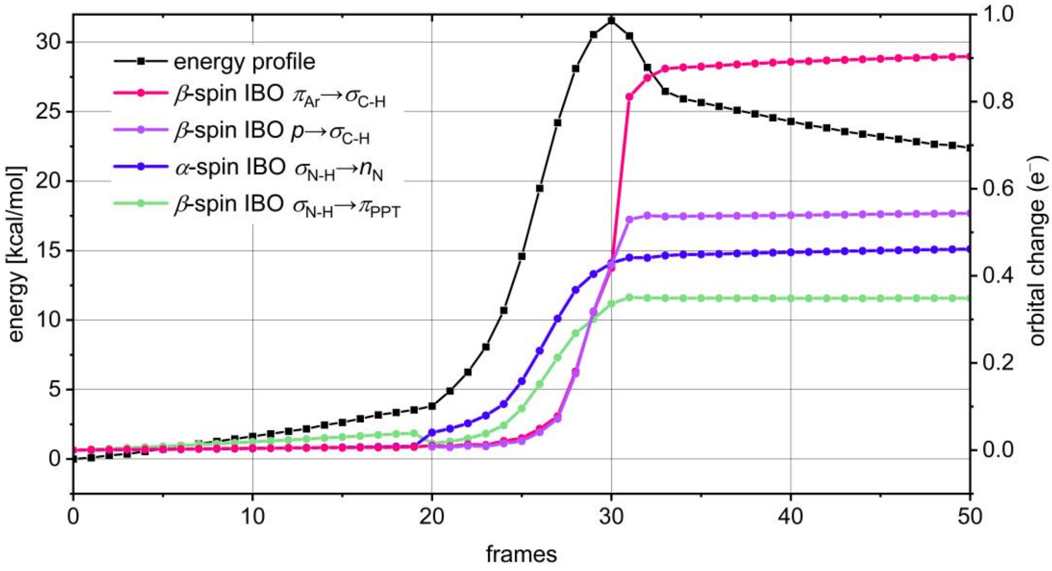

*Figure S27. IRC profile for the reaction of **PPTH** radical with phenoxy radical. Key α- and β-spin IBOs that change along the IRC are shown.*

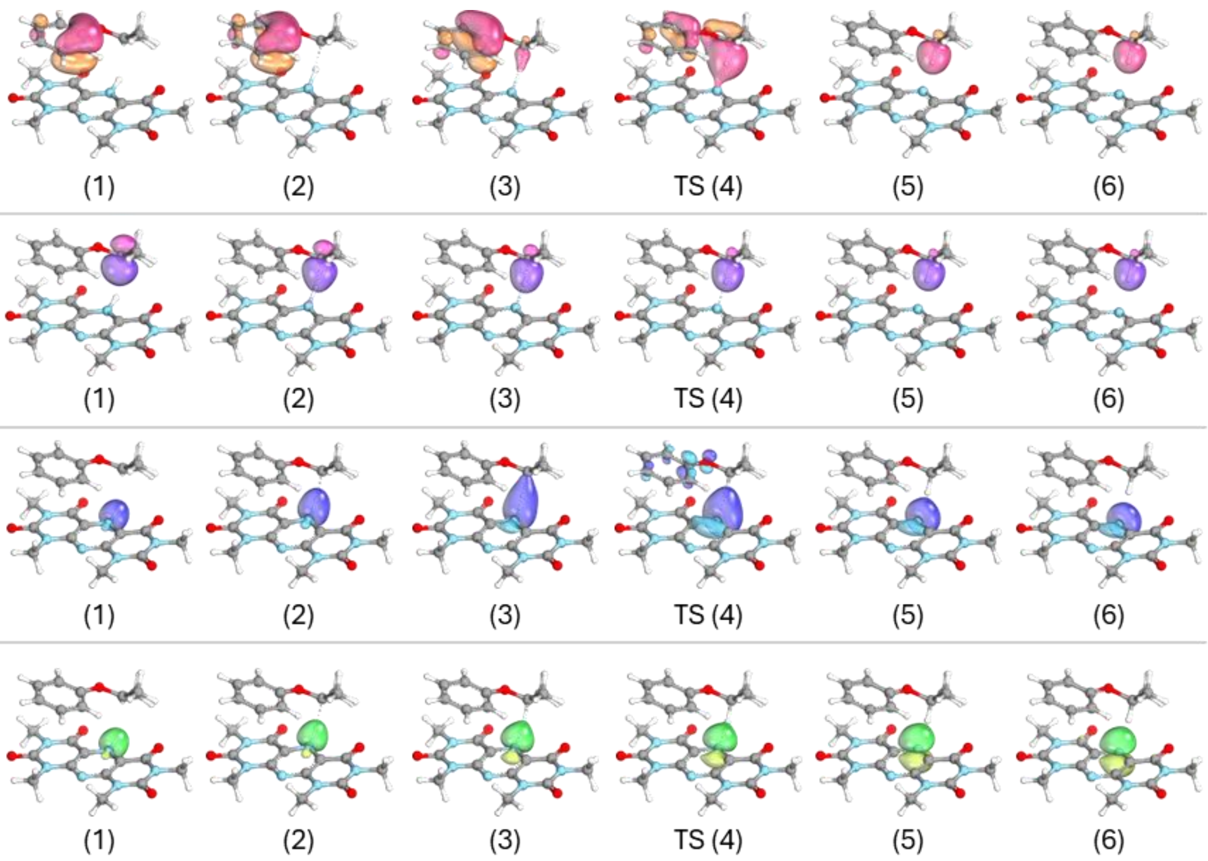

*Figure S28. α- and β-spin IBO snapshots along the IRC for the **PPTH**–phenoxy radical reaction, showing the movement and redistribution of electron density.*

11. Experimental validation of IBO analysis

To experimentally validate the mechanistic insights obtained from IBO analysis, we re-examined the thermodynamic parameters of representative carbon-centered radicals (Table S10) and correlated them with reactivity patterns observed in photomediated protodecarboxylation.

Table S10. Thermodynamic parameters of carboxylic acids and carbon-centered radicals.

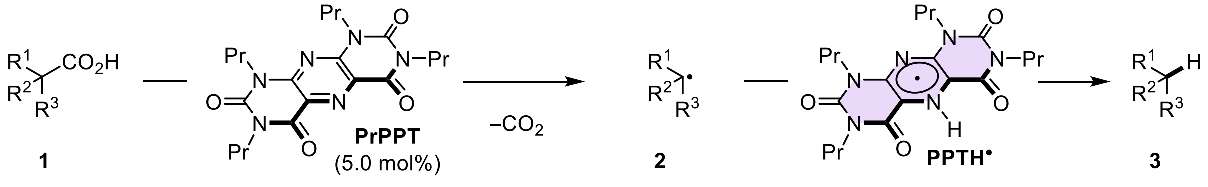

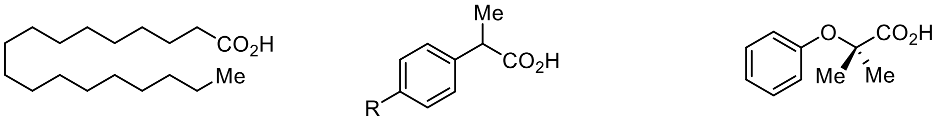

1a **1b** (R = H) **1d** (R = Cl) **1c**

entry	compound	pK_a[a]	E_{ox} [eV][b]		ΔG°_{ET}[c] [kcal·mol⁻¹]		ΔBDFE[d] [kcal·mol⁻¹]	E_{red} [V][b]	ΔG°_{ET}[e] [kcal·mol⁻¹]	ΔBDFE[f] [kcal·mol⁻¹]
			RCO_2H	RCO_2^-	RCO_2H	RCO_2^-		R•		
1	**1a**	21.2	>+3.0	>+3.0	+43	+43	–2	–2.0	+47	–34
2	**1b**	21.1	+2.40	+1.29	+29	+3	–3	–1.34[24,25]	+21	–22
3	**1c**	18.8	+2.08	+1.26	+21	+3	–3	+0.15[25,f]	–3	–28

[a] in MeCN. Calculated from pK_a values in water using pKa(MeCN) =(pKa(H_2O)+X+CO · 2.2–nC · 0.13–MW · 0.0017+6.5)/0.55.[26] [b] referenced to NHE by adding +0.63 V to the value measured against Fc^+/Fc. [c] From $\Delta G^\circ = (E_{ox,\ Sub} - {}^3E_{red,\ PPT*})\cdot 23.06$ in kcal·mol⁻¹. [d] ΔBDFE = $BDFE_{O\text{-}H}$–$BDFE*_{PPTH\bullet}$ in kcal·mol⁻¹. BDFEs were calculated using the ALFABET BDE predictor.[27] [e] From $\Delta G^\circ = (E_{red,\ PPT} - E_{red,\ Sub})\cdot 23.06$ in kcal·mol⁻¹. [f] ΔBDFE = $BDFE_{O\text{-}H}$–$BDFE*_{PPTH\bullet}$ in kcal·mol⁻¹. From BDFE values for C–H bonds.[27]

12.1. Photo-mediated protodecarboxylation of carboxlic acids 1a-1d

All reactions were performed on a 0.25 mmol or 0.5 mmol scale and a molarity of 0.1 M under an inert atmosphere according to the general procedure previously reported by our group.[28] Unless otherwise stated, yields are measured via calibrated GC using biphenyl as internal standard. The reactions were irradiated using 2 x 30 W LED with an emission maxima at 396 nm. The reaction was submerged in a translucent oil bath heated to 50 °C.

Determination of Yields by GC-FID Analysis

Reaction yields were determined by GC-FID using biphenyl as an internal standard (ISTD).

After completion of the reaction, the mixture was allowed to cool to room temperature. An accurately weighed amount of biphenyl (38.55 mg, 0.25 mmol) was added to the crude reaction mixture. The mixture was diluted with ethyl acetate (2 mL), filtered through a short plug of celite, and analyzed by GC-FID.

GC Conditions

GC-FID analyses were performed on an instrument equipped with a capillary column (30 m × 0.25 mm × 0.25 µm film thickness). The injector temperature was set to 250 °C and the detector temperature to 280 °C. The oven temperature program was: 80 °C (2 min), ramped at 15 °C/min to 280 °C, held for 5 min. Nitrogen was used as carrier gas. Injection volume: 1.0 µL (split ratio 20:1).

Calibration and Response Factor Determination

Response factors (RF) were determined using independently prepared calibration mixtures containing known molar ratios of the product and internal standard. Calibration solutions were prepared in ethyl acetate with product-to-standard molar ratios of 2.0:1.0, 1.6:1.0, 1.2:1.0, 0.8:1.0, and 0.4:1.0. Each mixture was analyzed in triplicate.

The response factor (RF) was calculated according to:

$$RF = \frac{(A_{prod}/n_{prod})}{(A_{std}/n_{std})}$$

where

- A_{prod} = integrated peak area of the product
- A_{std}= integrated peak area of biphenyl
- n_{prod} = number of moles of product
- n_{std}= number of moles of biphenyl

The average response factor obtained from three calibration points was used for yield calculations.

All measurements were performed in triplicate, and the resulting calibration curve was used for the quantification of product in the reaction mixtures.

Yield Calculation

The amount of product formed in the reaction was calculated using:

$$n_{prod} = \frac{A_{prod} \cdot n_{ISTD}}{A_{std} \cdot RF}$$

The GC yield (%) was determined relative to the initial amount of limiting reagent:

$$Yield\ (\%) = \frac{n_{prod}}{n_{carboxylic\ acid}} \cdot 100$$

All reported GC yields represent the average of at least two independent measurements.

*Table S11. . Screening of different solvents for the base-free protodecarboxylation of stearic acid (**1a**) at 50 °C.*

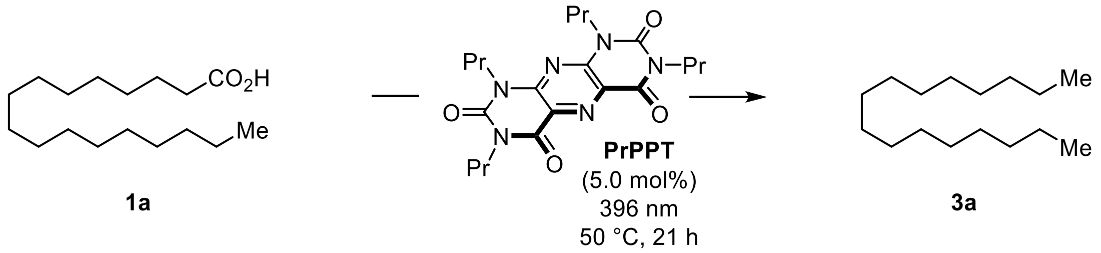

Entry	Variations	Yield [%][a]
1	EtOAc (25%) MeCN/ H_2O (9:1)[b]	18%
2	EtOAc (25%) MeCN/ H_2O (9:1)	10%
3	MeCN/EtOAc (1:1)	5%
4	EtOAc	6%
5	MeCN/EtOAc (3:1)	5%
6	MeCN	<5%[c]

[a] Reactions were conducted on a 0.5 mmol scale. Yields were determined by calibrated GC using biphenyl as internal standard. [b] 1.33 equiv. of base were added. [c] **1a** insoluble in MeCN.

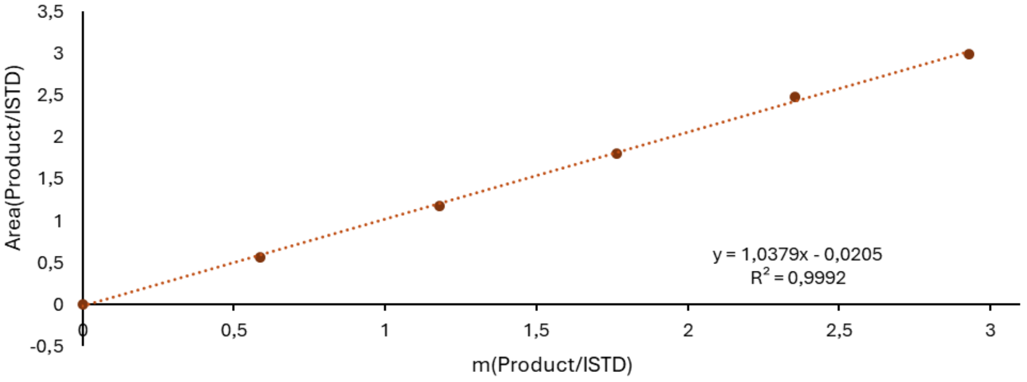

*Figure S29. Calibration curve for stearic acid (**1a**). Biphenyl was used as internal standard.*

Note: 2-(4-chlorophenyl)propanoic acid (**1d**) was used instead of 2-phenylpropanoic acid (**1b**) due to improved detectability of the corresponding decarboxylation product (**3d**).

*Table S12. Protodecarboxylation of 4-chlorophenylacetic acid (**1d**) at 50 °C.*

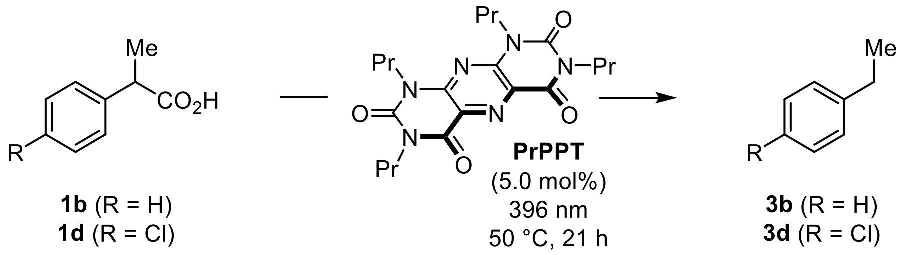

Entry	Compound	Variations	Yield [%][a]
1	**1d**	MeCN/H_2O (9:1)[b]	77%[c]
2	**1d**	MeCN	21%

[a] Reactions were conducted on a 0.5 mmol scale. Yields were determined by calibrated GC using biphenyl as internal standard. [b] 1.33 equiv. of base were added. Water was added for solubility. [c] Isolated yield.

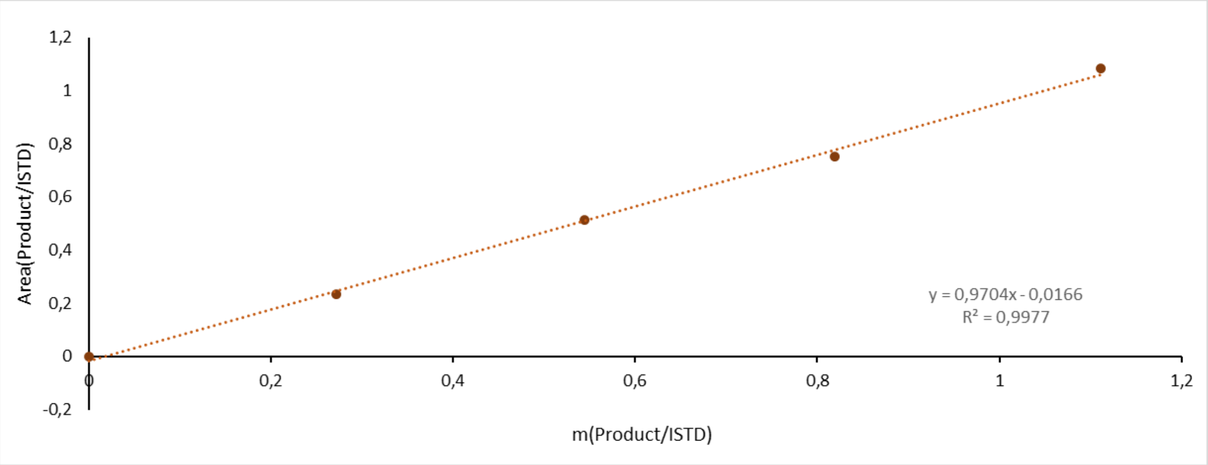

*Figure S30. Calibration curve for 2-(4-chlorophenyl)propanoic acid (**1d**). Biphenyl was used as internal standard.*

*Table S13. Protodecarboxylation of 2-methyl-2-phenoxypropanoic acid (**1c**) at 50 °C.*

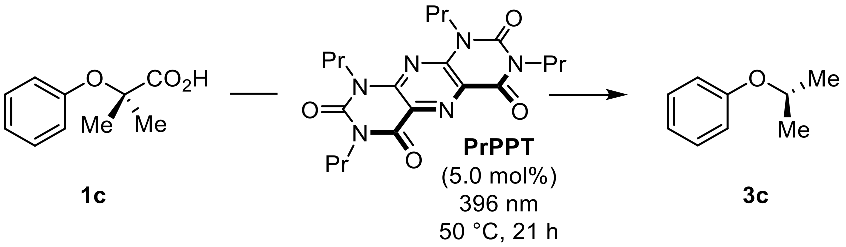

Entry	Compound	Variations	Yield [%][a]
1	**1c**	MeCN/H_2O (9:1)[b]	35%[c]
2	**1c**	MeCN	<5%

[a] Reactions were conducted on a 0.5 mmol scale. [b] 1.33 equiv. of base were added. Water was added for solubility. [c] NMR yield using 1,3,5-trimethoxybenzene as internal standard.

11.2. Capture of carbanion intermediates

To probe the involvement of carbanion intermediates, we employed acetone (3.0 equiv.) as a trapping reagent, anticipating formation of 1,2-addition products that would provide direct experimental evidence for species **4a-d**. The experimental results are summarized in Table S14, below.

Table S14. Trapping of Carbanion Intermediates with Acetone.

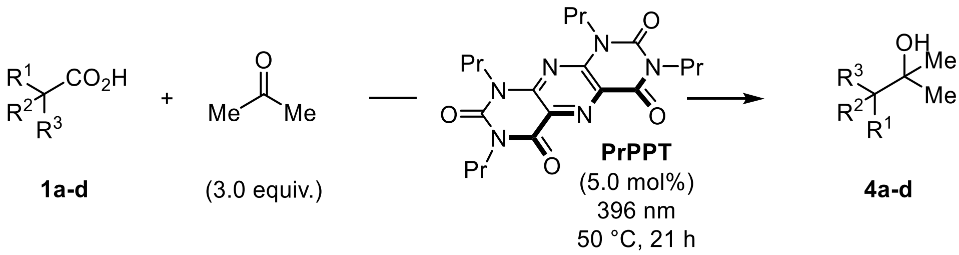

Entry	Compound	Variations	result[b]
1	**1a**	MeCN	n.d.[c]
2	**1b**	MeCN	n.d.[c]
3	**1c**	MeCN	n.d.[c]
4	**1d**	MeCN	positive

[a] Reactions were conducted on a 0.5 mmol scale. [b] Qualitative assessment by GC, GC-MS and LC-MS analysis. Unoptimzed conditions. [c] not detected (n.d.).

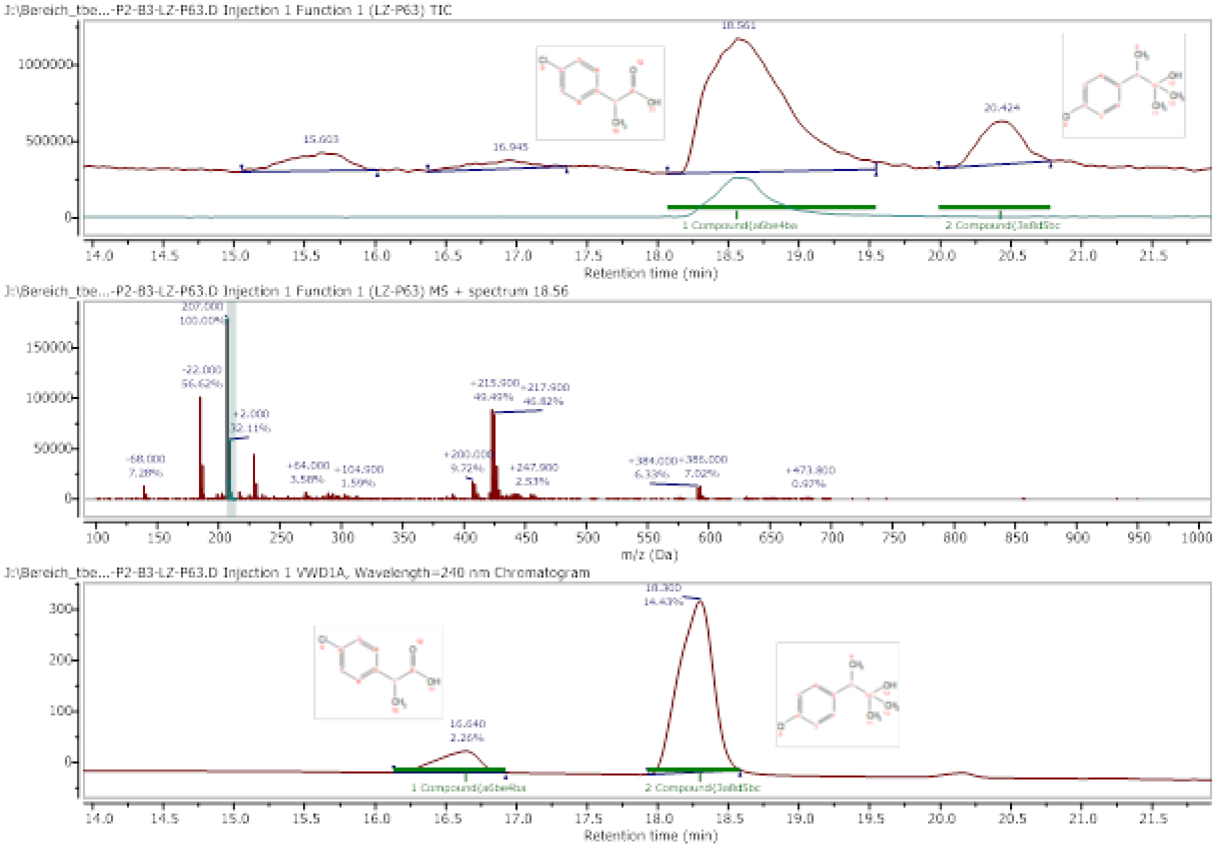

Figure S31. LC-MS analysis of 1,2-addition product ***4d.***

12. Abbreviations

Table S15. List of abbreviations used in this work.

Abbreviation	*Meaning*
PPT	*Pyrimidopteridinetetraones*
PPTH˙	*N-hydropyrimidopteridinetetraone radicals*
PrPPT	*Tetrapropylpyrimidopteridinetetraone*
HAT	*Hydrogen Atom Transfer*
dHAT	*direct HAT*
PCET	*Proton-Coupled Electron Transfer*
SET	*Single-electron transfer*
ET-PT	*step-wise Electron Transfer/Proton Transfer*
BDFE	*Bond Dissociation Free Energies*
EPR	*Electron Paramagnetic Resonance*
CV	*Cyclic Voltammetry*
DPV	*Differential pulse voltammetry*
(TD)DFT	*(Time-Dependent)Density Functional Theory*
IBO	*Intrinsic bond orbital*
IRC	*Intrinsic Reaction Coordinate*
NTO	*Natural Transition Orbitals*
HONTO	*Highest Occupied NTO*
LUNTO	*Lowest Unoccupied NTO*
ISC	*Intersystem-Crossing*
VR	*Vibrational Relaxation*
CT	*Charge Transfer*
IC	*Internal Conversion*
SOC	*Spin–Orbit Coupling*
NBO	*Natural Bond Orbital*
SR	*Seperated Reactants*
RC	*Reactant Complex*
TS	*Transition State*
PR	*Product*

13. References

(1) Taeufer, T.; Argüello Cordero, M. A.; Petrosyan, A.; Surkus, A.-E.; Lochbrunner, S.; Pospech, J. Photophysical and Electrochemical Properties of Pyrimidopteridine-Based Organic Photoredox Catalysts. *ChemPhotoChem* **2021**, *5* (11), 999–1003. DOI: https://doi.org/10.1002/cptc.202100159.

(2) Yanai, T.; Tew, D. P.; Handy, N. C. A new hybrid exchange–correlation functional using the Coulomb-attenuating method (CAM-B3LYP). *Chem. Phys. Lett.* **2004**, *393* (1), 51–57. DOI: https://doi.org/10.1016/j.cplett.2004.06.011.

(3) Iikura, H.; Tsuneda, T.; Yanai, T.; Hirao, K. A long-range correction Scheme Sfor generalized-gradient-approximation exchange functionals. *J. Chem. Phys.* **2001**, *115* (8), 3540–3544. DOI: 10.1063/1.1383587.

(4) Weigend, F.; Ahlrichs, R. Balanced basis sets of split valence, triple zeta valence and quadruple zeta valence quality for H to Rn: Design and assessment of accuracy. *Physical Chemistry Chemical Physics* **2005**, *7* (18), 3297–3305, 10.1039/B508541A. DOI: 10.1039/B508541A.

(5) Grimme, S.; Ehrlich, S.; Goerigk, L. Effect of the damping function in dispersion corrected density functional theory. *J. Comput. Chem.* **2011**, *32* (7), 1456–1465. DOI: https://doi.org/10.1002/jcc.21759.

(6) Barone, V.; Cossi, M. Quantum Calculation of Molecular Energies and Energy Gradients in Solution by a Conductor Solvent Model. *J. Phys. Chem. A* **1998**, *102* (11), 1995–2001. DOI: 10.1021/jp9716997.

(7) Tomasi, J.; Mennucci, B.; Cammi, R. Quantum Mechanical Continuum Solvation Models. *Chem. Rev.* **2005**, *105* (8), 2999–3094. DOI: 10.1021/cr9904009.

(8) Fukui, K. The path of chemical reactions - the IRC approach. *Acc. Chem. Res.* **1981**, *14* (12), 363–368. DOI: 10.1021/ar00072a001.

(9) Fukui, K. Formulation of the reaction coordinate. *J. Phys. Chem.* **1970**, *74* (23), 4161–4163. DOI: 10.1021/j100717a029.

(10) Knizia, G. Intrinsic Atomic Orbitals: An Unbiased Bridge between Quantum Theory and Chemical Concepts. *Journal of Chemical Theory and Computation* **2013**, *9* (11), 4834–4843. DOI: 10.1021/ct400687b.

(11) Knizia, G.; Klein, J. E. M. N. Electron Flow in Reaction Mechanisms—Revealed from First Principles. *Angew. Chem. Int. Ed.* **2015**, *54* (18), 5518–5522. DOI: https://doi.org/10.1002/anie.201410637.

(12) http://www.iboview.org

(13) Reed, A. E.; Weinstock, R. B.; Weinhold, F. Natural population analysis. *J. Chem. Phys.* **1985**, *83* (2), 735–746. DOI: 10.1063/1.449486.

(14) Plasser, F. TheoDORE: A toolbox for a detailed and automated analysis of electronic excited state computations. *J. Chem. Phys.* **2020**, *152* (8). DOI: 10.1063/1.5143076.

(15) Mai, S.; Plasser, F.; Dorn, J.; Fumanal, M.; Daniel, C.; González, L. Quantitative wave function analysis for excited states of transition metal complexes. *Coord. Chem. Rev.* **2018**, *361*, 74–97. DOI: https://doi.org/10.1016/j.ccr.2018.01.019.

(16) Neese, F.; Wennmohs, F.; Becker, U.; Riplinger, C. The ORCA quantum chemistry program package. *J. Chem. Phys.* **2020**, *152* (22). DOI: 10.1063/5.0004608.

(17) Pavlishchuk, V. V.; Addison, A. W. Conversion constants for redox potentials measured versus different reference electrodes in acetonitrile solutions at 25°C. *Inorg. Chim. Acta* **2000**, *298* (1), 97–102. DOI: https://doi.org/10.1016/S0020-1693(99)00407-7.

(18) Raamat, E.; Kaupmees, K.; Ovsjannikov, G.; Trummal, A.; Kütt, A.; Saame, J.; Koppel, I.; Kaljurand, I.; Lipping, L.; Rodima, T.; et al. Acidities of strong neutral Brønsted acids in different media. *J. Phys. Org. Chem.* **2013**, *26* (2), 162–170. DOI: https://doi.org/10.1002/poc.2946.

(19) Kütt, A.; Leito, I.; Kaljurand, I.; Sooväli, L.; Vlasov, V. M.; Yagupolskii, L. M.; Koppel, I. A. A Comprehensive Self-Consistent Spectrophotometric Acidity Scale of Neutral Brønsted Acids in Acetonitrile. *J. Org. Chem.* **2006**, *71* (7), 2829–2838. DOI: 10.1021/jo060031y.

(20) Agarwal, R. G.; Coste, S. C.; Groff, B. D.; Heuer, A. M.; Noh, H.; Parada, G. A.; Wise, C. F.; Nichols, E. M.; Warren, J. J.; Mayer, J. M. Free Energies of Proton-Coupled Electron Transfer Reagents and Their Applications. *Chem. Rev.* **2022**, *122* (1), 1–49. DOI: 10.1021/acs.chemrev.1c00521.

(21) Yamaguchi, K.; Jensen, F.; Dorigo, A.; Houk, K. N. A spin correction procedure for unrestricted Hartree-Fock and Møller-Plesset wavefunctions for singlet diradicals and polyradicals. *Chem. Phys. Lett.* **1988**, *149* (5), 537–542. DOI: https://doi.org/10.1016/0009-2614(88)80378-6.

(22) Yamaguchi, K.; Takahara, Y.; Fueno, T. Ab-Initio Molecular Orbital Studies of Structure and Reactivity of Transition Metal-OXO Compounds. In *Applied Quantum Chemistry*, Dordrecht, 1986//, 1986; Smith, V. H., Schaefer, H. F., Morokuma, K., Eds.; Springer Netherlands: pp 155–184. DOI: 10.1007/978-94-009-4746-7_11.

(23) Van Hoomissen, D. J.; Vyas, S. Impact of Conjugation and Hyperconjugation on the Radical Stability of Allylic and Benzylic Systems: A Theoretical Study. *J. Org. Chem.* **2017**, *82* (11), 5731–5742. DOI: 10.1021/acs.joc.7b00549.

(24) Sim, B. A.; Griller, D.; Wayner, D. D. M. Reduction potentials for substituted benzyl radicals: pKa values for the corresponding toluenes. *J. Am. Chem. Soc.* **1989**, *111* (2), 754–755. DOI: 10.1021/ja00184a066.

(25) Fu, Y.; Liu, L.; Yu, H.-Z.; Wang, Y.-M.; Guo, Q.-X. Quantum-Chemical Predictions of Absolute Standard Redox Potentials of Diverse Organic Molecules and Free Radicals in Acetonitrile. *J. Am. Chem. Soc.* **2005**, *127* (19), 7227–7234. DOI: 10.1021/ja0421856.

(26) Kütt, A.; Tshepelevitsh, S.; Saame, J.; Lõkov, M.; Kaljurand, I.; Selberg, S.; Leito, I. Strengths of Acids in Acetonitrile. *Eur. J. Org. Chem.* **2021**, *2021* (9), 1407–1419. DOI: https://doi.org/10.1002/ejoc.202001649.

(27) St. John, P. C.; Guan, Y.; Kim, Y.; Kim, S.; Paton, R. S. Prediction of organic homolytic bond dissociation enthalpies at near chemical accuracy with sub-second computational cost. *Nature Communications* **2020**, *11* (1), 2328. DOI: 10.1038/s41467-020-16201-z.

(28) Mayer, T. S.; Taeufer, T.; Brandt, S.; Rabeah, J.; Pospech, J. Photomediated Hydro- and Deuterodecarboxylation of Pharmaceutically Relevant and Natural Aliphatic Carboxylic Acids. *J. Org. Chem.* **2023**, *88* (10), 6347–6353. DOI: 10.1021/acs.joc.2c01664.
